# Body mass index in parents and their adult offspring: A systematic review and meta‐analysis

**DOI:** 10.1111/obr.13644

**Published:** 2023-10-02

**Authors:** Jie Zhang, Gemma L. Clayton, Kim Overvad, Anja Olsen, Deborah A. Lawlor, Christina C. Dahm

**Affiliations:** ^1^ Department of Public Health Aarhus University Aarhus Denmark; ^2^ Population Health Sciences, Bristol Medical School University of Bristol Bristol UK; ^3^ MRC Integrative Epidemiology Unit at the University of Bristol Bristol UK; ^4^ Danish Cancer Society Research Center Copenhagen Denmark

**Keywords:** adult offspring, body mass index, intergenerational, systematic review

## Abstract

Obesity may track across generations, due to genetics and shared family environmental factors, or possibly intrauterine programming. However, many studies only assess associations between maternal body mass index (BMI) and offspring BMI in childhood. To determine whether maternal and paternal associations with offspring BMI differ and whether associations persist into adulthood, a systematic review and meta‐analysis was done. PubMed, Embase, Web of Science, and Google Scholar (to October 2022) were searched. Observational studies reporting associations between maternal or paternal BMI and adult offspring BMI were included. Offspring BMIs were reported as continuous or categorical measures. Forty‐six studies were included in the systematic review. Meta‐analyses were conducted using random‐effects models. Parental BMI was positively associated with offspring BMI in adulthood. The pooled mother–offspring standardized mean difference (SMD) was 0.23 (95% confidence interval [CI]: 0.20, 0.26), and father–offspring SMD was similar: 0.22 (95% CI: 0.19, 0.25) in adjusted models. Offspring of mothers with overweight or obesity had the same risk of higher BMI as offspring of fathers with overweight or obesity. If these associations are causal, they support interventions targeting all family members, rather than focusing solely on mothers, to obtain a healthy weight development among offspring.

**Abbreviations**
BMI: body mass index
CI: confidence interval
MD: mean difference
OR: odds ratio
ROR: ratio of odds ratio
RR: risk ratio
SMD: standardized mean difference

## 1 INTRODUCTION

Obesity represents one of the most urgent national and global health challenges because of its high prevalence and long‐term adverse health consequences.^1^ Familial resemblance in body mass index (BMI) is well recognized.^2^ To date, multiple studies have explored parental–offspring BMI associations and linked adiposity in parents with unfavorable offspring body composition and related morbidity (e.g., diabetes, cardiovascular and metabolic diseases).^3^, ^4^

The causes of the intergenerational association are unclear. Genetic predisposition, family socioeconomic status, and lifestyle factors (e.g., diet, physical activity) have been implicated.^5^, ^6^ In addition to the above factors, mothers with a higher BMI during pregnancy might increase the risk of their offspring becoming overweight via the fetal environment according to the “fetal overnutrition” hypothesis.^7^, ^8^ The hypothesis suggests that maternal nutritional imbalance during gestation can alter metabolic processes, including the hypothalamic response to leptin and subsequent regulation of appetite and pancreatic beta‐cell physiology, and have a persistent effect on offspring adiposity development.^9^ This hypothesis is supported by some animal studies,^10^, ^11^ yet the evidence is mixed in human studies.^12^, ^13^ However, novel evidence from Mendelian randomization studies that use maternal genetic variants adjusted for offspring genetic variants as unconfounded instrumental variables for an intrauterine exposure casts doubt on the causal effect of maternal pregnancy adiposity.^14^, ^15^ Furthermore, “negative control” studies, which used paternal adiposity as a control and compared mother–offspring and father–offspring BMI associations suggest that maternal associations may be explained by family confounding.^6^, ^16^ If there is a causal in utero effect, the association between parental and offspring BMI should be stronger in the maternal line than in the paternal line.^17^ There is also growing interest to investigate whether any such association differs between daughters and sons. Comparison between the same‐sex (mother–daughter, father–son) versus cross‐sex (mother–son, father–daughter) transmission would provide more insight into understanding the mechanisms underlying the association. If a stronger same‐sex association is observed, it might imply that shared environment rather than genes is the driver because selective mother–daughter and father–son gene transmission is not a common Mendelian trait.^18^ However, no consistent patterns have been identified so far, making it difficult to draw conclusions. While previous systematic reviews have assessed associations between parental BMI and BMI of the offspring during childhood,^6^, ^19^ few have investigated sex‐specific parental–offspring BMI associations, and very little is known about the strength of associations with offspring in adulthood. We undertook this systematic review and meta‐analyses to quantify the strengths of associations between parental BMI and that of their adult offspring, separately by maternal and paternal lines. The objectives of the present study were threefold: (i) to synthesize data from studies published from 1980 to 2022, thus investigating whether parental BMI is persistently associated with offspring BMI in adulthood; (ii) to explore the difference in maternal or paternal associations with adult offspring BMI and compare the associations for maternal and paternal lines; and (iii) to assess same‐sex (mother–daughter, father–son) and cross‐sex (mother–son, father–daughter) parent‐offspring BMI associations.

## 2 METHODS

The meta‐analysis was reported according to the Meta‐analysis of Observational Studies in Epidemiology (MOOSE) checklists.^20^ The study was registered in PROSPERO (CRD42020159281), an international database of prospectively registered systematic reviews.

### 2.1 Search strategy

We carried out a systematic literature search of PubMed, Embase, Web of Science, and Google Scholar using predefined search terms (Appendix in the Supporting Information). Searches were restricted to human studies published in English, full‐text articles published after 1980. The reference lists of all studies that met the inclusion criteria and all related systematic reviews were searched by J.Z., H.T.V., and C.C.D. Citation searches for all studies that met the inclusion criteria and all related systematic reviews were performed using Google Scholar Citations. Authors of included studies were contacted for additional data when required for inclusion in the meta‐analyses. Database searches were completed in August 2019 and updated in October 2022.

### 2.2 Study eligibility criteria

#### 2.2.1 Inclusion criteria

Inclusion criteria were peer‐reviewed studies reporting both the exposure variable (maternal BMI or paternal BMI) and the outcome variable (offspring BMI) among offspring aged 18 years or older. Exposure and outcome variables could be either continuous or categorical. To be included, studies must have been published as full texts written in English, and the findings expressed as correlation or regression coefficients, or odds ratios (OR) or risk ratios (RR) with some measure of variability such as 95% confidence intervals (CIs) or standard errors. Studies reporting offspring BMI in late adolescence/early adulthood (reporting any offspring aged 18 years or older) were included. In cohort studies that looked at three generations, only the first two generations, that is, grandparents and parents, were included when the youngest (third) generation was less than 18 years of age. We reported offspring BMI as a continuous measure as the primary outcome. Where studies were based on the same cohort (e.g., 1958 British birth cohort) and the same participants, we chose the study with the larger study population or older age group.

#### 2.2.2 Study selection

Three assessors (J.Z., H.T.V., M.L.L.) independently reviewed the titles and abstracts of all identified citations using Covidence online software (Covidence.org).^21^ Each full‐text article was independently evaluated by three reviewers (J.Z., C.C.D., H.T.V.). Disagreements were settled by discussion and consensus, with the third reviewer (C.C.D.) available as an adjudicator.

#### 2.2.3 Data extraction

A piloted data collection form was used by three reviewers (J.Z., C.C.D., H.T.V.) to independently extract the following data from full‐text articles: study design, years of study, study origin (country), study setting, sample size, assessment methods of weight and height information, when BMI was ascertained, whether maternal BMI pertained to pre‐pregnancy, and reported measures of association. We included information available from the publications. Inconsistencies were checked and resolved through the consensus process described earlier.

### 2.3 Data synthesis

#### 2.3.1 Primary outcome (difference in mean BMI)

We extracted correlation coefficients (*r*) or regression coefficients (mean difference [MD] or standardized mean difference [SMD]) for continuous outcomes. As studies varied in reporting associations between different familial groupings, we summarized two levels of family relationship groups: (i) sex‐specific at parental level and offspring level: mother–daughter, mother–son, father–daughter, and father–son; (ii) sex‐specific at parental level: mother–offspring and father–offspring. When measures of association were reported by offspring sex at parental and offspring level, we used methods proposed by Borenstein et al^22^ to combine father–offspring (or mother–offspring) from sex‐specific associations (father–daughter and father–son, or mother–daughter and mother–son) and assumed varying correlations (ranging from 0 to 0.3) depending on the study design and analysis of the study to account for dependency. For example, we assumed a correlation of 0.3 where multilevel models had accounted for the structure of the data while we assumed a correlation of 0 in models with, for example, fathers with only one child. An inverse variance‐weighted fixed‐effects model was used to pool the sex‐specific associations to either father–offspring or mother–offspring. Finally, a random‐effects meta‐analysis was used to synthesize the overall parental–offspring association to help account for between study differences. We therefore report the average association based on the distribution of study estimates while also describing any between‐study heterogeneity.^23^

For data from regression models (where offspring BMI could be in kg/m^2^ or SD units), we separately extracted regression coefficients that were (i) MD per kg/m^2^ difference in parental BMI and (ii) SMD per SD difference in parental BMI.^24^, ^25^, ^26^, ^27^ We then transformed studies reporting MD per kg/m^2^ difference in parental BMI to SD units so that (i) and (ii) could be combined to increase power. Regression coefficients from multilevel models were treated as standard regression models. For studies reporting correlation coefficients (*r*), data were transformed with Fisher's Z scale, and via the inverse Fisher's Z transformation to *r*. The correlation coefficients were combined with unadjusted regression coefficients for the unadjusted data synthesis. The unadjusted and adjusted data (minimally adjusted for offspring's age and sex) were synthesized separately.

Furthermore, we tested whether there were differences between mother–offspring and father–offspring associations. Differences in SMD or MD were calculated for continuous outcomes. To explore the “fetal overnutrition” hypothesis, we compared the strength of associations restricted to studies that assessed parental BMI either before/during early pregnancy or if there were few studies, when the children were young.

Forest plots were constructed to display the individual and aggregate measures of association and their corresponding 95% CIs, in addition to tables presenting the aggregate results. We reported the *I* ^2^ statistic, which describes the percentage of the total variation across studies that is estimated to be due to between‐study heterogeneity.

#### 2.3.2 Secondary outcome (odds/risk of offspring being overweight or with obesity)

ORs and RRs and either their standard errors or 95% CI were directly extracted from study results. Because few studies reported obesity prevalence of more than 20% or reported RRs, ORs and RRs were summarized together.^28^ Studies varied in reporting parental or offspring weight status using different criteria, different reference groups (e.g., mother with normal weight, father with normal weight, both parents with normal weight), or varied in stratifying sex‐specific associations (e.g., mother/father–daughter, mother/father–son association, parent–daughter/parent–son association, or mother/father–offspring, or parent–offspring), precluding synthesizing results to a meaningful single summary OR. We therefore present forest plots showing individual study results without pooling ORs.^29^

### 2.4 Subgroup analyses

The following subgroup analyses were specified a priori for the primary analysis, where data were available: (i) BMI assessment method: measured versus self‐reported; (ii) study design: cross‐sectional study versus longitudinal study; (iii) maternal BMI measured before pregnancy versus after pregnancy; (iv) offspring age: early adulthood (younger than 30 years), mid‐adulthood (30–40 years), and later adulthood (older than 40 years).

### 2.5 Quality assessment

Quality assessment was done by two reviewers (J.Z. and C.C.D.) using a Newcastle–Ottawa scale (Appendix in the Supporting Information). The Newcastle–Ottawa scale evaluated the study design, representativeness of the exposed cohort, selection of the non‐exposed cohort, ascertainment of exposure, demonstration that outcome of interest was not present at start of study, comparability of cohorts on the basis of the design or analysis, assessment of offspring BMI, whether all adult participants in the analysis were the target age of the research question,^30^ and the adequacy of follow‐up of cohorts. We also considered how missing data were handled, whether studies included only biological parents and children (non‐paternity). We considered studies to have adjusted for confounding of the parental–offspring association if they had adjusted for the following: maternal age, family socioeconomic factors, smoking, drinking, and child sex. Studies were awarded one point per fulfilled criteria. We considered a total score ≥6 as good quality and a score of ≤3 as poor quality.

### 2.6 Post hoc analyses and publication bias

We conducted post hoc analyses by (i) parental and offspring BMI assessment time: similar young age (parental BMI measured before or during pregnancy‐offspring followed until early adulthood); late aged parent and young child (parental BMI measured at late age, when the child was school age, child BMI measured at young adulthood); parental mid/late age (both parental BMI and offspring BMI measured at mid or late adulthood); (ii) excluding seven studies^27^, ^31^, ^32^, ^33^, ^34^, ^35^, ^36^ (15% of studies and 5% of participants) that measured offspring BMI with some participants younger than 18 years (age range 15–25 years); or (iii) took out three studies^26^, ^32^, ^37^ with poor quality scores. To evaluate the potential effect of unpublished studies on our main findings (due to asymmetry or publication bias), we produced funnel plots and conducted Egger's regression.^38^

All analyses were conducted using the metan command in Stata BE version 17^39^ (StataCorp, College Station, TX, USA).

## 3 RESULTS

### 3.1 Descriptive statistics

The searches identified 32,689 studies, of which 90 met the abstract inclusion criteria. Of the studies that met the initial abstract screening, 44 were excluded from the review, because: study subjects were not adult offspring (*n* = 22), wrong exposure or outcome (*n* = 10), adiposity was not assessed by BMI (*n* = 3), exposure or outcome were BMI change (*n* = 4), articles were not in English (n = 1), wrong comparator (*n* = 1), wrong study design (*n* = 1), same cohort (*n* = 1), or conference abstract (*n* = 1). This resulted in 46 publications in the review (Figure S1). Of these studies, 26 were cohort studies, and 20 were cross‐sectional studies. Study populations were from Australia, Asia, Europe, and North and South America. Most of the studies included parent–offspring pairs and trios, with sample sizes ranging from 32 mother–daughter dyads^32^ to 36,528 father–mother–offspring trios.^40^ Two studies used a three‐generation cohort.^41^, ^42^ Parental and offspring BMIs were examined at different life stages across studies. Most cohort studies assessed parental BMI before/during early pregnancy and assessed child's BMI at early adulthood; nine cohort studies assessed parental BMI when children were young (mostly school age) and assessed the child's BMI at adulthood.^17^, ^25^, ^26^, ^43^, ^44^, ^45^, ^46^, ^47^ Cross‐sectional studies collected parental anthropometric information in parallel with that of the adult offspring. Study populations generally consisted of middle‐aged parents and young adult children^31^, ^32^, ^33^, ^34^, ^37^, ^48^, ^49^, ^50^, ^51^, ^52^, ^53^, ^54^ or both parents and children in middle or late adulthood.^18^, ^24^, ^40^, ^41^, ^42^, ^55^, ^56^ The information extracted from each study is presented in Table S1.

Various statistical methods were employed to analyze the relationship between parental BMI and adult offspring BMI. These included correlation coefficient methods, linear regression models, logistic or multinomial models, and multilevel models.

Most linear regression studies adjusted for family socioeconomic factors, maternal age, smoking, drinking, and other important confounders (Figure S2 depicting our directed acyclic diagram). The majority of studies found positive associations between parents and their adult offspring BMI, while four studies reported null associations.^26^, ^37^, ^41^, ^53^ Several studies investigated sex‐specific associations and compared the same‐sex (mother–daughter, or father–son) or cross‐sex (mother–son, father–daughter) relationships within the same population.^18^, ^24^, ^25^, ^54^, ^57^ Conclusions regarding the findings for these sex‐specific results varied between the studies. Detailed information is found in Tables S2–S4.

### 3.2 SMD for continuous measures of BMI

Associations of continuous measures of maternal and/or paternal BMI with offspring BMI were reported in 35 studies. These studies used different BMI units and not all adjusted for potential confounders. We therefore pooled results separately for (i) confounder‐adjusted SMD (*n* = 15 studies), (ii) unadjusted (for any covariates) SMD (*n* = 21), and (iii) confounder‐adjusted associations with parental and offspring BMI in 1 kg/m^2^ units (*n* = 9).

**FIGURE 1:**
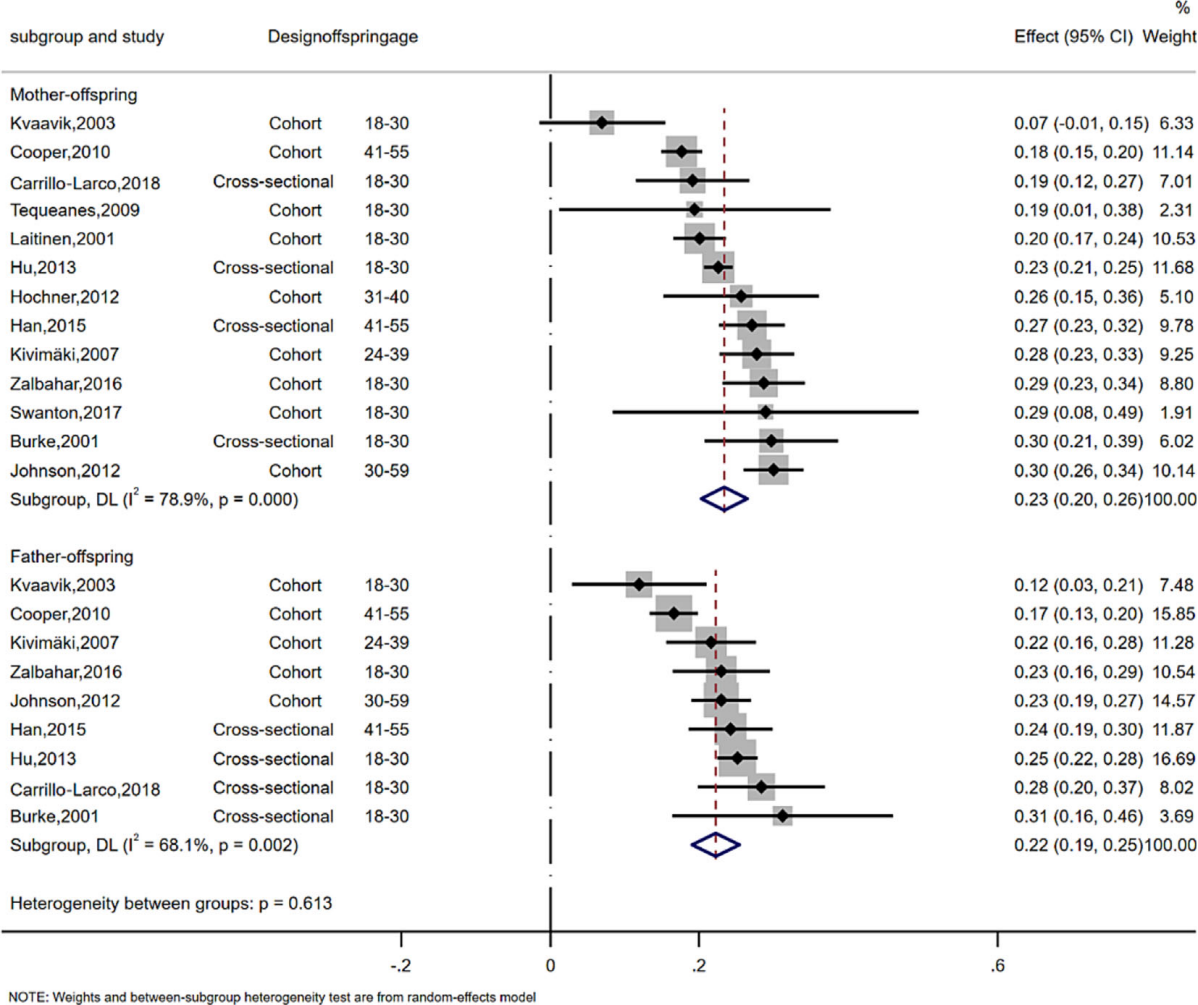
Meta‐analysis of the association between parent and offspring BMI (SMD). The effect size estimate is the SMD with 95% CI (per SD of parental BMI). The size of the squares' estimates is proportional to the weight assigned to each study. Diamonds represent pooled estimates from a random effects meta‐analysis. The *I* ^2^ and *P* values for heterogeneity are shown. BMI, body mass index; CI, confidence interval; SD, standard deviation; SMD, standardized mean difference.

**FIGURE 2:**
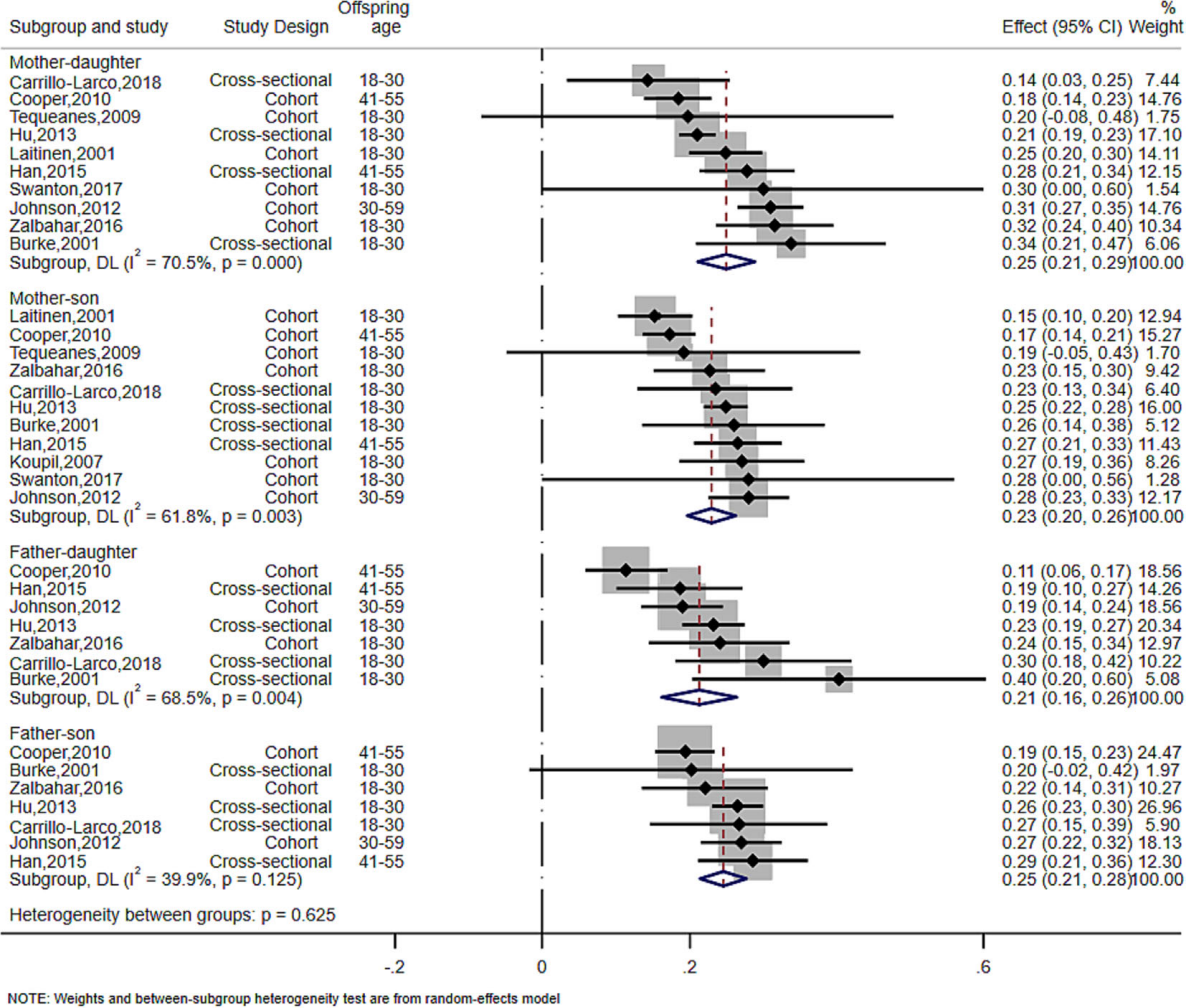
Meta‐analysis of the association between parent and offspring BMI at sex‐specific level (SMD). The effect size estimate is the SMD with 95% CI (per SD of parental BMI). The size of the squares' estimates is proportional to the weight assigned to each study. Diamonds represent pooled estimates from a random effects meta‐analysis. The *I* ^2^ and *P* values for heterogeneity are shown. BMI, body mass index; CI, confidence interval; SD, standard deviation; SMD, standardized mean difference.

**FIGURE 3:**
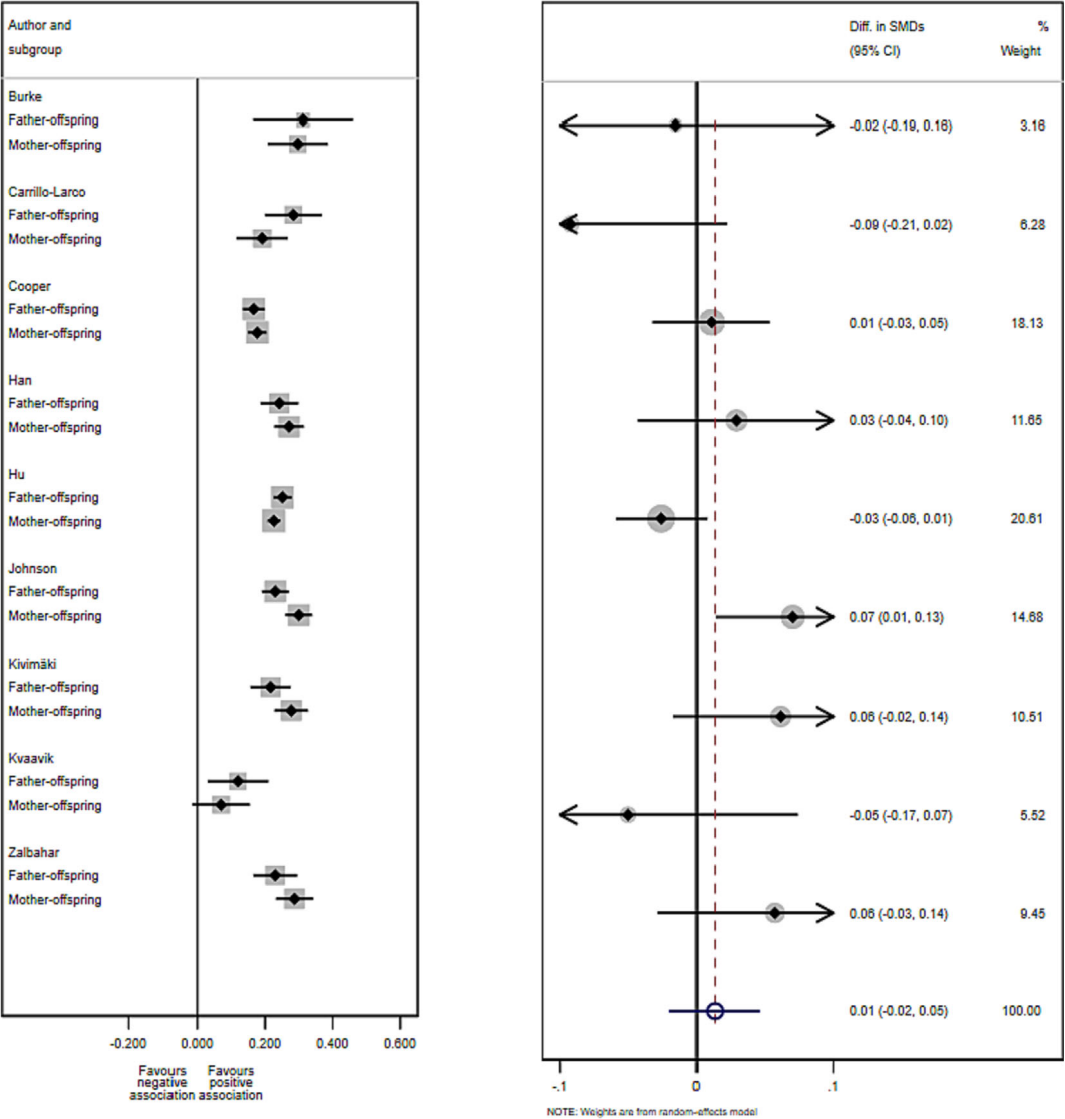
Difference in SMD between mother–offspring and father–offspring BMI associations. Left plot: SMD (95% CI) in each subgroup of each study. Right plot: Difference of SMD comparing effects for mother–offspring and father–offspring in each study with a fixed‐effects meta‐analysis of these differences of SMD. The size of the squares and circles is proportional to the weight assigned to each study. Open circles represent pooled estimates. CI, confidence interval; SMD, standardized mean difference.

Across all of these studies, results were consistent with similar positive associations for mother–offspring and father–offspring. For example, the pooled analyses showed that the mother–offspring SMD was 0.23 (95% CI: 0.20, 0.26 per SD greater maternal BMI), and the father–offspring was 0.22 (95% CI: 0.19, 0.25 per SD greater paternal BMI) in adjusted models (Figure 1). The equivalent unadjusted SMD results were 0.24 (95% CI: 0.21, 0.28) for mother–offspring and 0.21(95% CI: 0.18, 0.25) for father–offspring (Figure S3A, with additional result in Table S5). There was evidence of between study heterogeneity in both analyses (*I* ^2^% = 79% for mother–offspring, *I* ^2^% = 68% for father–offspring). The synthesized sex‐specific adjusted and unadjusted SMDs revealed similar positive associations for mother–daughter, mother–son, father–daughter, and father–son (Figure 2, Figure 3B, Table S5).

Pooled results from 15 studies of the mean difference in offspring BMI (kg/m^2^) per 1 kg/m^2^ parental BMI were consistent with those for the SMD. There were also high levels of between study heterogeneity (Figure S4A, Figure S4B, and Table S6).

We then compared the strength of associations between maternal and paternal BMI and offspring BMI by calculating the difference in SMD. There was no strong evidence for differences by offspring gender for either maternal or paternal associations (difference in SMD = 0.01, 95% CI: −0.02, 0.05; Figure 3). Similar approaches were applied to unadjusted SMD and MD, and the results remained similar (Figure S5, Figure S6, and Tables S7–S9).

There was no evidence of a difference between maternal and paternal BMI–offspring BMI associations when restricting to studies assessing parental BMI when the children were young (difference in SMD = 0.02, 95% CI: −0.01, 0.05; Figure S7, Table S10).

There were six studies that could not be included in the meta‐analyses.^36^, ^40^, ^55^, ^58^, ^59^, ^60^ The reported findings were consistent with the meta‐analyzed studies. For detailed descriptions, see Table S11.

### 3.3 Subgroup and post hoc analyses

There was evidence that associations were weaker when parental BMI was based on parental self‐reported weight and height rather than on clinical or research measures (e.g., self‐reported SMD = 0.21 (95% CI: 0.15, 0.27, *p* < 0.001) for mother–offspring, and clinical or research measures SMD = 0.26 (95% CI: 0.23, 0.30, *p* = 0.006), respectively; exact *p* < 0.1 for heterogeneity between groups; Figure S8, Table S12). The association was stronger in cross‐sectional studies compared to cohort studies for father–offspring associations but not for mother–offspring associations (Figure S9A, Table S13).

Pooled results were very similar comparing studies where maternal BMI ascertainment was before/in early pregnancy or after pregnancy (Figure S10, Table S14); similarly, results were consistent between studies in which children were younger than 30 years and those who were older than 40 years (Figure S11A, Figure S11B, and Table S15). When considering temporal relationships in the assessment of BMI for both parents and offspring, the strengths of the parental–offspring BMI associations were similar when offspring BMI was measured in early adulthood irrespective of timing of parental BMI measurement. Results were more heterogeneous when both parental and offspring BMI were measured at mid/late adulthood, mostly due to the variety in offspring's BMI assessment time and study design (Figure S12A,B, Table S16).

Further post hoc analyses were performed to investigate the moderate‐to‐high degree of between‐study heterogeneity, including taking out studies with a small proportion of offspring younger than 18 years (Table S17) or with low quality (Table S18). The results remained similar to the main analyses.

### 3.4 Secondary outcome: ORs

Eighteen studies investigated the association between parental BMI status and offspring having overweight or obesity and were included. Eleven of the studies^18^, ^26^, ^34^, ^36^, ^43^, ^46^, ^47^, ^49^, ^50^, ^51^, ^56^, ^57^, ^60^, ^61^, ^62^, ^63^, ^64^, ^65^, ^66^, ^67^ used World Health Organization^68^ categories to define overweight (BMI 25.0–29.9 kg/m^2^) and obesity (BMI ≥ 30 kg/m^2^); the remaining studies used different thresholds based on the study population distribution.

As noted in Section 2, we did not pool results from these studies. Most studies found that adult offspring of parents with overweight or obesity (either mother or father, or both) were more likely to be with overweight or obesity themselves, compared to those with parents with normal weight, though magnitudes of association varied ranging from 1.1 (95% CI: 0.8, 1.6)^46^ to 11.8 (95% CI: 7.7, 18.0)^18^ (see Figures S13–S15).

Only one study found little evidence of an association between mother–offspring or father–offspring weight status.^50^ ORs were generally higher when both parents had overweight or obesity compared to results using where only one parent had overweight or obesity, and there was evidence of a dose response with stronger magnitudes of association for those whose parents had obesity rather than overweight. Four studies examined the association of parental BMI as a continuum with ORs for offspring having overweight or obesity, with results for these showing higher odds of offspring having overweight or obesity with higher parental mean BMI.^34^, ^62^, ^66^, ^67^

### 3.5 Study quality and risk of bias

The majority of the studies were considered of good quality (29 scored ≥6), with four studies scoring 8 and three studies scoring ≤3 (Table S19). Eleven studies did not have a representative sample of the target population, and seven studies did not consider the temporal relationship between the exposure and outcome. The exposure or outcome was self‐reported or without description in 15 studies, and confounding factors were not considered in the analyses in 10 studies. Thirteen cross‐sectional studies did not score a point for adequacy of follow up of the cohort due to the nature of the study design; 17 cohort studies had a follow‐up of <80% and did not provide any description of loss to follow‐up. All studies were followed up long enough for outcomes to occur (average offspring age was above 18 years old). Sensitivity analyses taking out the three studies of poor quality (score ≤3) showed slightly higher SMD and lower *I* ^**2**^ in the adjusted models (data not shown).

### 3.6 Funnel plot asymmetry

The funnel plot and Egger's test show no strong evidence of funnel asymmetry suggesting there was little evidence of publication bias (Figure S16).

## 4 DISCUSSION

In this systematic review, we found positive associations between parental BMI and their offspring BMI in adulthood, assessed using several different measures of association. Directions and magnitudes of associations were similar for both parents and when assessed separately in daughters and sons. They were also similar in subgroups of studies that differed in age at which offspring weight and height were ascertained and in timing of maternal BMI in relation to pregnancy. Most studies were considered good quality, with only few scoring poorly, and we observed that results in studies with BMI (parental and/or offspring) based on self‐reported rather than measured weight and height were weaker. The findings extend previous systematic reviews on the intergenerational transmission of BMI in childhood^6^, ^19^ and demonstrate that the association persists into adulthood. The strength of association across the BMI distribution was relatively weak. For example, the pooled SMD = 0.23 per 1 SD higher maternal BMI is equivalent to a correlation coefficient of 0.23. On the other hand, this correlation could be important at a population level.

While our meta‐analyses were focused on investigating the parent–offspring BMI association in adulthood in general, this relationship might be influenced by a range of factors that vary across different stages of the life course. Such dynamic factors might influence the strength of association in different periods. Nevertheless, subgroup analyses demonstrate that the associations were consistent when considering temporal relationships in the assessment time of BMI for both parents and offspring. Notably, only birth cohort studies were able to prospectively assess parental BMI before/during pregnancy, whereas some of the meta‐analyzed studies measured parental BMI concurrently with offspring BMI. Consequently, it is conceivable that any intrauterine effects might be diluted with increasing age. However, our results were similar for studies that measured parental BMI before the index pregnancy. That we see similar magnitudes of associations for mothers and fathers is consistent with previous parental negative control and Mendelian randomization studies,^15^, ^16^, ^69^, ^70^, ^71^, ^72^, ^73^ which suggest that intrauterine mechanisms are not key to cross‐generational associations. The comparison of same‐sex versus cross‐sex association also did not reveal significant sex differences in the associations. Our findings are consistent with both parents contributing to an “obesogenic” family environment, but are also compatible with the inheritance from either parent, or both, of genetic traits predisposing to adiposity.^74^ Thus, these findings emphasize the need to target all family members for obesity prevention.

Between‐study heterogeneity was moderate to high for some of the pooled results. We conducted a series of subgroup analyses to explore the sources of heterogeneity but found consistent results between subgroups for all of these, except whether BMI was based on self‐reported or measured weight and height. Results were consistent between studies that did not adjust for any confounders and those adjusting for some confounders (most of which adjusted for what we consider to be key confounders). Thus, differences in confounder adjustments are unlikely to explain between‐study heterogeneity, and we are unable to determine the cause of between‐study heterogeneity. Six studies adjusted for early life variables such as birth weight or gestational weight gain,^53^, ^59^, ^63^, ^65^, ^75^, ^76^ which are potential mediators on the causal pathway between parental and offspring BMI, rather than confounders. Therefore, we used results from models that did not adjust for birth weight or gestational weight gain in the meta‐analyses.

Key strengths of our review include the focus on offspring adult BMI, carefully considering which studies are appropriate to meta‐analyses, and undertaking relevant subgroup analyses. We have investigated sex‐specific associations and compared associations of maternal–offspring to paternal–offspring BMI associations, including the timing of BMI measurement for both parents and children, to explore the potential etiology of BMI intergenerational associations.

We acknowledge limitations of this review. Of the 46 studies identified in the systematic review, some studies could not be included in the meta‐analyses.^36^, ^40^, ^55^, ^58^, ^59^, ^60^ The description of results and conclusions of those studies were largely consistent with the findings of the meta‐analyses in that they suggested parental BMI associated with offspring BMI into adulthood. While our focus was on offspring adult (18 years or older) BMI, seven studies^27^, ^31^, ^32^, ^33^, ^34^, ^35^, ^36^ (corresponding to 15% of studies and 5% of participants) measuring offspring BMI at 15–25 years were included in the systematic review. However, meta‐analysis results were not changed with removal of these studies. We have focused on adult BMI, and it is possible that results would be different for other measures of adiposity, such as waist circumference or fat mass. However, the strong correlations between BMI and these measurements, together with similar Mendelian randomization results for maternal BMI with offspring BMI and fat mass, make this unlikely.^77^ The small number of studies reporting all combinations of mother, father, daughter, and son associations limited our ability to assess any dose–response relationships between within these different groups.

## 5 CONCLUSIONS

This systematic review and meta‐analysis of observational studies indicates that the intergenerational associations between parental and offspring BMI persist into adulthood. We found there was no strong evidence of differences between maternal and paternal lines, which, together with Mendelian randomization and negative control studies^6^, ^16^ mostly in younger aged offspring, suggest that intrauterine effects related to higher maternal BMI are not a major cause of adiposity in offspring.

## AUTHOR CONTRIBUTIONS

Jie Zhang and Christina C. Dahm had the original idea for the paper and designed the study. Jie Zhang, Gemma L. Clayton, Kim Overvad, Anja Olsen, Deborah A. Lawlor, and Christina C. Dahm contributed to the analysis plan. The bibliographic search and data extraction was carried out by Jie Zhang, Helene Tilma Vistisen, Mette Lise Lousdal, and Christina C. Dahm. The methods and statistical analysis were performed by Jie Zhang and Gemma L. Clayton. The interpretation of results and writing and final editing was done by all authors. All authors reviewed and approved the manuscript.

## CONFLICT OF INTEREST STATEMENT

DAL has received support from Roche Diagnostics for research unrelated to this paper; other authors declare no conflicts of interest.

## Supporting information


**Figure S1.** PRISMA flow diagram of the study selection process
**Figure S2.** Directed acyclic diagram (DAG) illustrating confounding factors between parent‐offspring BMI association.
**Figure S3a.** Meta‐analysis of the association between parent and offspring BMI (standardized mean difference).
**Figure S3b.** Meta‐analysis of the association between parent and offspring BMI at sex‐specific level (standardized mean difference).
**Figure S4a.** Meta‐analysis of the association between parent and offspring BMI (mean difference).
**Figure S4b.** Meta‐analysis of the association between parent and offspring BMI at sex‐specific level (mean difference).
**Figure S5.** Difference of standardized mean difference between mother‐offspring and father‐offspring association.
**Figure S6.** Difference of mean difference between mother‐offspring and father‐offspring association.
**Figure S7.** Difference of standardized mean difference between mother‐offspring and father‐offspring association in studies assessing parental BMI at early age.
**Figure S8.** Standardized mean difference between parent‐offspring BMI association‐subgroup analyses by BMI measurement methods.
**Figure S9a.** Standardized mean difference between parent‐offspring BMI association‐subgroup analyses by study design.
**Figure S9b.** Standardized mean difference between parent‐offspring BMI association‐subgroup analyses by study design (sex‐specific level).
**Figure S10.** Standardized mean difference between parent‐offspring BMI association‐subgroup analyses by maternal BMI measurement time.
**Figure S11a.** Standardized mean difference between parent‐offspring BMI association‐subgroup analyses by offspring age.
**Figure S11b.** Standardized mean difference between parent‐offspring BMI association‐subgroup analyses by offspring age (sex‐specific level).
**Figure 12a.** Standardized mean difference between parent‐offspring BMI association‐subgroup analyses by parent‐offspring BMI assessed time.
**Figure 12b.** Standardized mean difference between parent‐offspring BMI association‐subgroup analyses by parent‐offspring BMI assessed time (sex‐specific level).
**Figure S13.** Forest plot showing odds ratio (OR) of offspring with overweight with parental weight status.
**Figure S14.** Forest plot showing odds ratio (OR) of offspring with obesity with parental weight status.
**Figure S15.** Forest plot showing odds ratio (OR) of offspring with overweight or obesity with parental weight status.
**Figure S16.** Funnel plot for publication bias.
**Table S1.** Description of all the studies included in systematic review and meta‐analyses.
**Table S2.** Descriptions of studies reporting correlation coefficient.
**Table S3.** Descriptions of studies reporting mean difference or standardized mean difference.
**Table S4.** Descriptions of studies reporting odd ratios or risk ratios.
**Table S5.** Pooled standardized mean difference between parental and offspring BMI (per standard deviation).
**Table S6.** Pooled mean difference between parental and offspring BMI (per kg/m2).
**Table S7.** Difference of standardized mean difference between maternal and paternal line in adjusted models.
**Table S8.** Difference of standardized mean difference between maternal and paternal line in unadjusted models.
**Table S9.** Difference of mean difference between maternal and paternal line.
Table S10. Difference of mean difference between maternal and paternal line (restricting to studies assessing parental BMI when children were young).
**Table S11.** Summary of studies not included in meta‐analyses.
**Table S12.** Subgroup analyses by BMI measurement method.
**Table S13.** Subgroup analyses by study design.
**Table S14.** Subgroup analyses by maternal BMI measurement time.
**Table S15.** Subgroup analyses by offspring age.
**Table S16.** Subgroup analyses by parent‐offspring BMI assessed time.
**Table S17.** Post hoc analyses‐standardized mean difference taking out studies including younger participants.
**Table S18.** Post hoc analyses‐standardized mean difference taking out studies with low quality score.
**Table S19.** Study quality assessment using Adapted Newcastle‐Ottawa scale.
