# Supplementary material for: Body mass index in parents and their adult offspring: A systematic review and meta‐analysis
Source: Obes Rev. 2023 Oct 2;25(1):e13644. doi: 10.1111/obr.13644 (PMC10909538; doi:10.1111/obr.13644)
Supplement: Supplementary file 1 — Figure S1. PRISMA flow diagram of the study selection process Figure S2. Directed acyclic diagram (DAG) illustrating confounding factors between parent‐offspring BMI association. Figure S3a. Meta‐analysis of the association between parent and offspring BMI (standardized mean difference). Figure S3b. Meta‐analysis of the association between parent and offspring BMI at sex‐specific level (standardized mean difference). Figure S4a. Meta‐analysis of the association between parent and offspring BMI (mean difference). Figure S4b. Meta‐analysis of the association between parent and offspring BMI at sex‐specific level (mean difference). Figure S5. Difference of standardized mean difference between mother‐offspring and father‐offspring association. Figure S6. Difference of mean difference between mother‐offspring and father‐offspring association. Figure S7. Difference of standardized mean difference between mother‐offspring and father‐offspring association in studies assessing parental BMI at early age. Figure S8. Standardized mean difference between parent‐offspring BMI association‐subgroup analyses by BMI measurement methods. Figure S9a. Standardized mean difference between parent‐offspring BMI association‐subgroup analyses by study design. Figure S9b. Standardized mean difference between parent‐offspring BMI association‐subgroup analyses by study design (sex‐specific level). Figure S10. Standardized mean difference between parent‐offspring BMI association‐subgroup analyses by maternal BMI measurement time. Figure S11a. Standardized mean difference between parent‐offspring BMI association‐subgroup analyses by offspring age. Figure S11b. Standardized mean difference between parent‐offspring BMI association‐subgroup analyses by offspring age (sex‐specific level). Figure 12a. Standardized mean difference between parent‐offspring BMI association‐subgroup analyses by parent‐offspring BMI assessed time. Figure 12b. Standardized mean difference between parent‐offspring BMI associ [file OBR-25-e13644-s001.pdf]

BODY MASS INDEX IN PARENTS AND THEIR ADULT OFFSPRING: A SYSTEMATIC REVIEW AND META-ANALYSIS

Supplementary information

Jie Zhang[1], Gemma L Clayton[2,3], Kim Overvad[1], Anja Olsen[1,4], Deborah A Lawlor[2,3], Christina C Dahm[1]

[1] Department of Public Health, Aarhus University, Aarhus, Denmark

[2] Population Health Sciences, Bristol Medical School, University of Bristol, Bristol, United Kingdom

[3] MRC Integrative Epidemiology Unit at the University of Bristol, Bristol, United Kingdom

[4] Danish Cancer Society Research Center, Copenhagen, Denmark

Correspondence: Christina C. Dahm (ccd@ph.au.dk)

Department of Public Health, Aarhus University, Bartholins Allé 2, DK-8000 Aarhus C, Denmark

Contents

Figure S1. PRISMA flow diagram of the study selection process

Figure S2. Directed acyclic diagram (DAG) illustrating confounding factors between parent-offspring BMI association

Figure S3a. Meta-analysis of the association between parent and offspring BMI (standardized mean difference)

Figure S3b. Meta-analysis of the association between parent and offspring BMI at sex-specific level (standardized mean difference)

Figure S4a. Meta-analysis of the association between parent and offspring BMI (mean difference)

Figure S4b. Meta-analysis of the association between parent and offspring BMI at sex-specific level (mean difference)

Figure S5. Difference of standardized mean difference between mother-offspring and father-offspring association

Figure S6. Difference of mean difference between mother-offspring and father-offspring association

Figure S7. Difference of standardized mean difference between mother-offspring and father-offspring association in studies assessing parental BMI at early age

Figure S8. Standardized mean difference between parent-offspring BMI association-subgroup analyses by BMI measurement methods

Figure S9a. Standardized mean difference between parent-offspring BMI association-subgroup analyses by study design

Figure S9b. Standardized mean difference between parent-offspring BMI association-subgroup analyses by study design (sex-specific level)

Figure S10. Standardized mean difference between parent-offspring BMI association-subgroup analyses by maternal BMI measurement time

Figure S11a. Standardized mean difference between parent-offspring BMI association-subgroup analyses by offspring age

Figure S11b. Standardized mean difference between parent-offspring BMI association-subgroup analyses by offspring age (sex-specific level)

Figure 12a. Standardized mean difference between parent-offspring BMI association-subgroup analyses by parent-offspring BMI assessed time

Figure 12b. Standardized mean difference between parent-offspring BMI association-subgroup analyses by parent-offspring BMI assessed time (sex-specific level)

Figure S13. Forest plot showing odds ratio (OR) of offspring with overweight with parental weight status

Figure S14. Forest plot showing odds ratio (OR) of offspring with obesity with parental weight status

Figure S15. Forest plot showing odds ratio (OR) of offspring with overweight or obesity with parental weight status

Figure S16. Funnel plot for publication bias

Table S1. Description of all the studies included in systematic review and meta-analyses

Table S2. Descriptions of studies reporting correlation coefficient

Table S3. Descriptions of studies reporting mean difference or standardized mean difference

Table S4. Descriptions of studies reporting odd ratios or risk ratios

Table S5. Pooled standardized mean difference between parental and offspring BMI (per standard deviation)

Table S6. Pooled mean difference between parental and offspring BMI (per kg/m^2)

Table S7. Difference of standardized mean difference between maternal and paternal line in adjusted models

Table S8. Difference of standardized mean difference between maternal and paternal line in unadjusted models

Table S9. Difference of mean difference between maternal and paternal line

Table S10. Difference of mean difference between maternal and paternal line (restricting to studies assessing parental BMI when children were young)

Table S11. Summary of studies not included in meta-analyses

Table S12. Subgroup analyses by BMI measurement method

Table S13. Subgroup analyses by study design

Table S14. Subgroup analyses by maternal BMI measurement time

Table S15. Subgroup analyses by offspring age

Table S16. Subgroup analyses by parent-offspring BMI assessed time

Table S17. Post hoc analyses-standardized mean difference taking out studies including younger participants

Table S18. Post hoc analyses-standardized mean difference taking out studies with low quality score

Table S19. Study quality assessment using Adapted Newcastle-Ottawa scale

Appendix

Figure S1. PRISMA flow diagram of the study selection process

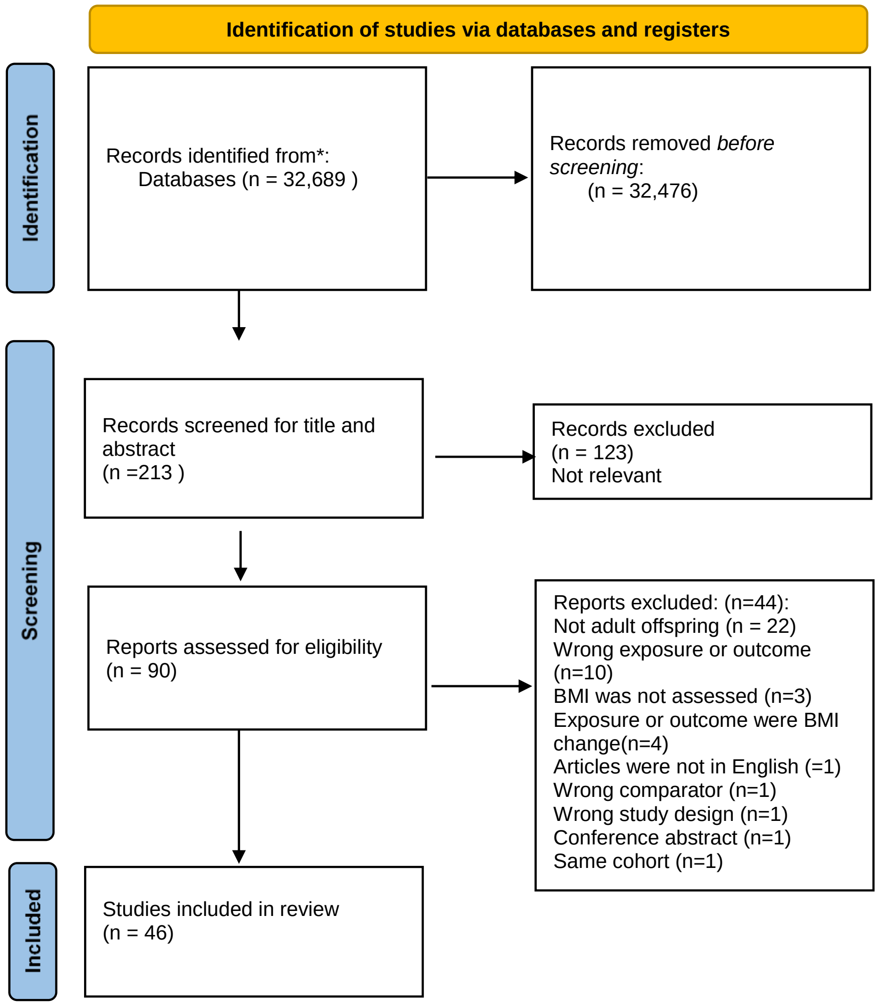

Figure S2. Directed acyclic diagram (DAG) illustrating confounding factors between parent-offspring BMI association

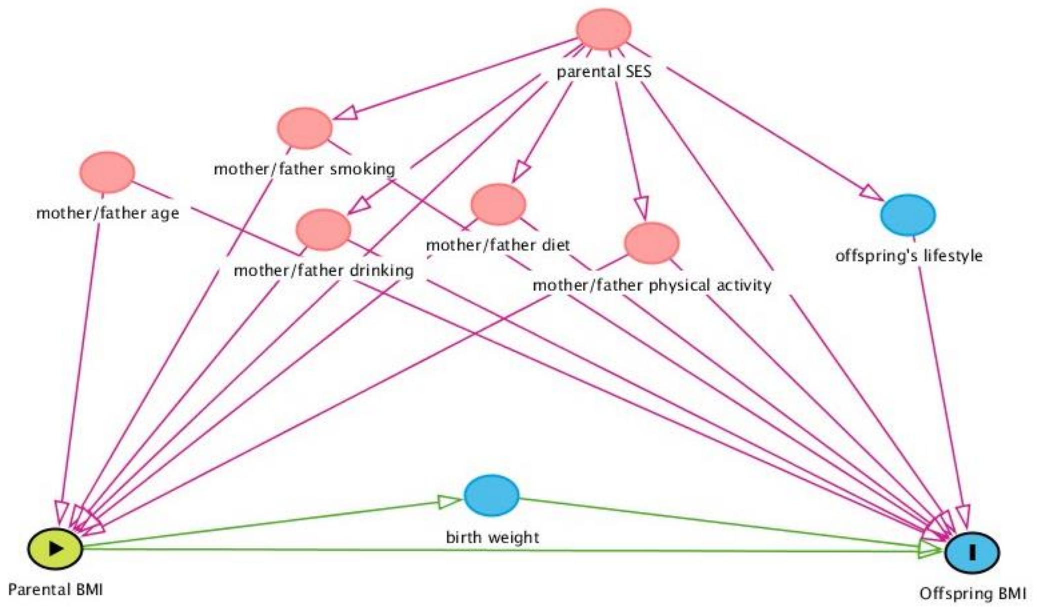

*****Minimal adjusted confounders: parental age, parental socioeconomic status (SES), smoking, drinking, physical activity or diet.

Figure S3a. Meta-analysis of the association between parent and offspring BMI (standardized mean difference)*

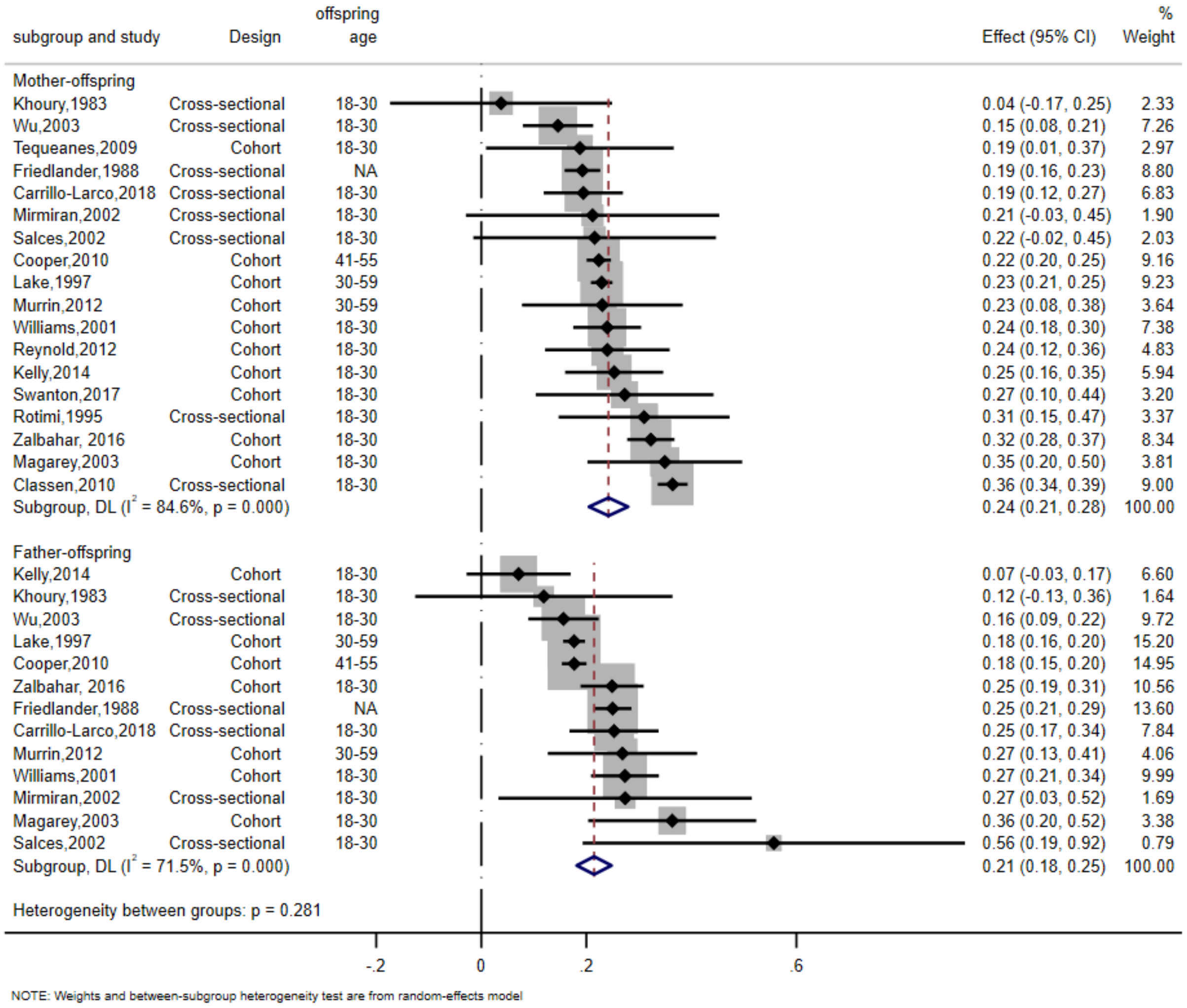

*pooled SMD for unadjusted models.

Figure S3b. Meta-analysis of the association between parent and offspring BMI at sex-specific level (standardized mean difference)*

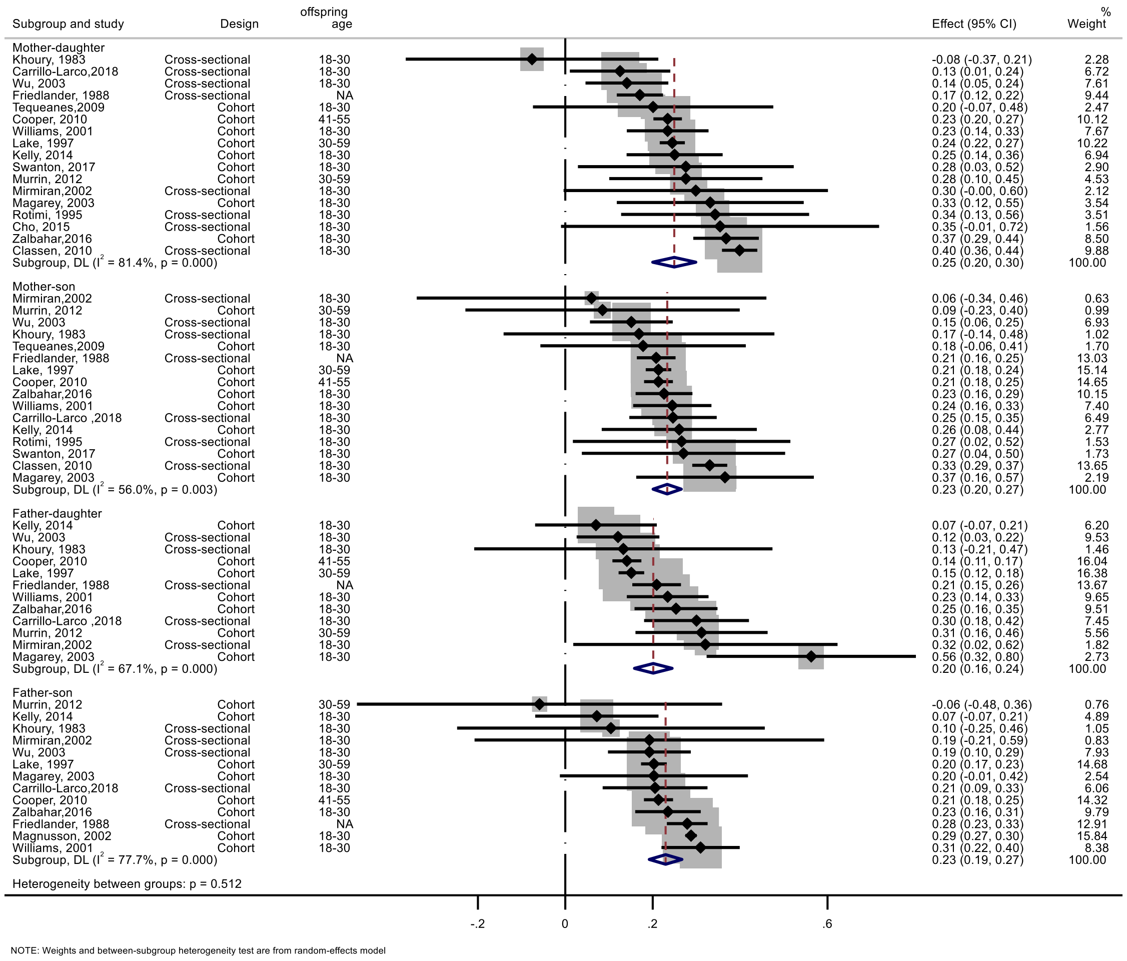

*pooled SMD for unadjusted models

Figure S4a. Meta-analysis of the association between parent and offspring BMI (mean difference)

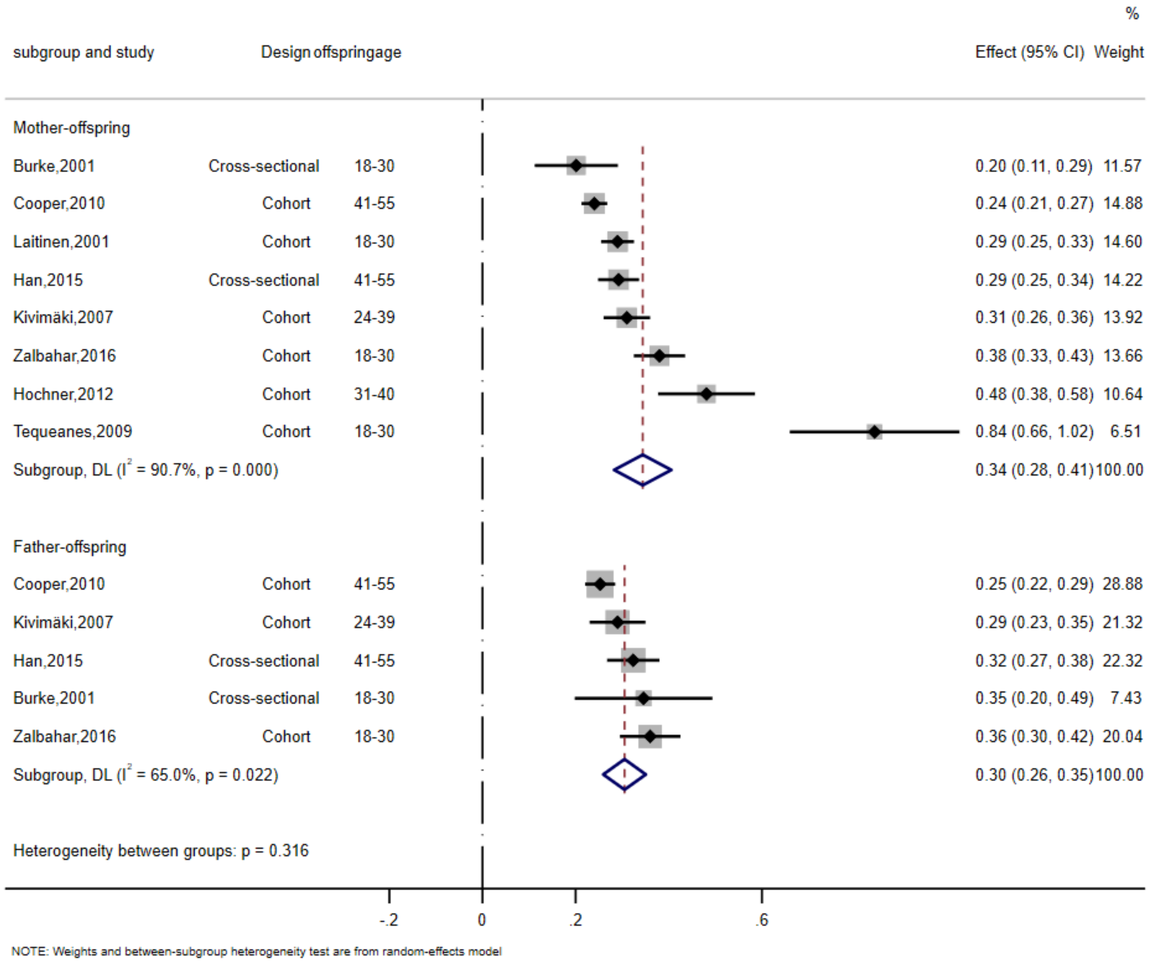

Figure S4b. Meta-analysis of the association between parent and offspring BMI at sex-specific level (mean difference)

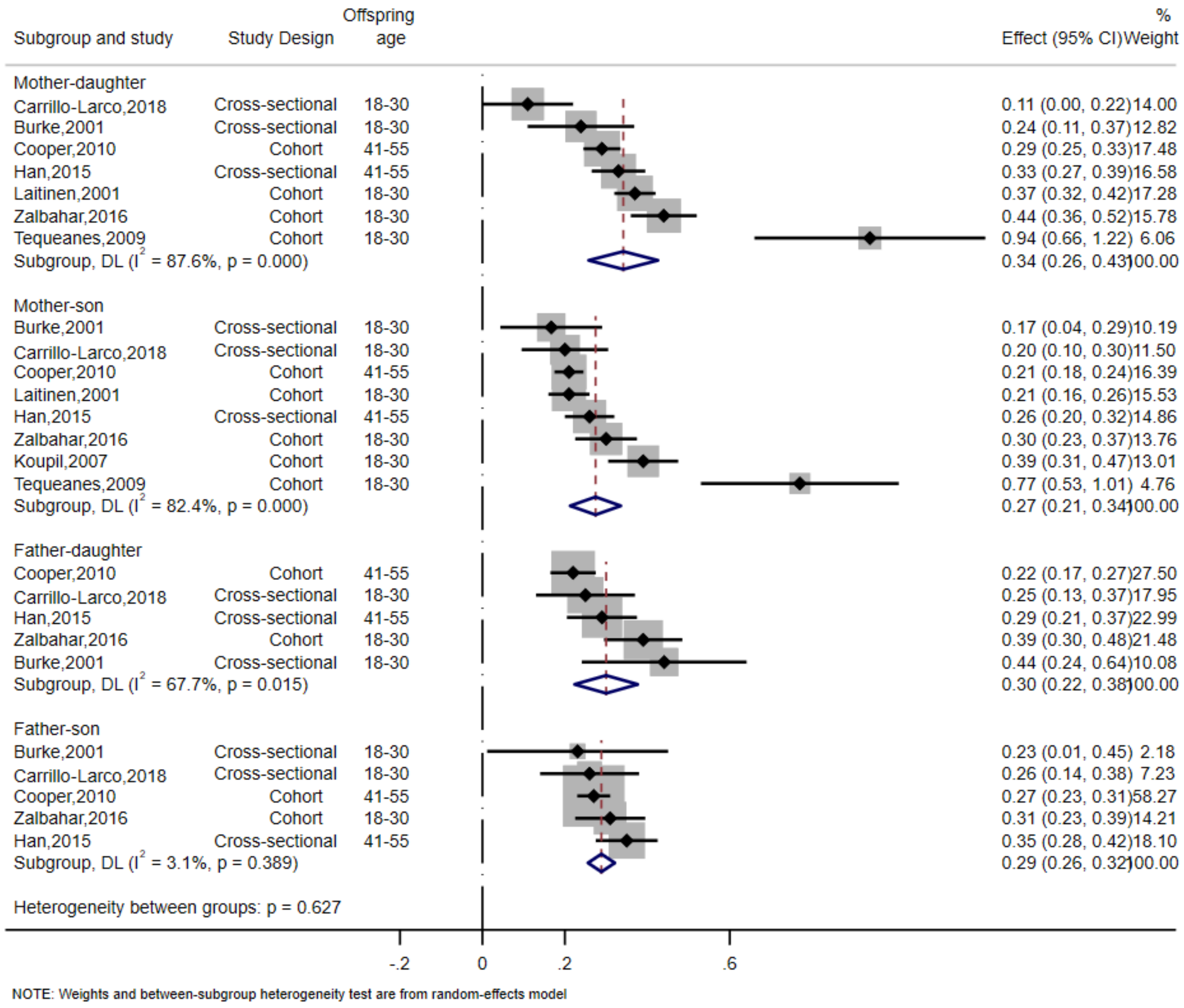

Figure S5. Difference of standardized mean difference between mother-offspring and father-offspring association*

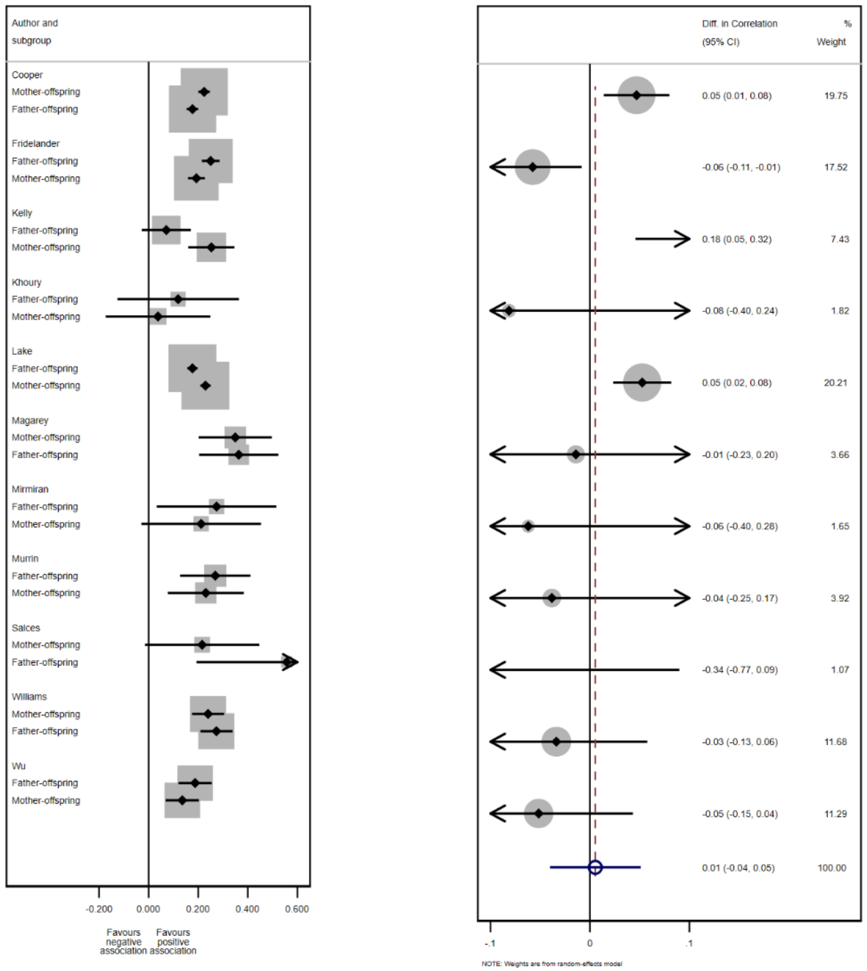

*unadjusted models

Figure S6. Difference of mean difference between mother-offspring and father-offspring association

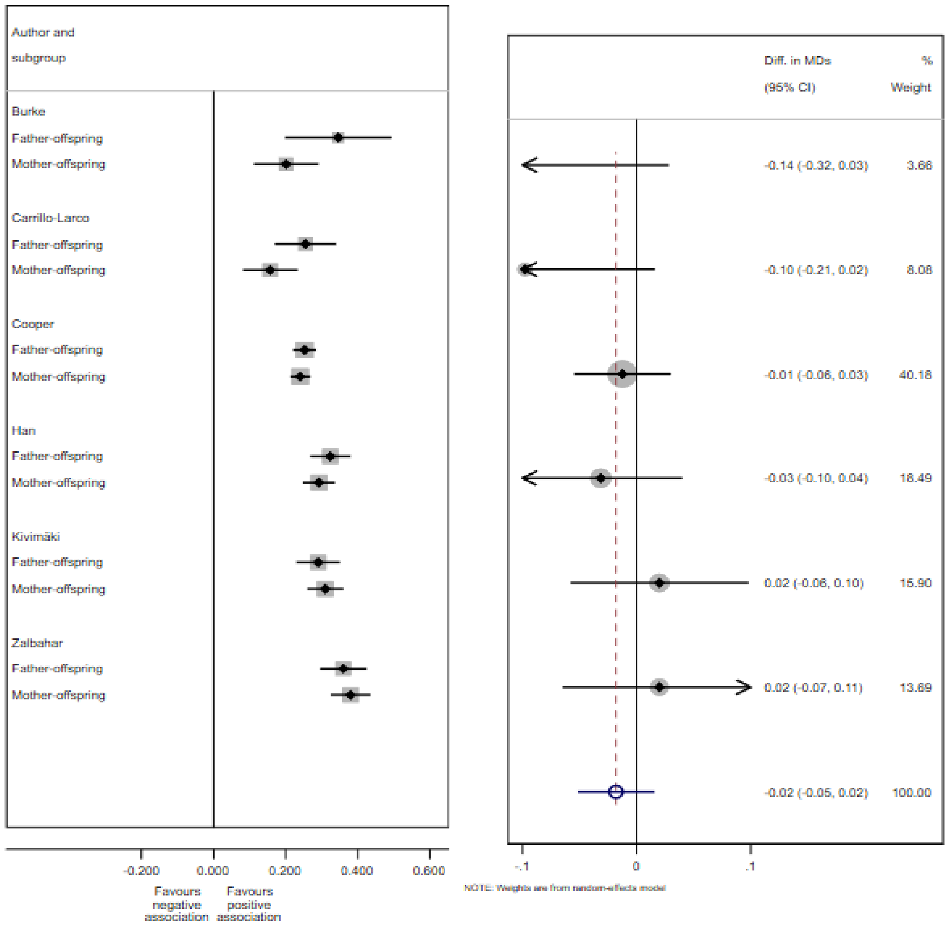

Figure S7. Difference of standardized mean difference between mother-offspring and father-offspring association in studies assessing parental BMI at early age

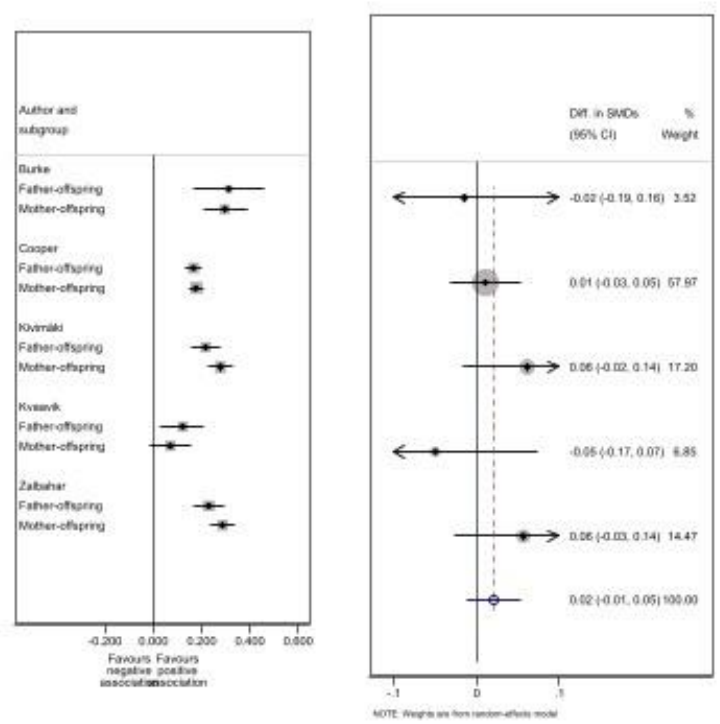

*restricting to studies assessed parental BMI at early stage

Figure S8. Standardized mean difference between parent-offspring BMI association-subgroup analyses by BMI measurement methods

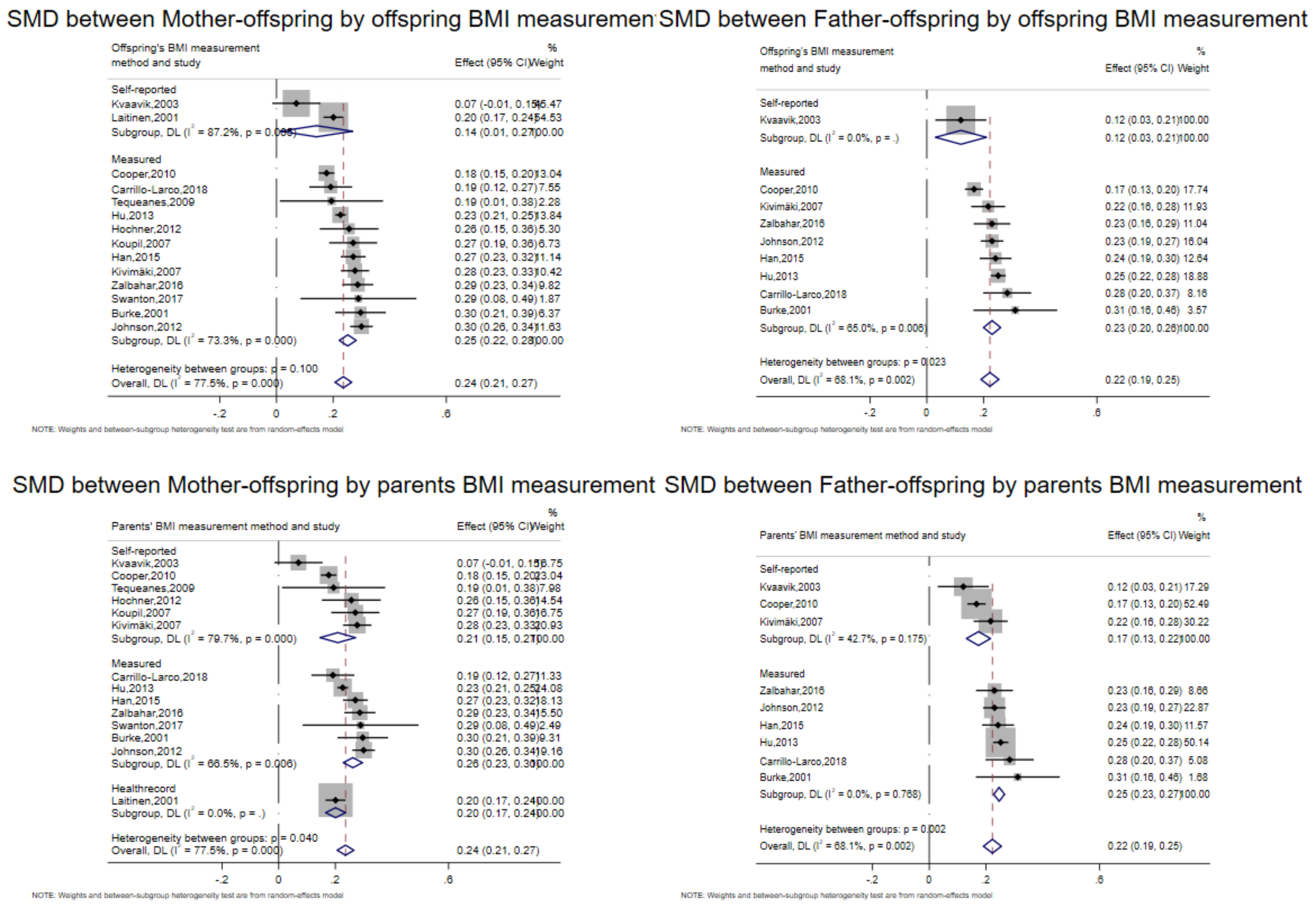

SMD-Standardized mean difference

Figure S9a. Standardized mean difference between parent-offspring BMI association-subgroup analyses by study design

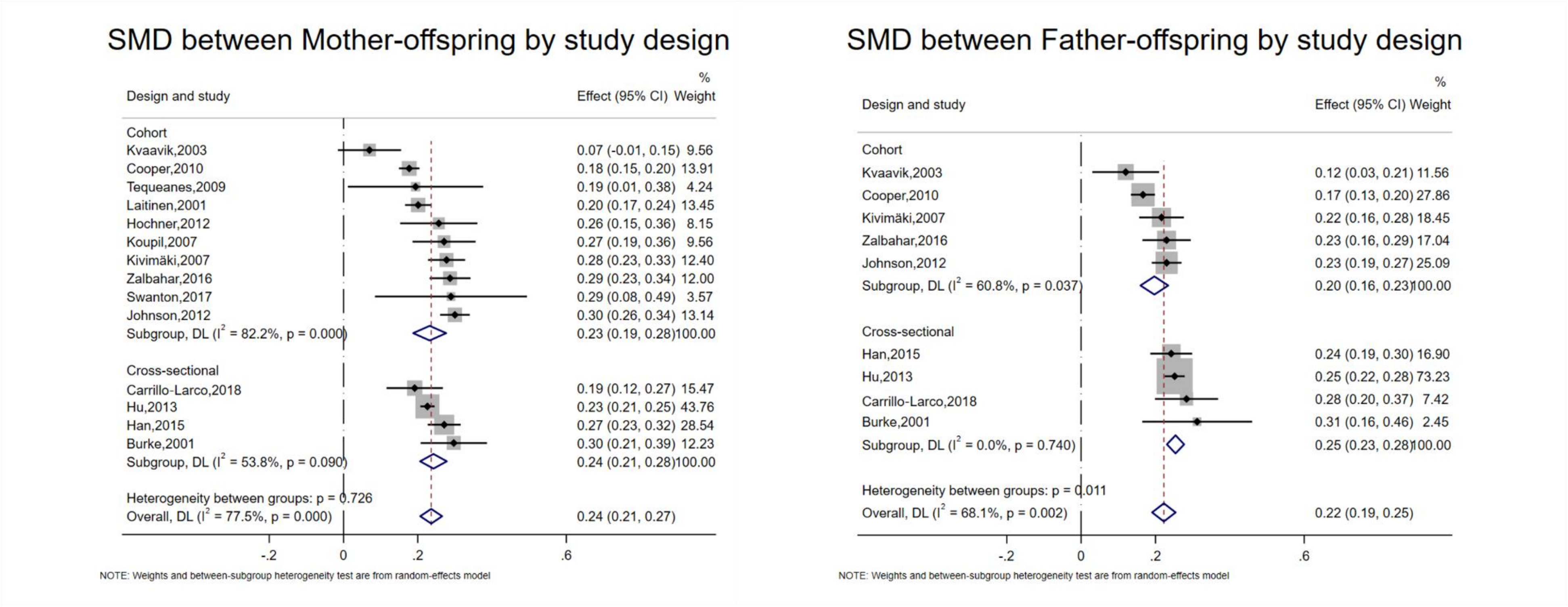

SMD-standardized mean difference

Figure S9b. Standardized mean difference between parent-offspring BMI association-subgroup analyses by study design (sex-specific level)

SMD between Mother-daughter by study design

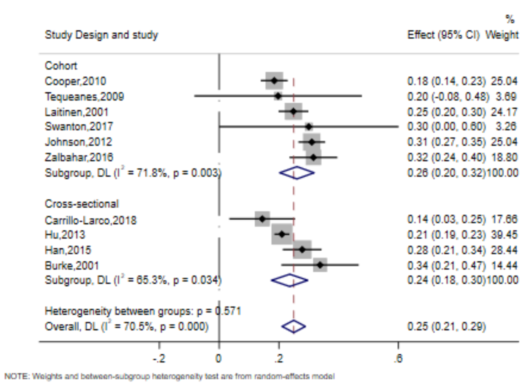

SMD between Mother-son by study design

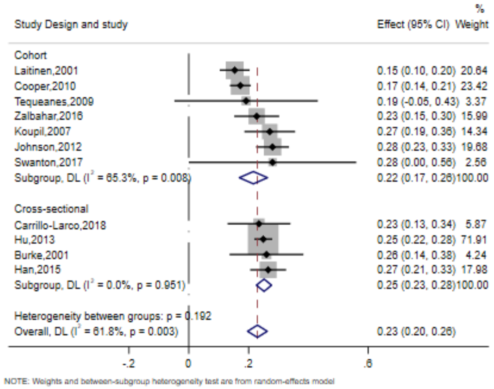

SMD between Father-daughter by study design

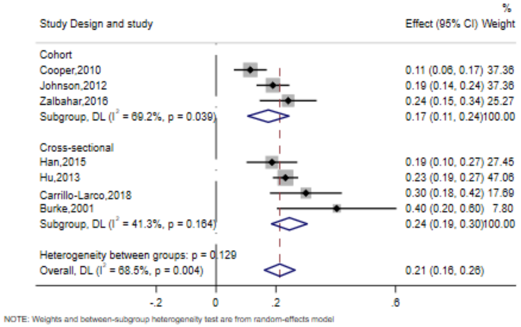

SMD between Father-son by study design

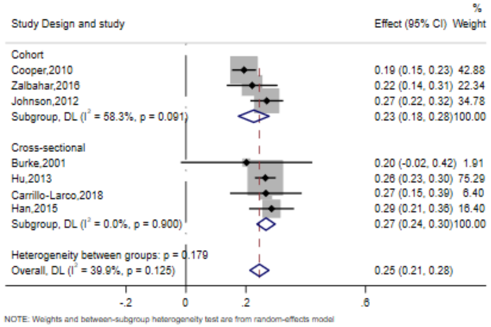

SMD-standardized mean difference

Figure S10. Standardized mean difference between parent-offspring BMI association-subgroup analyses by maternal BMI measurement time

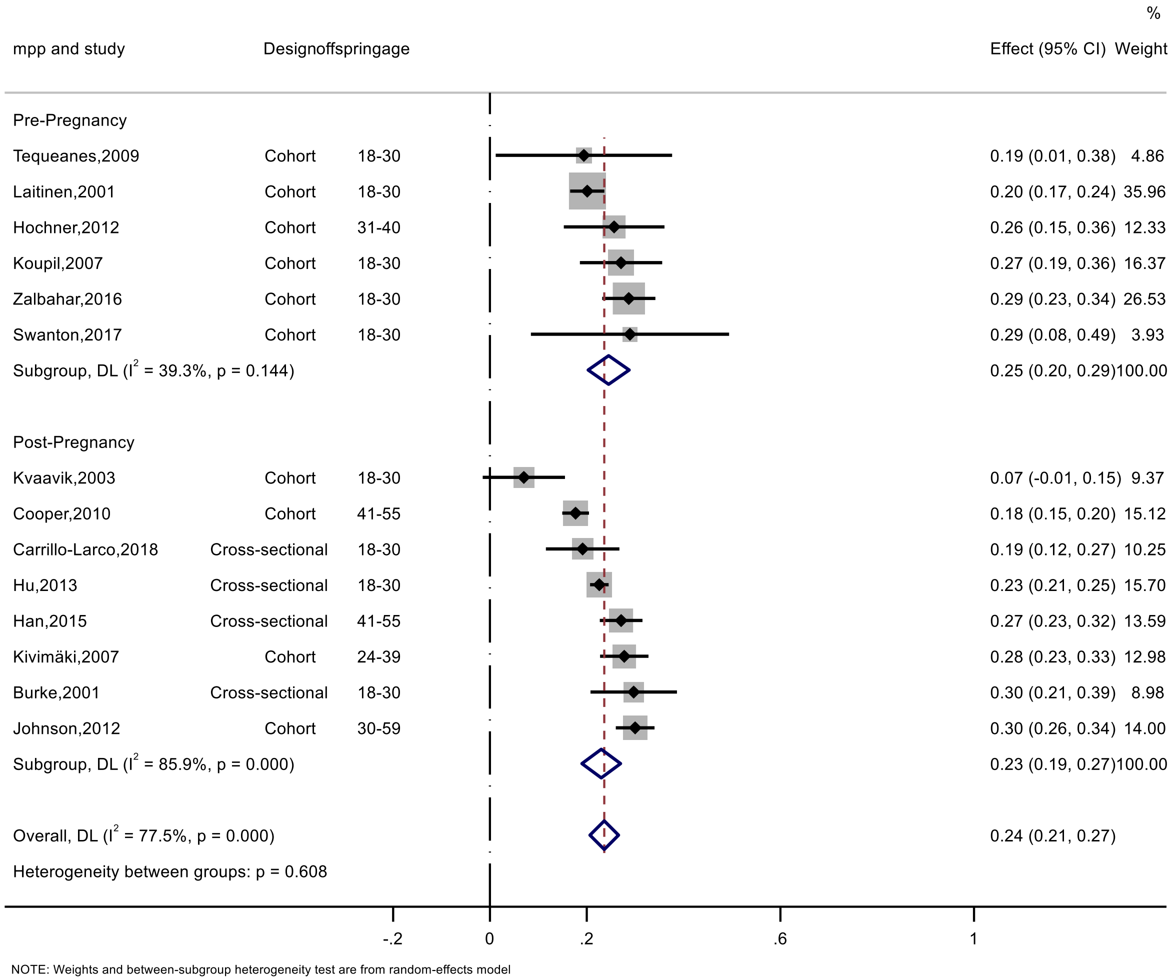

SMD-standardized mean difference, mpp-maternal pre-pregnancy BMI

Figure S11a. Standardized mean difference between parent-offspring BMI association-subgroup analyses by offspring age

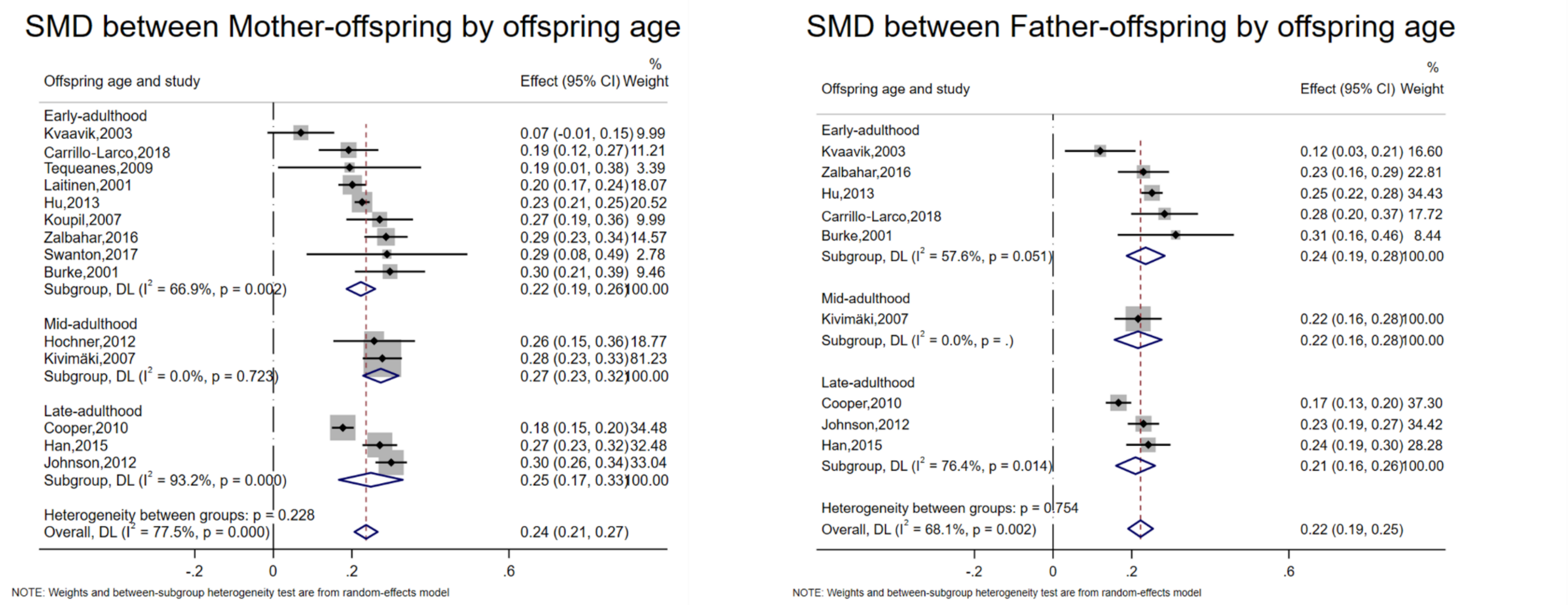

SMD-Standardized mean difference

*Early adulthood: 18-30y; Mid-adulthood: 25-39y or 30-40y; Late adulthood: >40y

Figure S11b. Standardized mean difference between parent-offspring BMI association-subgroup analyses by offspring age (sex-specific level)

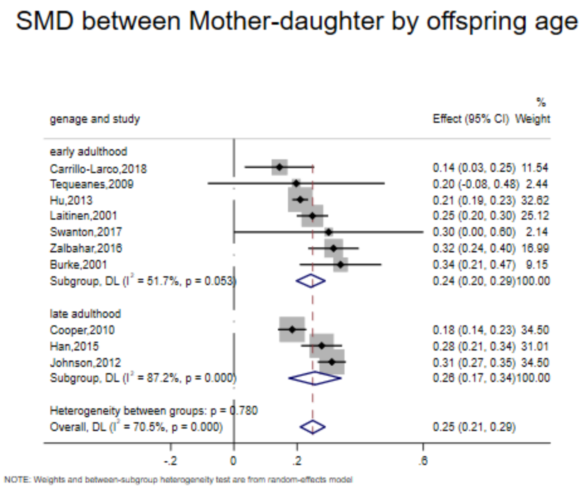

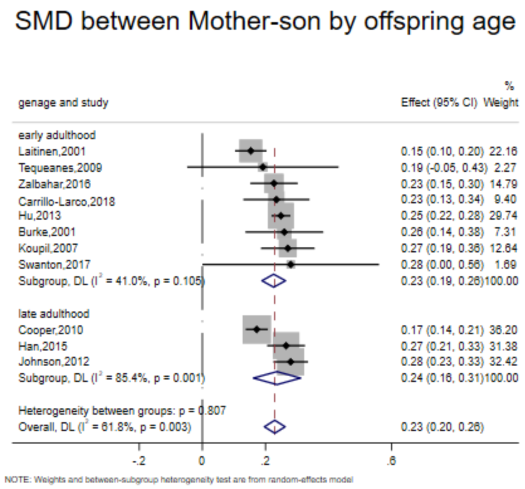

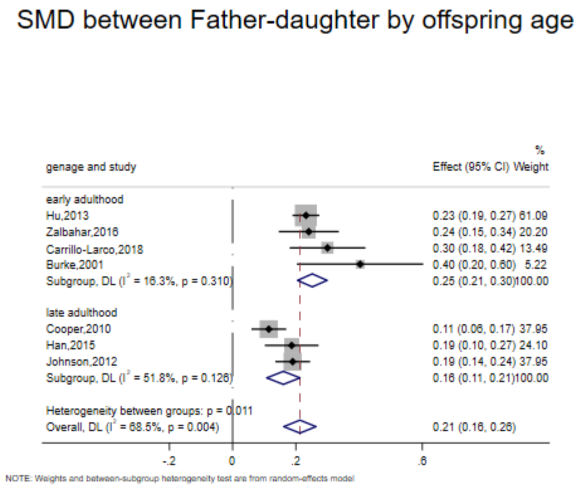

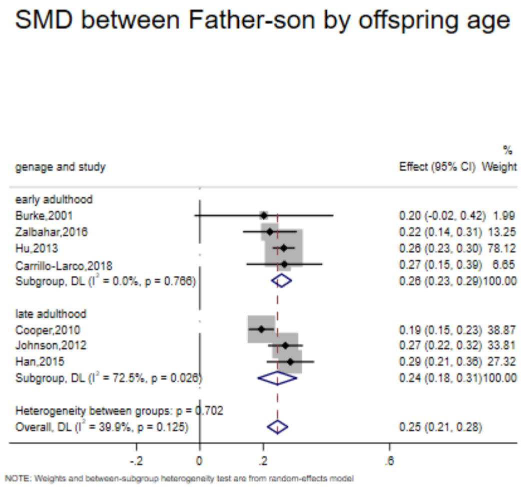

SMD-standardized mean difference

Figure 12a. Standardized mean difference between parent-offspring BMI association-subgroup analyses by parent-offspring BMI assessment time*

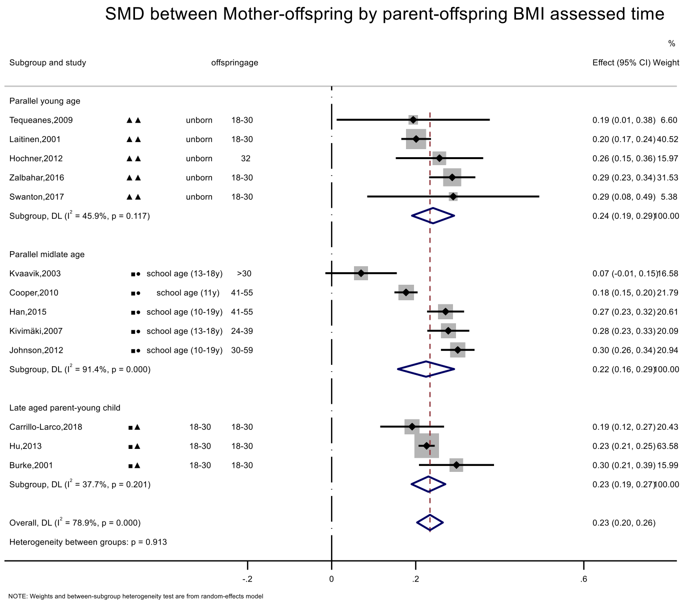

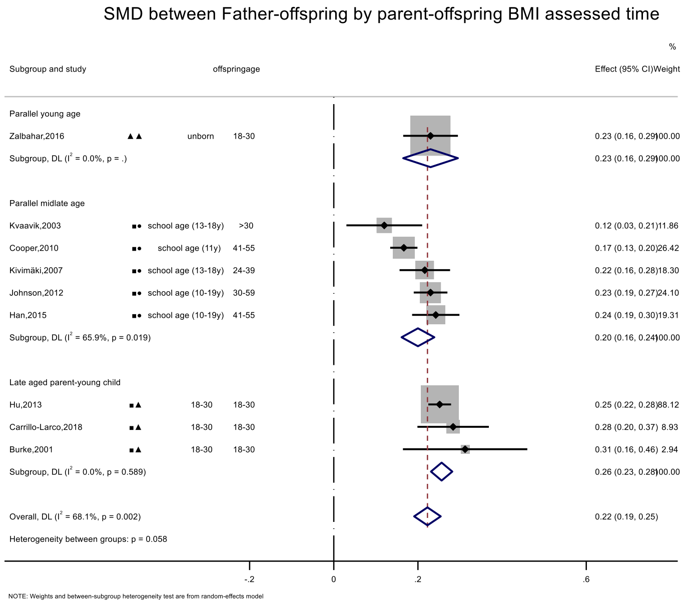

▲ BMI assessed at early age (<=30 y)

● BMI assessed at middle age (30-45 y)

■ BMI assessed at mid/late age (>45 y)

*the parent-offspring BMI assessed time pattern was created based on both parent and offspring's BMI assess time,

The column 'offspringage' in the figure shows child's age when their parents' BMI was assessed, following by child's age when their BMI was assessed.

▲▲ indicates parallel young age (both parent and offspring BMI assessed younger than 30 y)

■● indicates parallel mid/late age (both parent and offspring BMI assessed around mid/late adulthood)

■▲ indicates late aged parent-young child (offspring BMI assessed at young age, while parental BMI at late adulthood)

Figure 12b. Standardized mean difference between parent-offspring BMI association-subgroup analyses by parent-offspring BMI assessment time (sex-specific level)

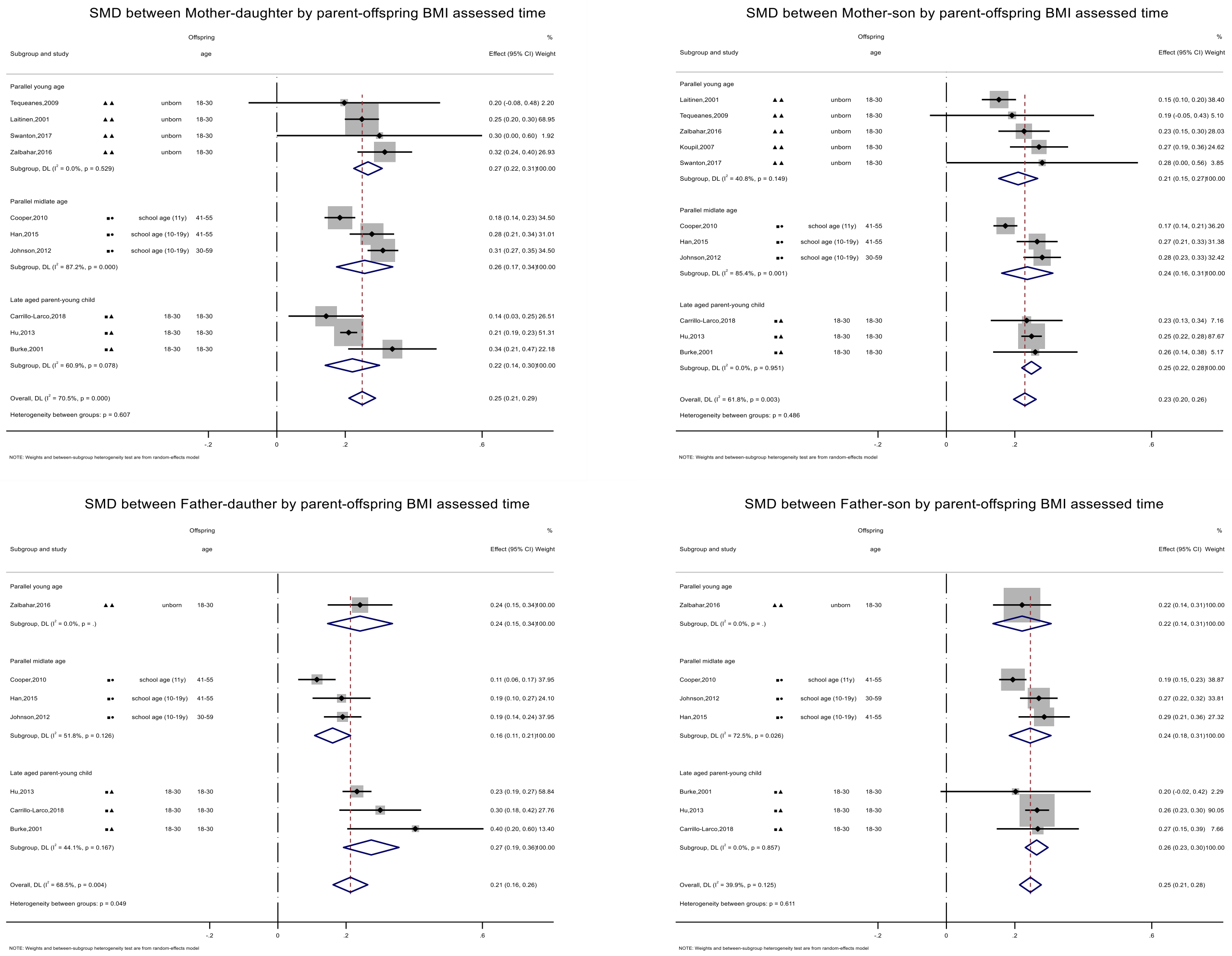

▲ BMI assessed at early age (<=30 y)

● BMI assessed at middle age (30-45 y)

■ BMI assessed at mid/late age (>45 y)

*the parent-offspring BMI assessed time pattern was created based on both parent and offspring's BMI assess time,

The column 'offspringage' in the figure shows child's age when their parents' BMI was assessed, following by child's age when their BMI was assessed.

▲▲ indicates parallel young age (both parent and offspring BMI assessed younger than 30 y)

■● indicates parallel mid/late age (both parent and offspring BMI assessed around mid/late adulthood)

■▲ indicates late aged parent-young child (offspring BMI assessed at young age, while parental BMI at late adulthood)

Figure S13. Forest plot showing odds ratio (OR) of offspring with overweight with parental weight status

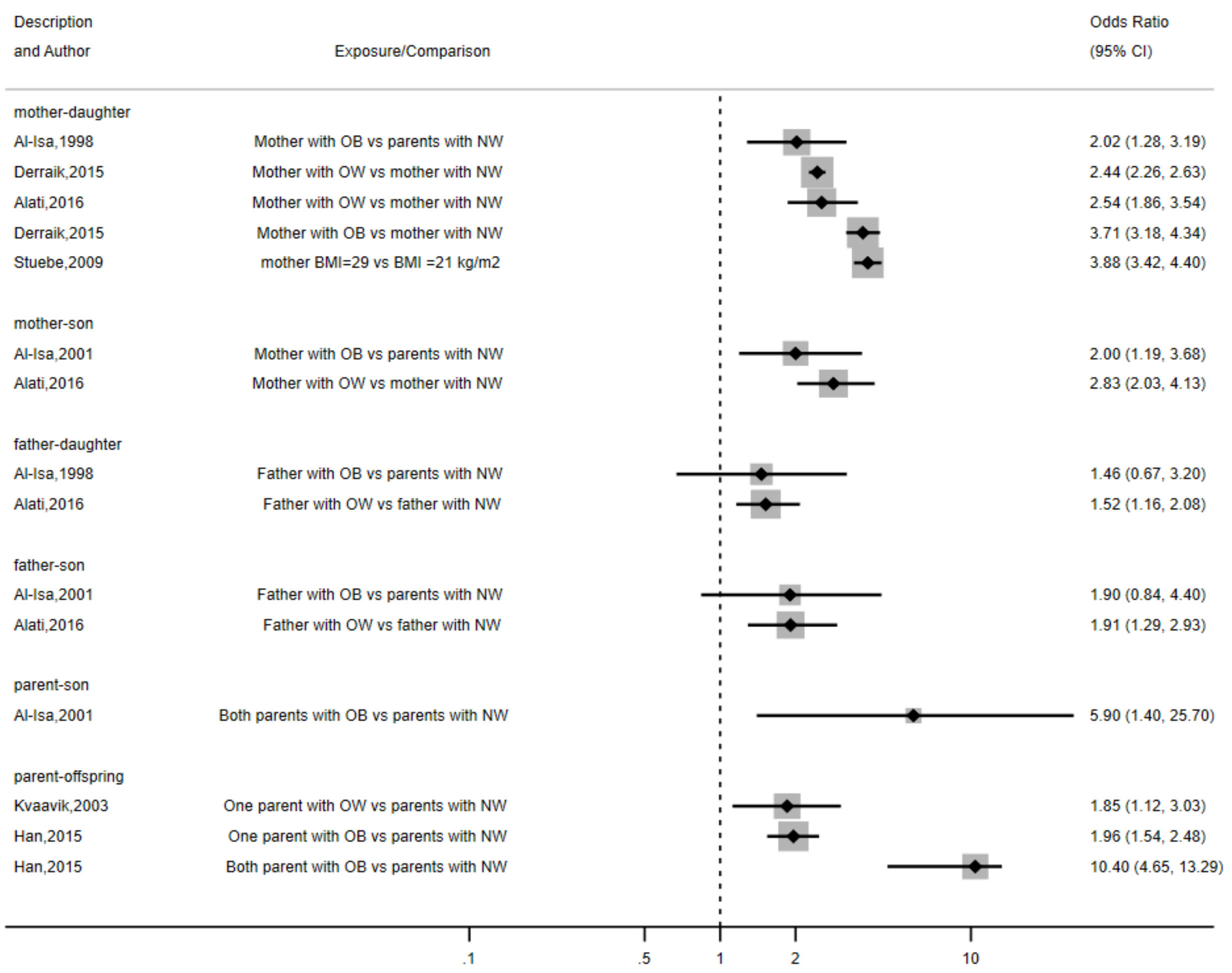

OB-obesity, OW-overweight, NW-normal weight

Figure S14. Forest plot showing odds ratio (OR) of offspring with obesity with parental weight status

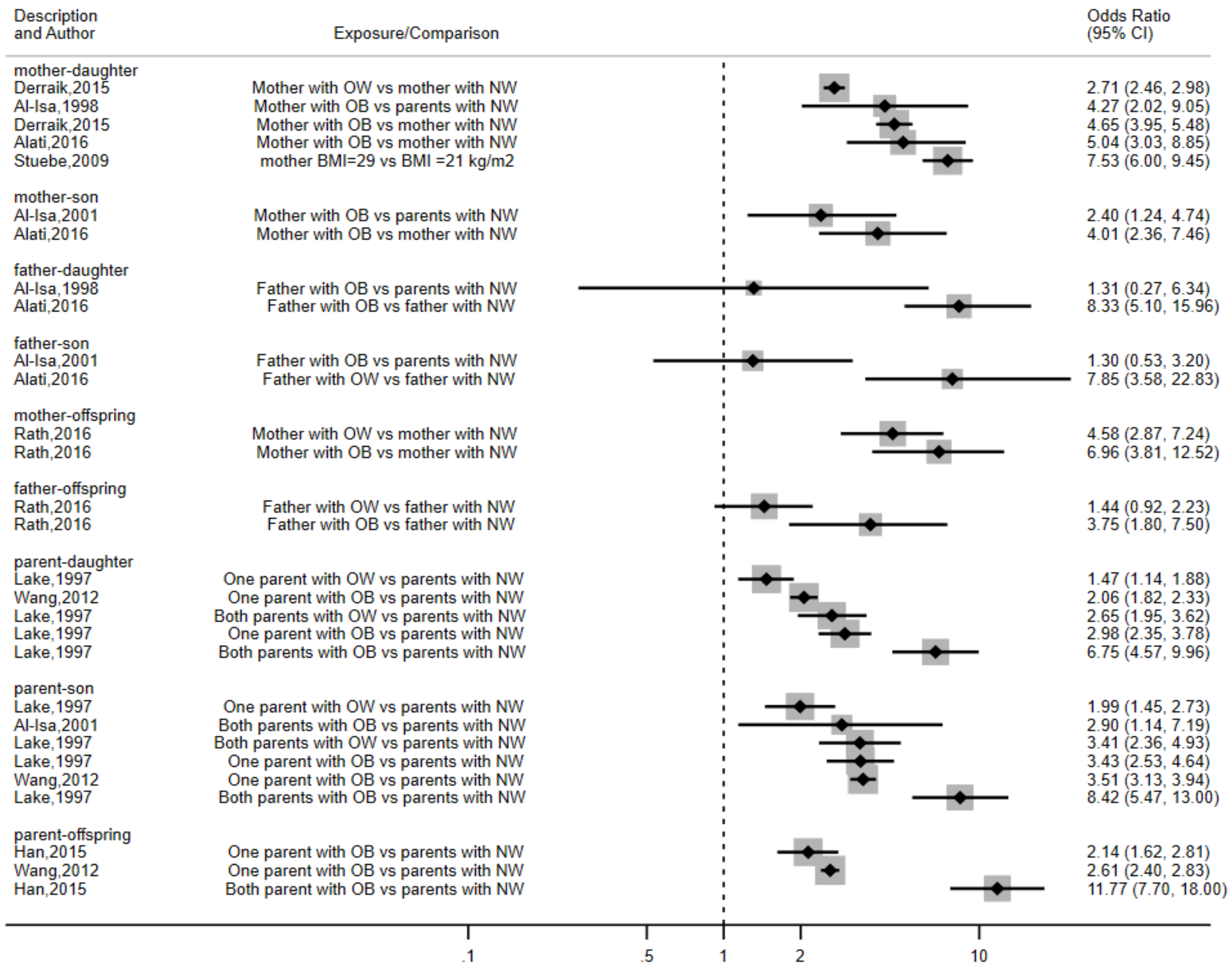

OB-obesity, OW-overweight, NW-normal weight

Figure S15. Forest plot showing odds ratio (OR) of offspring with overweight or obesity with parental weight status

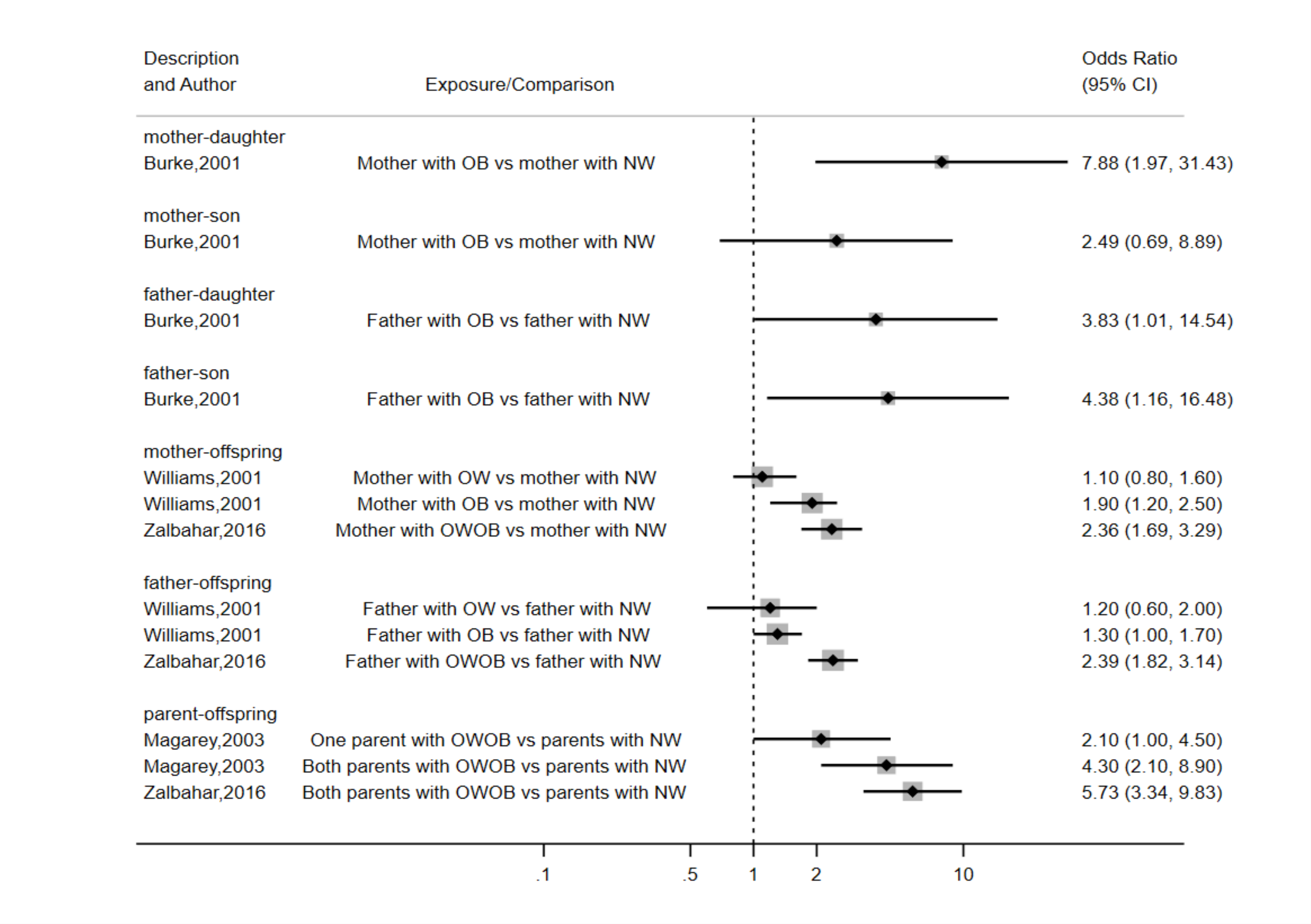

OB-obesity, OW-overweight, NW-normal weight, OWOB-overweight or obesity

Figure S16. Funnel plot for publication bias

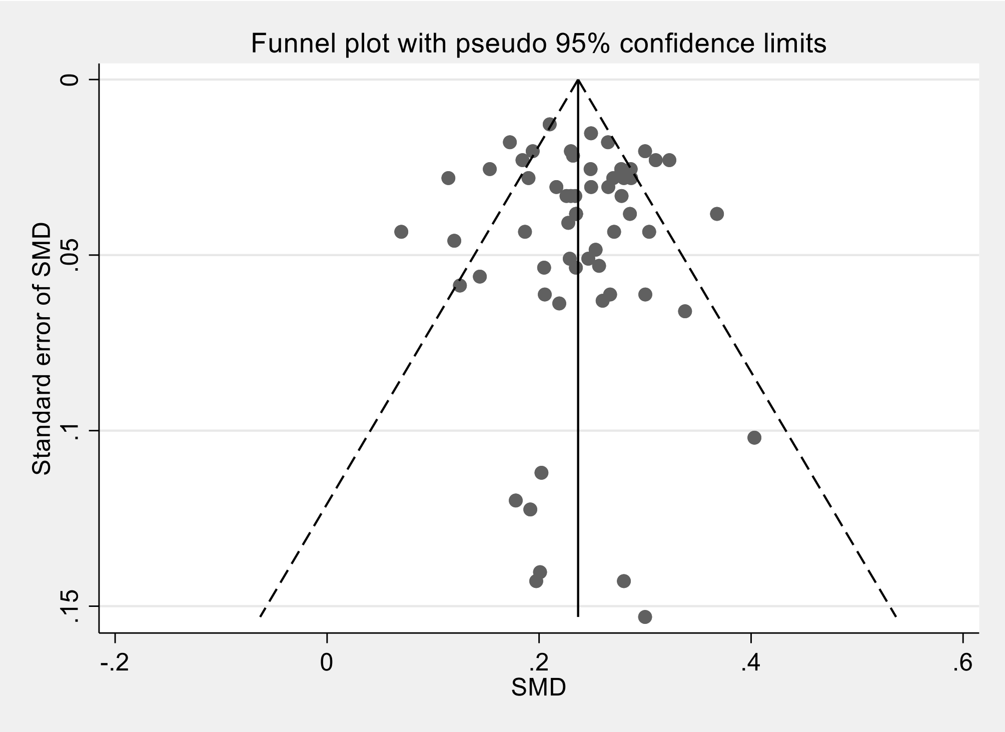

SMD-standardized mean difference

Table S1. Description of all the studies included in systematic review and meta-analyses

Author, publication year, country	Title	Data source	Study design	Sample size	Parents age when BMI assessed	Offspring age when parental BMI assessed	Offspring age when BMI assessed	Assessment of anthropometrics	Parental variable(s)	Offspring variable(s)	Reported measure-ment of association
Khoury et al.[1] 1983 USA	Parent-offspring and sibling body mass index associations during and after sharing of common household environments: the Princeton School District Family Study	The Princeton School Family Study cohort	Cross-sectional study	877	■ detailed age not provided [assessed between 1976-1979]	Late aged parent-young adult child (child's current age)	▲ above 20 y [assessed between 1976-1979]	NA	the Quetelet index (weight/ height^2), and the Benn index (weight/height^2)	the Quetelet index (weight/ height^2), and the Benn index (weight/height^2)	correlation coefficient
Friedlander et al.[2] 1988 Israel	Familial aggregation of body mass index in ethnically diverse families in Jerusalem	The Jerusalem LRC study population	Cross-sectional study	2,942 nuclear family	■ detailed age not provided [assessed between 1976-1979]	Late aged parent-young adult child (child's current age)	▲ Above 17 y [assessed between 1976-1979]	M:Measured F: Measured C: Measured	BMI continuous	BMI continuous	correlation coefficient
Sørensen et al.[3] 1992 Denmark	Correlations of body mass index of adult adoptees and their biological and adoptive relatives	The non-familial adoptions granted in the Copenhagen area	Cross-sectional study	3,476	■ detailed age not provided	Late aged parent-middle aged child (child's current age)	● 42.2 (8.1)	NA	BMI continuous	BMI continuous	correlation coefficient
Rotimi et al.[4] 1995 USA	Familial resemblance for anthropometric measurements	Survey sample from Maywood, IL	Cross-sectional study	162	■ M:46.8(12.50) F:49.10(12.40)	Late aged parent-young adult child (child's current age)	▲ D: 25.40(7.80) S: 26.60(6.30) [assessed in 1990]	M:Measured F: Measured C: Measured	BMI, WHcR, WC, HC as continuous	BMI, WC, HC, WHR as continuous	correlation coefficient

Author, publication year, country	Title	Data source	Study design	Sample size	Parents age when BMI assessed	Offspring age when parental BMI assessed	Offspring age when BMI assessed	Assessment of anthropometrics	Parental variable(s)	Offspring variable(s)	Reported measure-ment of association
	and relative fat distribution among African Americans. International journal of obesity and related metabolic disorder				[assessed in 1990]						
Lake et al.[5] 1997 UK	Child to adult body mass index in the 1958 British birth cohort: associations with parental obesity	The 1958 British birth cohort	Cohort study	12,747	● detailed not provided [assessed in 1969]	Middle aged parent-young child (child aged 11 years at parental measurement)	● 23 and 33 * [assessed between 1991-1992]	M:Self-reported F:Self-reported (at offspring age 11) C:Measured (33 y)	Underweight, Normal, Overweight, or obesity, as defined by the 85th centile &continuous	Binary: Obese vs normal &continuous	ORs correlation coefficient
Al-Isa et al.[6] 1998 Kuwaiti	Factors associated with overweight and obesity among Kuwaiti college women	Random sample from Kuwaiti college women	Cross-sectional study	585	■ detailed age not provided [assessed in 1997]	Late aged parent-young adult child (child's current age)	▲ 18-23 y [assessed in 1997]	M: Measured F: Self-reported C: Measured	WHO criteria: Underweight Normal Overweight Obesity	Binary: OW/OB vs normal	ORs
Burke et al.[7] 2001 Australia	Family lifestyle and parental body mass index as predictors of body mass index in Australian children: a longitudinal study	A cohort of Perth children	Cross-sectional study	219 families (104 sons, and 115 daughters)	■ (F:52.1 (0.4) M:49.1(0.3))	Late aged parent-young adult child (child aged 18 years at parental measurement)	▲ S: 18.1(0.01) D: 18.1(0.01) assessed in 199, Cohort info reference: [8])	M: Measured F: Measured C: Measured	The National Heart Lung and Blood Institute: Normal Overweight Obesity	OW/OB (BMI>25 kg/m^2) vs BMI <25 kg/m^2	β (per kg/m^2) ORs
Williams et al.[9] 2001	Overweight at age 21: the association	Birth cohort born in	Cohort study	924	● detailed age not provided	Middle aged parent-young child	▲	M: Self-reported	WHO criteria: Underweight Normal	OW/OB defined as	RRs correlation coefficient

Author, publication year, country	Title	Data source	Study design	Sample size	Parents age when BMI assessed	Offspring age when parental BMI assessed	Offspring age when BMI assessed	Assessment of anthropometrics	Parental variable(s)	Offspring variable(s)	Reported measure-ment of association
New Zealand	with body mass index in childhood and adolescence and parents' body mass index. A cohort study of New Zealanders born in 1972–1973	Dunedin, New Zealand			[Assessed between 1983-1984]	(child aged 11 years at parental measurement)	21 y [assessed between 1993-1994]	F: Self-reported<br>C: Measured	Overweight<br>Obesity	BMI>25 kg/m^2<br>Normal defined as BMI <25 kg/m^2<br>&<br>continuous	
Laitinen et al.[10]<br>2001<br>Finland<br>ϕ	Family social class, maternal body mass index, childhood body mass index, and age at menarche as predictors of adult obesity	The northern Finland Birth Cohort for 1966	Cohort study	6,280	▲<br>Pre-pregnancy (M: around 27 y at antenatal visit [assessed in 1966]	Similar young adult age at measurement (child unborn at parental measurement)	▲<br>(S:25.2 (3.6)<br>D: 23.8(4.4))<br>[Assessed between 1997-1998]	M: health record<br>C: Self-reported	BMI continuous	WHO criteria:<br>Underweight<br>Normal<br>Overweight<br>Obesity<br>&continuous	β (per kg/m^2)
Magnusson et al.[11]<br>2002<br>Sweden<br>ϕ	Familial resemblance of body mass index and familial risk of high and low body mass index. A study of young men in Sweden	The Swedish Multi-Generation Register	Cohort study	22,517	▲<br>Pre-pregnancy (Around 18-19 y)	Parallel young age<br>(child unborn at parental measurement)	▲<br>18-19 y	F: measured	BMI continuous	BMI continuous	correlation coefficient
Salces et al.[12]<br>2002<br>Spain	Family resemblance for anthropometric traits II. Assessment of maternal	Nuclear families from the province of Biscay	Cross-sectional study	1,326	■<br>F:22-66 y<br>M:22-62 y	Late aged parent-young adult child<br>(child current age)	▲<br>after puberty (only above 18 y were included in the review)	M: Measured<br>F: Measured<br>C: Measured	BMI continuous	BMI continuous	correlation coefficient

Author, publication year, country	Title	Data source	Study design	Sample size	Parents age when BMI assessed	Offspring age when parental BMI assessed	Offspring age when BMI assessed	Assessment of anthropometrics	Parental variable(s)	Offspring variable(s)	Reported measure-ment of association
	occupational and age effects										
Mirmiran et al.[13] 2002 Iran	Familial clustering of obesity and the role of nutrition: Tehran Lipid and Glucose Study. International journal of obesity	The Tehran Lipid and Glucose Study (TLGS)	Cross-sectional study	474	■ M:40.6 (0.5) F:47.4 (0.5) [assessed between 1999-2001]	Late aged parent-young adult child (child's current age)	▲ 18-25 y [assessed between 1999-2001]	M: Measured F: Measured C: Measured	Overweight ≥25 kg/m^2	Overweight ≥25 kg/m^2 (aged≥20)	correlation coefficient ORs***
Kvaavik et al.[14] 2003 Norway	Predictors and tracking of body mass index from adolescence into adulthood: follow-up of 18 to 20 years in the Oslo Youth Study	The Oslo Youth Study	Cohort study	485	■ F: 45.8 (7.0) M: 42.3 (6.3) (assessed in baseline survey 1981)	Similar mid/late age at measurement (child aged 13-18 years at parental measurement)	● 32.4(1.0) [assessed in 1999]	M: Self-reported F: Self-reported C: Self-reported	BMI≥25 kg/m^2 vs BMI<25 kg/m^2	BMI≥25 kg/m^2 Vs BMI<25 kg/m^2 &continuous	β (per sd) ORs
Wu et al.[15] 2003 Taiwan	Familial resemblance of adiposity-related parameters: results from a health check-up population in Taiwan	The Mei-Jou Health Screening program	Cross-sectional study	1,724	■ F: 58.2 (8.5) M: 55.3 (8.5) [assessed in 1997]	Late aged parent-young adult child (child's current age)	▲ S: 30.1 (8.2) D: 28.5 (7.2) [assessed in 1997]	M:Measured F: Measured C: Measured	BMI as continuous	BMI as continuous	correlation coefficient
Magarey et al.[16] 2003 Australia	Predicting obesity in early adulthood from childhood and parental obesity	Sample selected from healthy term infants	Cohort study	188	● detailed age not provided [assessed 1983-1984]	Middle aged parent-young adult child (child aged 8 years at	▲ 20 y [assessed 1995-1996]	M: Measured F: Measured C: Measured	Acceptable weight <25 kg/m^2, overweight ≥25 and <30 kg/m^2	Acceptable weight <25 kg/m^2, overweight ≥25 and <30 kg/m^2	correlation coefficient ORs

Author, publication year, country	Title	Data source	Study design	Sample size	Parents age when BMI assessed	Offspring age when parental BMI assessed	Offspring age when BMI assessed	Assessment of anthropometrics	Parental variable(s)	Offspring variable(s)	Reported measure-ment of association
		born in Adelaide, South Australia				parental measurement)			and obesity ⩾ 30 kg/m^2	and obesity ⩾30 kg/m^2	
Kazumi et al.[17] 2005 Japan	Associations of middle-aged mother's but not father's body mass index with 18-year-old son's waist circumferences, birth weight, and serum hepatic enzyme levels	Male college students from Kobe University of Mercantile Marine	Cross-sectional study	195	■ F: 49 (4) M: 46 (3) [assessed in 2019]	Late aged parent-young adult child (child's current age)	▲ 18y [assessed in 2019]	M: Self-reported F: Self-reported C: Measured	BMI continuous	BMI continuous	Correlation coefficient
Crossman et al.[18] 2006 USA	The family environment and American adolescents' risk of obesity as young adults	The United States National Longitudinal Study of Adolescent Health	Cross-sectional study	6,378	● detailed age not provided [assessed between 1995]	Middle aged parent-young adult child (child aged 6-8 y at parental measurement)	▲ 18-26 y [assessed between 2001-2002]	M: Self-reported F: Self-reported C: Self-reported	WHO criteria: Underweight Normal Overweight Obesity	CDC's adult guidelines Binary: OW/OB	ORs
Kivimäki et al.[19] 2007 Finland	Substantial intergenerational increases in body mass index are not explained by the fetal overnutrition hypothesis: the Cardiovascular Risk in Young Finns Study	The Young Finns Study	Cohort study	1,918	■ F: 40.0 (8.4) M: 37.5 (7.6) [assessed in 1980]	Middle aged parent-young adult child (child aged 3-18y at parental measurement)	● 24-39 y [assessed in 2001]	M: Self-reported F: Self-reported C: Measured	BMI continuous	BMI continuous	β (per kg/m^2)

**Author, publication year, country**	**Title**	**Data source**	**Study design**	**Sample size**	**Parents age when BMI assessed**	**Offspring age when parental BMI assessed**	**Offspring age when BMI assessed**	**Assessment of anthropometrics**	**Parental variable(s)**	**Offspring variable(s)**	**Reported measure-ment of association**
Abu-Rmeileh NM et al.[20] 2008 Scotland	Contribution of Midparental BMI and other determinants of obesity in adult offspring	The Renfrew and Paisley	Cohort study	2,162	■ 45-64 y [assessed between 1972-1976]	Late aged parent-mid/late adulthood child (child was around 10-39 years old at parental measurement)	■● 30-59 y [assessed in 1996]	M:Measured F:Measured C:Measured	WHO criteria: Underweight Normal Overweight Obesity &the mean of parental BMI	WHO criteria: Underweight Normal Overweight Obesity &continuous	β (per kg/m^2)
Koupil et al.[21] 2008 Sweden ϕ	Social and early-life determinants of overweight and obesity in 18-year-old Swedish men	The Uppsala Birth Cohort	Cohort study	1,103	▲ Linked to register data to get mother's pre-pregnancy BMI, detailed age not provided	Similar young adult age at measurement (child unborn at parental measurement)	▲ 18 y (range:18-23)	M: Self-reported S: Measured	WHO criteria: Underweight Normal Overweight Obesity &continuous	WHO criteria: Underweight Normal Overweight Obesity &continuous	β (per kg/m^2) ORs
Tequeanes et al.[22] 2009 Brazil ϕ	Maternal anthropometry is associated with the body mass index and waist: height ratio of offspring at 23 years of age	1982 Pelotas Birth Cohort Study	Cohort study	2,978	▲ Pre-pregnancy (detailed age not provided)	Similar young adult age at measurement (child unborn at parental measurement)	▲ 23y (assessed in 1982)	M: Self-reported C: Measured	BMI Continuous	BMI Continuous	β (per kg/m^2)
Kowaleski-Jones et al.[23] 2009 USA	Are you what your mother weighs? Evaluating the impact of maternal weight trajectories on youth overweight	The National Longitudinal Survey of Youth 1979 Cohort	Cross-sectional study	1,759	■ 39-47 y [assessed in 2004]	Late age parent-young adult child (child's current age)	▲ 16-21 y [assessed in 2006]	M: Self-reported and measured C: Self-reported and measured	BMI continuous	Overweight: The CDC BMI-for-age	ORs

Author, publication year, country	Title	Data source	Study design	Sample size	Parents age when BMI assessed	Offspring age when parental BMI assessed	Offspring age when BMI assessed	Assessment of anthropometrics	Parental variable(s)	Offspring variable(s)	Reported measure-ment of association
Stuebe et al.[24] 2009 USA ϕ	Maternal-recalled gestational weight gain, pre-pregnancy body mass index, and obesity in the daughter	The Nurses' Healthy Study and the Nurses' Mothers' Cohort	Cohort	26,506 mother-nurse daughter dyads	▲ Detailed age not provided [assessed in 2001]	Similar young age at measurement (Used recalled pre-pregnancy BMI, child unborn at parental measurement)	▲ ● 25-44 or 36-56 [assessed in 1989 and 2001]	M: Self-reported D: Self-reported	Pre-pregnancy BMI: 21 kg/m^2, 23 kg/m^2, 25 kg/m^2, 27 kg/m^2, 29 kg/m^2	Overweight as BMI⩾25 and <30 kg/m^2 and obesity as BMI⩾30 kg/m^2	ORs
Classen et al.[25] 2010 USA ϕ	Measures of the intergenerational transmission of body mass index between mothers and their children in the United States, 1981–2004	The National Longitudinal Survey of Youth 1979 (NLSY79) and the Children and Young Adults of the NLSY79 (YA NLSY79)	Cross-sectional study	4,748	▲ 16-24 y [assessed in 1986]	Similar young adult age at measurement (child unborn at parental measurement)	▲ 16-24 y [assessed in 1994]	M: Measured C: Measured	WHO criteria: Underweight Normal Overweight Obesity	BMI continuous	correlation coefficient
Cooper et al.[26] 2010 UK	Associations between parental and offspring adiposity up to midlife: the contribution of adult lifestyle factors in the 1958 British Birth Cohort Study	The 1958 British birth cohort	Cohort study	9,346	■ detailed age was not provided [assessed in 1969]	Similar mid/late age at measurement (child was 11 years old at parental measurement)	● 44-45y [assessed in 2002-2003]	M:Self-reported F:Self-reported (when offspring were aged 11) C:Measured	WHO criteria: Underweight Normal Overweight Obesity	BMI continuous	β (per kg/m^2) correlation coefficient ORs

Author, publication year, country	Title	Data source	Study design	Sample size	Parents age when BMI assessed	Offspring age when parental BMI assessed	Offspring age when BMI assessed	Assessment of anthropometrics	Parental variable(s)	Offspring variable(s)	Reported measure-ment of association
Reynolds et al.[27]<br>2010<br>UK<br>*ϕ*	Maternal BMI, parity, and pregnancy weight gain: influences on offspring adiposity in young adulthood	The Motherwell birth chort study	Cohort study	276	▲<br>27.2 (6.0) [assessed 1967-1968]	Similar young adult age at measurement (child unborn at parental measurement)	▲<br>27-30 y [assessed in 1994-1998]	M: antenatal records<br>C: Measured	BMI continuous	BMI continuous AND binary outcome: overweight (BMI >25kg/ m^2)	correlation coefficient<br>ORs
Al-Isa et al.[28]<br>2011<br>Kuwaiti	Factors associated with overweight and obesity among Kuwaiti men	Kuwaiti men, samples from ambulatory patients visiting a clinic in the capital	Cross-sectional study	464	■<br>detailed age not provided	Late aged parent-young adult child (child's current age)	▲<br>Above 20 y	M: Reported<br>F: Reported<br>C: Measured	WHO criteria:<br>Underweight<br>Normal<br>Overweight<br>Obesity	WHO criteria:<br>Underweight<br>Normal<br>Overweight<br>Obesity	ORs
Hochner et al.[29]<br>2012<br>Israel<br>*ϕ*	Associations of maternal prepregnancy body mass index and gestational weight gain with adult offspring cardiometabolic risk factors: the Jerusalem Perinatal Family Follow-up Study	The Jerusalem Perinatal Study (JPS) population-based cohort	Cohort study	1,256	▲<br>M:<br>28.38 (5.47) [assessed between 1974-1976]	Similar young adult age at measurement (child unborn at parental measurement)	▲<br>32 y [assessed between 2007-2009]	M: Self-reported<br>C: Measured	mppBMI(quartile)<br>1:<21.0kg/ m^2<br>2:21.0-23.8kg/ m^2<br>3:23.9-26.4kg/ m^2<br>4:>26.4kg/ m^2<br>&continuous	BMI Continuous	β (per kg/ m^2)
Johnson et al.[30]<br>2012<br>UK	Intergenerational change and familial aggregation of	The Midspan Renfrew and	Cohort study	3,729	■<br>Above 45 y	Similar mid/late age at measurement	●■<br>30-59 y [assessed in 1996]	M: Measured<br>F: Measured<br>C: Measured	WHO criteria:<br>Underweight<br>Normal<br>Overweight	BMI continuous	Standardized β (per SD)

Author, publication year, country	Title	Data source	Study design	Sample size	Parents age when BMI assessed	Offspring age when parental BMI assessed	Offspring age when BMI assessed	Assessment of anthropometrics	Parental variable(s)	Offspring variable(s)	Reported measure-ment of association
ϕ	body mass index	Paisley Study, and Midspan Family Study			[assessed between 1972-1976]				Obesity		
Murrin et al.[31]<br>2012<br>Ireland**	Body mass index and height over three generations: evidence from the Lifeways cross-generational cohort study	The Lifeways study	Cohort study	529	■<br>MGM: 60.50 (8.54)<br>MGF:63.33 (9.09)<br>PGM:62.10(8.74)<br>PGF: 62.85 (10.10)<br>[assessed between 2001-2003]	Late aged parent-middle aged child (child's current age)	●<br>M:30.85 (5.70)<br>F:34.43 (5.65)<br>[assessed between 2001-2003]	MGM, MGF, PGM, PGF: Self-reported<br>M: Self-reported<br>F: Self-reported	WHO criteria:<br>Underweight<br>Normal<br>Overweight<br>Obesity<br>&BMI continuous	WHO criteria:<br>Underweight<br>Normal<br>Overweight<br>Obesity<br>&BMI continuous	correlation coefficient
Wang et al.[32]<br>2012<br>China	Epidemiology of general obesity, abdominal obesity and related risk factors in urban adults from 33 communities of Northeast China: the CHPSNE study	The CHPSNE study(Control Hypertension and Other Risk Factors to Prevent Stroke with Nutrition Education in Urban Area of Northeast China)	Cross-sectional study	25,196	■<br>detailed age not provided, [assessed in 2009-2010]	Late aged parent-middle aged child (child's current age)	●<br>41.7 (14.4) [assessed in 2009-2010]	M: Interview or clinical measure<br>F: Interview or clinical measure<br>C: Interview or clinical measure	WHO Chinese criteria:<br>Underweight<br>Normal<br>Overweight (⩾ 25 and <27.5 kg/m^2)<br>Obesity (⩾27.5 kg/m^2)	WHO Chinese criteria:<br>Underweight<br>Normal<br>Overweight (⩾25 and <27.5)<br>Obesity (⩾27.5 kg/m^2)<br>And WHO criteria for Europids (comparable to other studies)	ORs

Author, publication year, country	Title	Data source	Study design	Sample size	Parents age when BMI assessed	Offspring age when parental BMI assessed	Offspring age when BMI assessed	Assessment of anthropometrics	Parental variable(s)	Offspring variable(s)	Reported measure-ment of association
Hu et al.[33]<br>2013<br>China	Familial correlation and aggregation of body mass index and blood pressure in Chinese Han population	The China National Nutrition and Health Survey 2002	Cross-sectional study	19,107	■<br>F: 52.0 (6.2)<br>M: 49.8 (5.8)<br>[assessed in 2002]	Late aged parent-young adult child (child's current age)	▲<br>S:24.8 (5.0)<br>D: 23.0 (4.7)<br>[assessed in 2002]	M:Measured<br>F: Measured<br>C: Measured	Overweight ⩾ 25 and <30 kg/m^2<br>Obesity ⩾30 kg/m^2<br>&continuous	Overweight ⩾25 and <30 kg/m^2<br>Obesity ⩾30 kg/m^2<br>&continuous	Standarized β(per SD)
Kelly et al.[34]<br>2014<br>Ireland*	Body mass index is associated with the maternal lines but height is heritable across family lines in the Lifeways Cross-Generation Cohort Study	The Lifeways Cross-Generation Cohort Study	Cohort study	556 families	■<br>NA<br>[assessed 2001-2014]	Late aged parent-middle aged child (child's current age)	●<br>NA<br>[assessed 2001-2014]	MGM, MGF, PGM, PGF: Self-reported and measured mixed<br>M: Self-reported<br>F: Self-reported	BMI continuous	BMI continuous	correlation coefficient
Vik et al.[35]<br>2014<br>Norway	Comparison of father-offspring and mother-offspring associations of cardiovascular risk factors: family linkage within the population-based HUNT Study, Norway	The HUNT study	Cross-sectional study	36,528 (father-mother-offspring trios)	■<br>M: 59.4 (12.5)<br>F: 61.7 (12.4)	Late aged parent-middle aged child (child's current age)	●<br>35.6(10.6)	M: Measured<br>F: Measured<br>C: Measured	BMI continuous	BMI continuous	β<br>(Per kg/m^2)
Cho et al.[36]<br>2015<br>USA	Comparisons of chewing rhythm, craniomandibu	Sample from local high schools	Cross-sectional study	32 mother–daughter pairs	■<br>49.9 (5.5)	Late aged parent-young adult child	▲<br>17.3 (2.2)	NA	BMI continuous	BMI continuous	correlation coefficient

Author, publication year, country	Title	Data source	Study design	Sample size	Parents age when BMI assessed	Offspring age when parental BMI assessed	Offspring age when BMI assessed	Assessment of anthropometrics	Parental variable(s)	Offspring variable(s)	Reported measure-ment of association
	lar morphology, body mass and height between mothers and their biological daughters. Archives of Oral Biology	and the University of Michigan School of Dentistry				(child's current age)					
Derraik et al.[37] 2015 Sweden ϕ	Obesity rates in two generations of Swedish women entering pregnancy, and associated obesity risk among adult daughters.	Retrospective cohort from the Swedish Birth Register	Cohort study	26,561 pairs	▲ 26.2 (5.0) [1982-1988]	Similar young adult age at measurement (child unborn at parental measurement)	▲ 22.4 (2.3) [2000-2008]	M: Weight was measured and current height was self-reported or measured D: same	WHO criteria: Underweight Normal Overweight Obesity	WHO criteria: Underweight Normal Overweight Obesity	ORs
Han et al.[38] 2015 Scotland	Contributions of maternal and paternal adiposity and smoking to adult offspring adiposity and cardiovascular risk: the Midspan Family Study	The Midspan Family Study	Cross-sectional study	2,230	■ F: 54.9(5.0) M: 52.8 (4.9) [assessed between 1972-1976]	Similar mid/late age at measurement (child was young at parental measurement)	■ Range: 30–59 S:44.8 (6.3) D:45.2 (6.1) [assessed in 1996]	M: Measured F: Measured C: Measured	WHO criteria: Underweight Normal Overweight Obesity &continuous	WHO criteria: Underweight Normal Overweight Obesity &continuous	β (per kg/m^2) ORs
Eriksson et al.[39] 2015 Finland ϕ	Maternal weight in pregnancy and offspring body composition in late adulthood:	The Helsinki Birth Cohort Study (HBCS)	Cohort study	2,003	▲ 28.6 (5.5) [birth cohort when child born	Young parent-late aged adult child (linked to birth records to get mother's BMI	■ Mean age: 62 y [assessed in 2003]	M: Measured C: Measured	Quartile ⩽24.6 kg/m^2 -26.3 kg/m^2 -28.1 kg/m^2 >28.1 kg/m^2	FM% LM%	β (per kg/m^2)

Author, publication year, country	Title	Data source	Study design	Sample size	Parents age when BMI assessed	Offspring age when parental BMI assessed	Offspring age when BMI assessed	Assessment of anthropometrics	Parental variable(s)	Offspring variable(s)	Reported measure-ment of association
	findings from the Helsinki Birth Cohort Study (HBCS)				between 1934-1944]	prior to delivery)					
Alati et al.[40]<br>2016<br>Australia<br>ϕ	Generational increase in obesity among young women: a prospective analysis of mother–daughter dyad	The Mater University Study of Pregnancy (MUSP)	Cohort study	953 pairs	▲<br>18-25 y<br>[assessed 1981-1983]	Similar young adult age at measurement (child unborn at parental measurement)	▲<br>21 y<br>[assessed 2002-2004]	M: Measured<br>C: Measured	WHO criteria:<br>Underweight<br>Normal<br>Overweight<br>Obesity<br>HW: BMI<25 kg/m^2<br>OW/OB: BMI$\geq$ 25 kg/m^2	WHO criteria:<br>Underweight<br>Normal<br>Overweight<br>Obesity	ORs
Zalbahar et al.[41]<br>2016<br>Australia<br>ϕ	Parental pre-pregnancy BMI influences on offspring BMI and waist circumference at 21 years	The Mater-University of Queensland Study of Pregnancy (MUSP) cohort	Cohort study	2,229 pairs	▲<br>Detailed age not provided [assessed 1981-1983]	Similar young adult age at measurement (child unborn at parental measurement)	▲<br>21 y<br>[assessed 2002-2004]	M: Measured<br>F: Reported by the mother<br>C: Measured	WHO criteria, then collapsed to two groups: Normal weight(<25 kg/m^2) or OW/OB($\geq$25 kg/m^2)	BMI continuous, WHO criteria, then collapsed to two groups: Normal weight(<25 kg/m^2) or OW/OB($\geq$ 25 kg/m^2)	β (per kg/m^2)<br>ORs
Rath et al.[42]<br>2016<br>Australia<br>ϕ	Parental pre-pregnancy BMI is a dominant early-life risk factor influencing BMI of offspring in adulthood	The Western Australian Pregnancy Cohort (Raine) Study	Cohort study	1,355	▲<br>27-28 y[43]<br>[1989-1991]	Similar young adult age at measurement (child unborn at parental measurement)	▲<br>22 y<br>[2011-2013]	M: Measured<br>F: Measured<br>C: Measured	Overweight was defined as BMI $\geq$25 kg/m^2, obesity as BMI $\geq$30 kg/m^2	2000 Center for Disease Control and Prevention growth charts<br>Overweight was defined as BMI z-score $\geq$85th centile, obesity as BMI z-score	ORs

Author, publication year, country	Title	Data source	Study design	Sample size	Parents age when BMI assessed	Offspring age when parental BMI assessed	Offspring age when BMI assessed	Assessment of anthropometrics	Parental variable(s)	Offspring variable(s)	Reported measure-ment of association
										⩾95th centile	
Swanton et al.[44] 2017 USA ϕ	Body mass index associations between mother and offspring from birth to age 18: the Fels Longitudinal Study	The Fels Longitudinal Study	Cohort study	427	▲ M: 30-40 y	Similar young adult age at measurement (child newly born at parental measurement)	▲ 18 y	M: Measured C: Measured	BMI continuous	BMI continuous	correlation coefficient β (per sd)
Rodrigo M. Carrillo-Larco et al.[45] 2018 Peru	Parental body mass index and blood pressure are associated with higher body mass index and blood pressure in their adult offspring: a cross-sectional study in a resource-limited setting in northern Peru	Population-based implementation study	Cross-sectional study	955	■ F: 59 (11.6) M: 54 (11.8) [assessed in 2016]	Late aged parent-young adult child (child's current age)	▲ 29 (9.5) [assessed in 2016]	M: Measured F: Measured C: Measured	BMI continuous	BMI continuous	β (per kg/m^2)
Kaseva et al.[46] 2018 Finland ϕ	Pre-pregnancy overweight or obesity and gestational diabetes as predictors of body composition in offspring twenty years	The ESTER Maternal Pregnancy Disorders Study and the Arvo Ylppö Longitudin	Cohort study	891	▲ At antenatal visit [assessed between 1985-1986]	Similar young adult age at measurement (child unborn at parental measurement)	▲ 24.1 (1.4) y [assessed between 2009-2011]	M: healthcare records & questionnaires C: NA	normoglycemic mothers with pre-pregnancy overweight or obesity; mothers with GDM at any level of maternal BMI; offspring of	BMI continuous	RD

Author, publication year, country	Title	Data source	Study design	Sample size	Parents age when BMI assessed	Offspring age when parental BMI assessed	Offspring age when BMI assessed	Assessment of anthropometrics	Parental variable(s)	Offspring variable(s)	Reported measure-ment of association
	later: evidence from two birth cohort studies	al Study (AYLS)							mothers with pre-pregnancy BMI <25 kg/m^2 and no GDM		
Schoppa et al.[47] 2018 Finland ϕ	Association of Maternal Prepregnancy Weight with Offspring Adiposity Throughout Adulthood over 37 Years of Follow-up	The Framingham Heart Study Offspring cohort	Cohort study	863	▲ Detailed age not provided [assessed in 1948]	Similar young adult age at measurement (child unborn at parental measurement)	▲ 33 (10) y [assessed between 1971-1975]	M: Self-reported or measured C: Measured	Normal weight (BMI < 25 kg/m^2) or OW/OB(BMI ⩾ 25 kg/m^2)	Overweight was defined as BMI 25 to 29.9 kg/m^2, and obesity was defined as BMI ⩾ 30 kg/m^2	RD
Chaparro et al.[48] 2017 Sweden ϕ	Maternal pre-pregnancy BMI and offspring body composition in young adulthood: the modifying role of offspring sex and birth order	The Uppsala Family Study (UFS)	Cohort study	226 (113 sibling pairs)	▲ 27-29 y [assessed between 1987-1995]	Similar young adult age at measurement (child unborn at parental measurement)	▲ 20.2 (15.3-24.6) y [assessed between 2010-2012]	M: Self-reported C: Measured	Maternal pre-pregnancy OW/OB (⩾ 25 kg/m^2)	FM% LM%	β (per kg/m^2)

Maternal (M); Paternal(F); Children (C); Daughter(D); Son(S); MGM, Maternal Grandmother; MGF, Maternal Grandfather; PGM, Paternal Grandmother; PGF, Paternal Grandfather; OW/OB, Overweight or obesity; Offspring OGDM, mother with gestational diabetes; mppBMI, Maternal pre-pregnancy BMI; NA, not available; FM, fat mass; LM, lean mass; WC, waist circumference; WHR, waist height ratio; WHcR, Waist-Hip Circumference Ratio
*results at 33 years were used in this review; **three-generation study, we extracted data for the first two generations (grandparents and parents)
ϕ pre/during pregnancy
▲BMI assessed at young adulthood (<=30 y)
● BMI assessed at middle adulthood (30-45 y)
■ BMI assessed at late adulthood (>45 y)

Table S2. Descriptions of studies reporting correlation coefficient

Study (Author, year)	Title	Methods	Adjusted covariates	Family relationship	r	n
Khoury et al.[1] 1983	Parent-offspring and sibling body mass index associations during and after sharing of common household environments: the princeton school district family study	correlation	NA	father-son	0.104	34
				father-daughter	0.132	36
				mother-son	0.167	43
				mother-daughter	-0.076	49
Friedlander et al.[2] 1988	Familial aggregation of body mass index in ethnically diverse families in Jerusalem. The Jerusalem Lipid Research Clinic	Interclass correlation	NA	father-son	0.272	1770
				mother-son	0.205	1960
				mother-daughter	0.169	1374
				father-daughter	0.206	1243
				parent-offspring	0.217	6347
Rotimi et al.[4] 1995	Familial resemblance for anthropometric measurements and relative fat distribution among African Americans	Intraclass correlation	age	mother-son	0.26	65
				mother-daughter	0.33	86
Lake et al.[5] 1997	Child to adult body mass index in the 1958 British birth cohort associations with parental obesity	Partial correlation (23y)	NA	father-son	0.21	4924
				father-daughter	0.17	4943
				mother-son	0.24	5030
				mother-daughter	0.25	5087
Lake et al.[5] 1997 ¤	Child to adult body mass index in the 1958 British birth cohort associations with parental obesity	Partial correlation (33y)	NA	father-son	0.20	4403
				father-daughter	0.15	4491
				mother-son	0.21	4496
				mother-daughter	0.24	4644
Williams et al.[9] 2001	Overweight at age 21 the association with body mass index in childhood and adolescence and parents' body mass index. A	NA	NA	father-son	0.30	482
				mother-son	0.24	482
				mother-daughter	0.23	442
				father-daughter	0.23	442

Study (Author, year)	Title	Methods	Adjusted covariates	Family relationship	r	n
	cohort study of New Zealanders born in 1972–1973					
Magnusson et al.[11] 2002	Familial resemblance of body mass index and familial risk of high and low body mass index. A study of young men in Sweden	Pearson correlation	NA	father-son*	0.280	22517
				Quasi father-son**	0.060	1576
Salces et al.[12] 2002	Family resemblance for anthropometric traits II. Assessment of maternal occupational and age effects	NA	NA	father-offsping^	0.601	26
				mother-offspring^	0.241	54
				father-offsping^^	0.029	9
				mother-offspring^^	0.142	24
Mirmiran et al.[13] 2002	Familial clustering of obesity and the role of nutrition: Tehran Lipid and Glucose Study	Bivariate familial correlation	NA	father-son	0.19	27
				mother-son	0.06	27
				mother-daughter	0.29	45
				father-daughter	0.31	45
Wu et al.[15] 2003	Familial resemblance of adiposity-related parameters Results from a health check-up population in Taiwan	Pearson correlation	NA	father-son	0.19	431
				mother-son	0.15	431
				mother-daughter	0.14	431
				father-daughter	0.12	431
		Partial correlation	age	father-son	0.22	431
				mother-son	0.14	431
				mother-daughter	0.13	431
				father-daughter	0.15	431
Magarey et al.[16] 2003	Predicting obesity in early adulthood from childhood and parental obesity	Pearson's correlation	NA	father-son	0.20	86
				mother-son	0.35	96
				mother-daughter	0.32	87

Study (Author, year)	Title	Methods	Adjusted covariates	Family relationship	r	n
				father-daughter	0.51	70
Classen et al.[25] 2010	Measures of the intergenerational transmission of body mass index between mothers and their children in the United States, 1981–2004	correlation	NA	mother-offspring	0.35	4748
				mother-daughter	0.379	2348
				mother-son	0.319	2400
Cooper et al.[26] 2010	Associations between parental and offspring adiposity up to midlife the contribution of adult lifestyle factors in the 1958 British Birth Cohort Study	correlation	NA	father-son	0.21	3470
				father-daughter	0.14	3503
				mother-son	0.21	3537
				mother-daughter	0.23	3620
Reynold et al[27]. 2010	Maternal BMI, parity, and pregnancy weight gain: influences on offspring adiposity in young adulthood	Pearson's correlation	NA	mother-offspring	0.21	276
			age, sex, smoking status, social class, and activity level	mother-offspring	0.35	276
Murrin et al.[31] 2012	Body mass index and height over three generations evidence from the Lifeways cross-generational cohort study	Univariate correlation	NA	father-son	-0.059	25
				mother-son	0.085	42
				mother-daughter	0.269	128
				father-daughter	0.302	171
Kelly et al.[34] 2014	Body mass index is associated with the maternal lines but height is heritable across family lines in the Lifeways Cross-Generation Cohort Study	Mixed model	age	father-son	0.072	196
				mother-son	0.255	125
				mother-daughter	0.245	321
				father-daughter	0.070	201
Swanton et al.[44] 2017	Body mass index associations between mother and offspring from birth to age 18: the Fels Longitudinal Study	Spearman correlation	NA	mother-son	0.264	74
				mother-daughter	0.269	66

Study (Author, year)	Title	Methods	Adjusted covariates	Family relationship	r	n
**Sensitivity analyses or narrative**						
Cho et al.[36] 2015	Comparisons of chewing rhythm, craniomandibular morphology, body mass and height between mothers and their biological daughters	Pearson's correlation	NA	mother-daughter	0.340	32
Sørensen et al.[3] 1992	Correlations of body mass index of adult adoptees and their biological and adoptive relatives	correlation	NA	Adoptee-biological mother	0.15	540
				Adoptee-biological father	0.11	540
				Adoptee-biological sibling	0.23	540
Kazumi et al.[17] 2005	Associations of middle-aged mother's but not father's body mass index with 18-year-old son's waist circumferences, birth weight, and serum hepatic enzyme levels	Partial correlation	birth weight, son's BMI	father-son	-0.08	139
				mother-son	0.37	136

NA, not available; r, correlation coefficient
¤only 33 years old was included in the meta-analyses
^Housewife mothers, ^^working mothers

Table S3. Descriptions of studies reporting mean difference (MD) or standardized mean difference (SMD)

Study	Title	Methods	Adjusted Covariates	Exposure unit	Family relationship	MD or SMD	95% LL	95%UL	n
Burke et al.[7] 2001	Family lifestyle and parental body mass index as predictors of body mass index in Australian children: a longitudinal study	Multivariate regression	'unsafe' drinking, physical fitness, fat consumption, smoking in offspring, and parental education	kg/m^2	father-son	0.23	0.01	0.45	104
					father-daughter	0.44	0.24	0.64	115
					mother-son	0.17	0.04	0.29	104
					mother-daughter	0.24	0.11	0.37	115
Laitinen et al.[10] 2001	Family social class, maternal body mass index, childhood body mass index, and age at menarche as predictors of adult obesity	Linear regression	maternal age; social class	kg/m^2	mother-son	0.21	0.16	0.26	2876
					mother-daughter	0.37	0.32	0.42	3404
Kivimäki et al.[19] 2007	Substantial intergenerational increases in body mass index are not explained by the fetal overnutrition hypothesis: the Cardiovascular Risk in Young Finns Study	Linear regression	age, sex, maternal age, paternal age, maternal BMI, paternal BMI adjusted simultaneously	kg/m^2	mother-offspring	0.31	0.26	0.36	1918
					father-offspring	0.29	0.23	0.35	1918
			age, sex	kg/m^2	mother-offspring	0.32	0.27	0.37	1918
					father-offspring	0.34	0.28	0.41	1918
Abu-Rmeileh NM et al.[20] 2008	Contribution of Midparental BM I and other determinants of ob esity in adult offspring.	Linear regression	sex, age, social class, smoking habit, physical activity, and reported dietary intake	kg/m^2	mid-parental BMI with offspring BMI	0.51	0.41	0.62	2162
Koupil et al.[21] 2008	Social and early-life determinants of overweight and obesity in 18-year-old Swedish men	Linear regression	age, mother'age, parity, mother's education, smoking	kg/m^2	mother-son	0.39	0.30	0.47	1103
Tequeanes et al.[22] 2009	Maternal Anthropometry Is Associated with the Body Mass Index and Waist: Height Ratio of Offspring at 23 Years of Age	Multiple linear regressions	Unadjusted model	kg/m^2	mother-son	0.70	0.47	0.94	1076
					mother-daughter	0.94	0.66	1.21	1112
			maternal age, maternal smoking, maternal parity,	kg/m^2	mother-son	0.77	0.53	1.01	1076
					mother-daughter	0.94	0.66	1.22	1112

Study	Title	Methods	Adjusted Covariates	Exposure unit	Family relationship	MD or SMD	95% LL	95%UL	n
			maternal education, family income, and skin color						
Cooper et al.[26] 2010	Associations between parental and offspring adiposity up to midlife: the contribution of adult lifestyle factors in the 1958 British Birth Cohort Study	Multiple linear regression	parental age, lifestyle factors, and markers of socioeconomic position	kg/m^2	father-son	0.27	0.23	0.31	4651
					father-daughter	0.22	0.17	0.28	4695
					mother-son	0.21	0.18	0.25	4651
					mother-daughter	0.29	0.25	0.34	4695
Hochner et al.[29] 2012	Associations of Maternal Prepregnancy Body Mass Index and Gestational Weight Gain With Adult Offspring Cardiometabolic Risk Factors	Linear regression	ethnicity, sex, maternal and offspring characteristics at the time of birth (ie, parity, mother's age, maternal smoking, socioeconomic status, mother's years of education, maternal medical condition, birth weight, and gestational week) and offspring characteristics at 32 years of age	kg/m^2	mother-offspring*	0.48	0.38	0.59	1248
Han et al.[38] 2015	Contributions of maternal and paternal adiposity and smoking to adult offspring adiposity and cardiovascular risk: the Midspan Family Study	Linear Mixed effect models	family clustering, parental and offspring age, smoking and social class	kg/m^2	father-son	0.35	0.27	0.42	1025
					father-daughter	0.29	0.21	0.38	1283
					mother-son	0.26	0.20	0.32	1025
					mother-daughter	0.33	0.27	0.40	1283
			Mutually adjust for parental BMI	kg/m^2	father-son	0.30	0.23	0.38	1025
					father-daughter	0.23	0.15	0.32	1283
					mother-son	0.22	0.16	0.28	1025
					mother-daughter	0.33	0.24	0.37	1283
			Unadjusted model	kg/m^2	father-offspring	0.36	0.30	0.42	2229

Study	Title	Methods	Adjusted Covariates	Exposure unit	Family relationship	MD or SMD	95% LL	95%UL	n
Zalbahar et al.[41] 2016	Parental pre-pregnancy BMI influences on offspring BMI and waist circumference at 21 years	Multiple linear regression			mother-offspring	0.35	0.31	0.40	2229
			Other parent's BMI, offspring sex, maternal factor, maternal and paternal education, annual family income (model2)	kg/m^2	father-offspring	0.36	0.29	0.42	2229
					mother-offspring	0.38	0.32	0.43	2229
			Unadjusted model	kg/m^2	father-son	0.30	0.23	0.38	1114
					father-daughter	0.41	0.32	0.50	1115
					mother-son	0.25	0.19	0.32	1114
					mother-daughter	0.47	0.39	0.54	1115
			Other parent's BMI, offspring sex, maternal factor, maternal and paternal education, annual family income (model2)	kg/m^2	father-son	0.31	0.23	0.39	1114
					father-daughter	0.39	0.29	0.49	1115
					mother-son	0.30	0.22	0.37	1114
					mother-daughter	0.44	0.35	0.52	1115
Rodrigo M. Carrillo-Larco et al.[45] 2018	Parental body mass index and blood pressure are associated with higher body mass index and blood pressure in their adult offspring: a cross-sectional study in a resource-limited setting in northern Peru	Mixed-effects linear regression	Adjusted by village, age, educational level, physical activity of the offspring and wealth index of the family.	kg/m^2	father-son	0.26	0.14	0.38	253
					father-daughter	0.25	0.13	0.37	185
					mother-son	0.20	0.10	0.31	253
					mother-daughter	0.11	−0.00	0.22	185
			Unadjusted model	kg/m^2	father-son	0.20	0.08	0.32	253
					father-daughter	0.19	0.07	0.32	185
					mother-son	0.20	0.10	0.30	253
					mother-daughter	0.10	-0.02	0.21	185
Kvaavik et al.[14] 2003	Predictors and Tracking of Body Mass Index From Adolescence Into Adulthood	Linear regression	father's education, leisure time physical activity, fitness, smoking, baseline BMI, sex, own education, leisure	SD	mother-offspring	0.07	-0.01	0.16	233
					father-offsping	0.12	0.03	0.21	249

Study	Title	Methods	Adjusted Covariates	Exposure unit	Family relationship	MD or SMD	95% LL	95%UL	n
			time physical activity, smoking at FU						
Hu et al.[33] 2008	Familial correlation and aggregation of body mass index and blood pressure in Chinese Han population	Multilevel linear regression	children's age, both childrens' and parents' education level and occupation	SD	father-son	0.27	0.23	0.30	4132
					mother-son	0.25	0.21	0.27	4132
					father-daughter	0.23	0.18	0.27	2237
					mother-daughter	0.21	0.18	0.23	2237
Johnson et al.[30] 2012	Intergenerational change and familial aggregation of body mass index	Multilevel linear regression	Age, marital status, number of children, smoking status and social class	SD	father-son	0.27	0.22	0.33	1023
					father-daughter	0.19	0.14	0.25	1263
					mother-son	0.28	0.22	0.33	1023
					mother-daughter	0.31	0.27	0.36	1263
					mother-offspring	0.30	0.26	0.34	1443
					father-offspring	0.23	0.19	0.27	1443
Swanton et al.[44] 2017	Body mass index associations between mother and offspring from birth to age 18: the Fels Longitudinal Study	Multiple linear regression	Maternal birth year	SD	mother-son	0.27	-0.00	0.54	74
					mother-daughter	0.46	-0.00	0.92	66
			Decade of birth, parity	SD	mother-son	0.28	-0.00	0.56	156
					mother-daughter	0.30	-0.00	0.60	133
Kaseva et al.[46] 2018 ø	Pre-pregnancy overweight or obesity and gestational diabetes as predictors of body composition in offspring twenty years later: evidence from two birth cohort studies	Linear regression	age, cohort, gestational age, birth weight SD score, maternal hypertension or preeclampsia during pregnancy, maternal smoking during pregnancy and parental educational attainment	OWOB Versus Normal	mother-son	1.64	0.57	2.72	335
					mother-daughter	1.41	0.20	2.63	365
			Age, cohort	OWOB vs normal	mother-son	2.35	1.34	3.36	335
					mother-daughter	1.67	0.56	2.78	365
Vik et al.[35] 2014 ø	Comparison of father-offspring and mother-offspring associations of cardiovascular	Linear regression	Age, sex	kg/m^2	father-offspring	*0.22*	*0.20*	*0.23*	36528

Study	Title	Methods	Adjusted Covariates	Exposure unit	Family relationship	MD or SMD	95% LL	95%UL	n
	risk factors family linkage within the population-based HUNT Study, Norway				mother-offspring	0.17	0.16	0.18	36528
				SD	father-offspring	*0.19*	*0.18*	*0.20*	36528
					mother-offspring	0.20	0.18	0.21	36528
Eriksson et al.[39] 2015 [Ø]	Maternal weight in pregnancy and offspring body composition in late adulthood: findings from the Helsinki Birth Cohort Study (HBCS)	Multiple linear regression	Age and sex	kg/m^2	Mother-offspring	4.9	2.7	7.0	1650
			Age		Mother-son	6.9	4.0	9.8	809
					Mother-daughter	2.6	-0.6	5.8	809
Chaparro et al.[48] 2017 [Ø]	Maternal pre-pregnancy BMI and offspring body composition in young adulthood: the modifying role of offspring sex and birth order	Multivariable linear regression	Age, height, mother's age at birth, and mother's education	SD	mother-son	0.59	0.27-1.44	226	226
					mother-daughter	0.97	0.14-1.80	226	226
Schoppa et al.[47] 2019 [Ø]	Association of Maternal Prepregnancy Weight with Offspring Adiposity Throughout Adulthood over 37 Years of Follow-up	Linear regression	Age, sex	OWOB vs normal	mother-offspring	1.4	0.62	2.18	863
			Age, sex, BMI GRS	OWOB vs normal	mother-offspring	1.6	0.62	2.58	766

LL, lower limit; UL, upper limit; MD, mean difference; SMD, standardized mean difference; OWOB, overweight or obese
[Ø] not included in meta-analyses

Table S4. Descriptions of studies reporting odds ratios (ORs) or risk ratios (RRs)

Author	Title	Exposure Criteria	Outcome Criteria	Family relationship	Comparison group vs reference group	ORs (95%CI)			Adjustment
						Over-weight	Obesity	OWOB	
Lake et al.[5] 1997	Child to adult body mass index in the 1958 British birth cohort associations with parental obesity	Underweight (<20 kg/m^2 for father, <18.7 for mother); Normal weight(20-24.9 kg/m^2 for father, 18.7-23.7 for mother); Overweight(25-27.7 kg/m^2 for father, 23.7-27.6 for mother); Obesity(>27.7 kg/m^2 for father, >27.6 for mother)	at or above 85th percentile for their age and sex are classified as obesity	parent-son	Both parents with overweight vs both parents with normal weight		3.41 (2.36-4.93)		Age
				parent-son	One parent with obesity vs both parents with normal weight		3.43 (2.53-4.64)		
				parent-daughter	One parent with overweight vs both parents with normal weight		1.47 (1.14-1.88)		
				parent-son	Both parents with obesity vs both parents with normal weight		8.42 (5.47-13.00)		
				parent-daughter	Both parents with obesity vs both parents with normal weight		6.75 (4.57-9.96)		
				parent-son	One parent with overweight both parents with normal weight		1.99 (1.45-2.73)		
				parent-daughter	Both parents with overweight vs both parents with normal weight		2.65 (1.95-3.62)		
				parent-daughter	One parent with obesity vs both parents with normal weight		2.98 (2.35-3.78)		

Author	Title	Exposure Criteria	Outcome Criteria	Family relationship	Comparison group vs reference group	ORs (95%CI)			Adjustment
						Over-weight	Obesity	OWOB	
Al-Isa et al.[6] 1998	Factors Associated with Overweight and Obesity among Kuwaiti College Women	both parents obese; father or mother obese; neither parent obese	Normal weight<25; 25⩽overweight<30; obesity>30 kg/m^2	mother-daughter	Mother with obesity vs both parents with normal weight	2.02 (1.28-3.19)	4.27 (2.02-9.05)		number of brothers, having chronic diseases, Having Obesity parent, Dieting, Country prefer visiting
				father-daughter	Father with obesity vs both parents with normal weight	1.46 (0.67-3.20)	1.31 (0.27-6.34)		
Burke et al.[7] 2001	Family lifestyle and parental body mass index as predictors of body mass index in Australian children: a longitudinal stud	Normal weight<25; 25⩽overweight<30; obesity>30 kg/m^2	Normal weight<25; 25⩽overweight<30; obesity>30 kg/m^2	mother-daughter	mother with obesity vs mother with normal weight			7.88 (1.97-31.43)	'unsafe' drinking, physical fitness, fat consumption, smoking in offspring, and parental education
				father-daughter	father with obesity vs father with normal weight			3.83 (1.01-14.54)	
				mother-son	mother with obesity vs mother with normal weight			2.49 (0.69-8.89)	
				father-son	father with obesity vs father with normal weight			4.38 (1.16-16.48)	
Williams et al.[9] 2001	Overweight at age 21: the association with body mass index in childhood and adolescence and parents' body mass index. A cohort study	underweight<18.5; 18.5⩽normal weight<25; 25⩽overweight<30; obesity⩾30 kg/m^2	underweight<18.5; 18.5⩽normal weight<25; 25⩽overweight<30; obesity⩾30 kg/m^2	father-offspring	father with overweight vs father with normal weight			1.2 (0.60-2.00)	Sex, and other parent's BMI, children's BMI at 11 years
				mother-offspring	mother with obesity vs mother with normal weight			1.9 (1.20-2.50)	
				father-offspring	father with obesity vs father with normal weight			1.3 (1.00-1.70)	
				mother-offspring	mother with overweight vs			1.1 (0.80-1.60)	

Author	Title	Exposure Criteria	Outcome Criteria	Family relationship	Comparison group vs reference group	ORs (95%CI)			Adjustment
						Over-weight	Obesity	OWOB	
	of New Zealanders born in 1972–1973				mother with normal weight				
Kvaavik et al.[14] 2003	Predictors and Tracking of Body Mass Index From Adolescence Into Adulthood	Normal weight<25; 25⩽overweight<30; obesity>30 kg/m^2	Normal weight<25; 25⩽overweight<30; obesity>30 kg/m^2	parent-offspring	Either parent with overweight vs both parents with normal weight	0.54(0.89)	0.61(1.6)		Adolescent BMI, Tanner stage, sex,the adolescents' ascribed and family characteristics, perceptions of family relationship,adolescents' self-esteem and weight status adolescent behaviors
Crossman et al.[18] 2006	The family environment and American adolescents' risk of obesity as young adults	not obese: <30, obese⩾30 kg/m^2	CDC's age- and sex-specific BMI percentiles for children and adolescents	mother-son	mother with obesity vs mother with normal weight			1.33	the adolescents' ascribed and family characteristics
				father-daughter	father with obesity vs father with normal weight			1.58	
				mother-daughter	mother with obesity vs mother with normal weight			1.39	
				father-son	father with obesity vs father with normal weight			1.6	
Al-Isa et al.[28] 2013	Factors Associated With Overweight	Normal weight⩽25; 25<overweig	Normal weight⩽25; 25<overweight<3	parent-son	Both parents with obesity vs both parents with normal weight	5.9 (1.4-25.7)	2.9 (1.14-7.19)		Offspring age, dental status, chronic disease, number of

Author	Title	Exposure Criteria	Outcome Criteria	Family relationship	Comparison group vs reference group	ORs (95%CI)			Adjustment
						Over-weight	Obesity	OWOB	
	and Obesity Among Kuwaiti Men	ht<30; obesity>30 kg/m^2	0; obesity>30 kg/m^2	mother-son	mother with obesity vs both parents with normal weight	2 (1.19-3.68)	2.4 (1.24-4.74)		Obesity brothers, number of Obesity relatives, wife's education, last GPA, high school GPA, monthly family income, physical activity, sport, health status, dieting, feeling tired
				father-son	father with obesity vs both parents with normal weight	1.9 (0.84-4.4)	1.3 (0.53-3.2)		
Wang et al.[32] 2012	Epidemiology of general obesity, abdominal obesity and related risk factors in urban adults from 33 communities of northeast china: the CHPSNE study	history of obesity	Normal weight<25; 25⩽overweight<27.5; obesity⩾27.5 kg/m^2	parent-offspring	One parent with obesity vs both parents with normal weight		2.61 (2.4-2.83)		age, ethnicity, education, occupation, family income, physical activity, cigarette smoking, alcohol, eat fried foods, diet
				parent-son	One parent with obesity vs both parents with normal weight		3.51 (3.13-3.94)		
				parent-daughter	One parent with obesity vs both parents with normal weight		2.06 (1.82-2.33)		
Magarey et al.[49] 2003	Predicting obesity in early adulthood from childhood	Normal weight<25; 25⩽overweight<30; obesity>30 kg/m^2	Normal weight<25; 25⩽overweight<30; obesity>30 kg/m^2	parent-offspring	One parent with OWOB vs parent with normal weight			2.1 (1.0–4.5)	NA
				parent-offspring	Two parents with OWOB vs parent with normal weight			4.3 (2.1–8.9)	

Author	Title	Exposure Criteria	Outcome Criteria	Family relationship	Comparison group vs reference group	ORs (95%CI)			Adjustment
						Over-weight	Obesity	OWOB	
	and parental obesity								
Derraik et al.[37] 2015	Obesity rates in two generations of Swedish women entering pregnancy and associated obesity risk among adult daughters	underweight <18.5; 18.5⩽normal weight<25; 25⩽overweight<30; obesity⩾30 kg/m^2	underweight<18.5; 18.5⩽normal weight<25; 25⩽overweight<30; obesity⩾30 kg/m^2	mother-daughter	mother with overweight vs mother with normal weight	2.44 (2.26, 2.63)			Unadjusted
				mother-daughter	mother with obesity vs mother with normal weight	3.71 (3.18, 4.34)			
				mother-daughter	mother with overweight vs mother with normal weight		2.71 (2.46, 2.98)		
				mother-daughter	mother with obesity vs mother with normal weight		4.65 (3.95, 5.48)		
Han et al.[38] 2015	Contributions of maternal and paternal adiposity and smoking to adult offspring adiposity and cardiovascular risk: the Midspan Family Study	Normal weight<25; 25⩽overweight<30; obesity>30 kg/m^2	Normal weight<25; 25⩽overweight<30; obesity>30 kg/m^2	parent-offspring	parent with obesity vs both parents with normal weight	10.4 (4.65-13.29)			family clustering, offspring and parental age, smoking status (either current or former smokers) and social class
				parent-offspring	parent with obesity vs both parents with normal weight	1.96 (1.54-2.48)			
				parent-offspring	parent with obesity vs both parents with normal weight		11.77 (7.70-18.00)		
				parent-offspring	parent with obesity vs both parents with normal weight		2.14 (1.62-2.81)		
Alati et al.[40] 2016	Generational increase in obesity	underweight <18.5; 18.5⩽normal	underweight<18.5; 18.5⩽normal weight<25;	mother-daughter	mother with overweight vs	2.54 (1.86, 3.54)			age in years, education, number of

Author	Title	Exposure Criteria	Outcome Criteria	Family relationship	Comparison group vs reference group	ORs (95%CI)			Adjustment
						Over-weight	Obesity	OWOB	
	among young women: a prospective analysis of mother–daughter dyads	weight<25; 25⩽overweight<30; obesity⩾30 kg/m^2	25⩽overweight<30; obesity⩾30 kg/m^2		mother with normal weight				previous pregnancies, exercise and television viewing
				father-son	father with overweight vs father with normal weight		7.85 (3.58-22.83)		
				father-daughter	father with overweight vs father with normal weight	1.52 (1.16-2.08)			
				father-son	father with overweight vs father with normal weight	1.91 (1.29-2.93)			
				mother-son	mother with obesity vs mother with normal weight		4.01 (2.36-7.46)		
				mother-son	mother with overweight vs mother with normal weight	2.83 (2.03-4.13)			
				mother-daughter	mother with obesity vs mother with normal weight		5.04 (3.03, 8.85)		
				father-daughter	father with obesity vs father with normal weight		8.33 (5.10-15.96)		
Zalbahar et al.[41] 2016	Parental pre-pregnancy BMI	underweight <18.5; 18.5⩽normal	underweight<18.5; 18.5⩽normal weight<25;	father-offspring	father with OWOB vs father with normal weight			2.39 (1.82-3.14)	other parent’s, offspring’s sex, gestational

Author	Title	Exposure Criteria	Outcome Criteria	Family relationship	Comparison group vs reference group	ORs (95%CI)			Adjustment
						Over-weight	Obesity	OWOB	
	influences on offspring BMI and waist circumference at 21 years	weight<25; 25⩽overweight<30; obesity⩾30 kg/m^2	25⩽overweight<30; obesity⩾30 kg/m^2	mother-offspring	Mother with OWOB vs mother with normal weight			2.36 (1.69-3.29)	weight gain, maternal age at birth, maternal smoking during pregnancy), maternal and paternal education attainment, annual family income
				parent-offspring	both parents with OWOB vs parent with normal weight			5.73 (3.34-9.83)	
Rath et al.[42] 2016	Parental pre-pregnancy BMI is a dominant early-life risk factor	Normal weight<25; 25⩽overweight<30; obesity>30 kg/m^2	at or above 95th percentile for their age and sex are classified as obesity	father-offspring	father with overweight vs father with normal weight		1.44 (0.92-2.23)		Sex, maternal education, maternal smoking, maternal anemia and diabetes, cesarean, prematurity, birth weight, first year weight gain, duration of breast-feeding
				mother-offspring	mother with obesity vs mother with normal weight		6.96 (3.81-12.52)		
				mother-offspring	mother with overweight vs mother with normal weight		4.58 (2.87-7.24)		
				father-offspring	father with obesity vs father with normal weight		3.75 (1.80-7.50)		
Stuebe et al.[24] 2009	Maternal-recalled gestational weight gain, pre-pregnancy body mass index, and obesity in the daughter	BMI cut off: 21, 23, 25, 29 kg/m^2	Normal weight<25; 25⩽overweight<30; obesity>30 kg/m^2	mother-daughter	mother BMI=29 vs BMI =21 kg/m^2		7.53 (6.00-9.45)		Unadjusted (when child aged 18y)

Author	Title	Exposure Criteria	Outcome Criteria	Family relationship	Comparison group vs reference group	ORs (95%CI)			Adjustment
						Over-weight	Obesity	OWOB	
Koupil et al.[21] 2008	Social and early-life determinants of overweight and obesity in 18-year-old Swedish men	continuous	Normal weight<25; 25⩽overweight<30; obesity>30 kg/m^2	mother-son	per 1 kg/m^2 increase in mother's mean BMI			1.23 (1.16-1.30)	age, mother's age, parity and mother's education, smoking
Kowalski-Jones et al.[50] 2009	Are You What Your Mother Weighs? Evaluating the Impact of Maternal Weight Trajectories on Youth Overweight	continuous	at or above 95th percentile for their age and sex are classified as overweight	mother-son	per 1 kg/m^2 increase in mother's mean BMI	1.11(1.07–1.16)			family income, marriage time, residence, maternal weeks worked, neighborhood quality
				mother-daughter	per 1 kg/m^2 increase in mother's mean BMI	1.06(1.07–1.15)			
Reynolds et al.[27] 2010	Maternal BMI, Parity, and Pregnancy Weight Gain: Influences on Offspring Adiposity in Young Adulthood	continuous	OWOB vs normal	mother-offspring	per 1 SD increase in mother's mean BMI			1.99	Gender, smoking, maternal age, antenatal weight gain, primiparous

Note: all studies used logistic regression, except Han et al.[38], which used generalized estimating equation model.
BMI, body mass index; OR, odds ratio; CI, confidence interval; OWOB, overweight or obese; SD, standard deviation

Table S5. Pooled standardized mean difference between parental and offspring BMI (per standard deviation)

Series	Family relationship	Pooled SMD for adjusted models				Pooled SMD for unadjusted models*			
		SMD	95%CI	I^2 (%)	Study number	SMD	95%CI	I^2 (%)	Study number
**Level1**	Mother-daughter	0.25	0.21-0.29	70.5	10	0.25	0.20-0.30	81.4	17
	Mother-son	0.23	0.20-0.26	61.8	11	0.23	0.20-0.27	56.0	16
	Father-daughter	0.21	0.16-0.26	68.5	7	0.20	0.16-0.24	67.1	12
	Father-son	0.25	0.21-0.28	39.9	7	0.23	0.19-0.27	77.7	13
**Level2**	Mother-offspring	0.23	0.20-0.26	78.9	13	0.24	0.21-0.28	84.6	18
	Father-offspring	0.22	0.19-0.25	68.1	9	0.21	0.18-0.25	71.5	13

SMD, standardized mean difference; SD, standardized deviation; CI, Confidence interval

*include studies reporting correlation coefficient and unadjusted SMD in regression coefficient

I^2 = proportion of total variation in effect estimate due to between-study heterogeneity (based on Q)

Table S6. Pooled mean difference (MD) between parental and offspring BMI (per kg/m^2)

**Series**	**Family relationship**	**Pooled MD for adjusted models**				**Pooled MD for unadjusted models**			
		**MD**	**95%CI**	**I² (%)**	**Study number**	**MD**	**95%CI**	**I² (%)**	**Study number**
**Level1**	Mother-daughter	0.34	0.26-0.43	87.6	7	0.48	0.13-0.83	95.5	3
	Mother-son	0.27	0.21-0.34	82.4	8	0.34	0.16-0.52	86.6	3
	Father-daughter	0.30	0.22-0.38	67.7	5	0.30	0.09-0.52	86.7	2
	Father-son	0.29	0.26-0.32	3.1	5	0.26	0.17-0.36	47.9	2
**Level2**	Mother-offspring	0.34	0.28-0.41	90.7	9	0.38	0.24-0.51	93.7	4
	Father-offspring	0.30	0.26-0.35	65.0	6	0.30	0.21-0.39	80.5	3

MD, mean difference; CI, Confidence interval

I^2 = proportion of total variation in effect estimate due to between-study heterogeneity (based on Q)

Table S7. Difference of standardized mean difference between maternal and paternal line in adjusted models

Author	Diff. Of SMD	95% CI		% Weight	Test of overall effect		
		LL	UL		p value compare MO and FO	I^2 (%)	tau^2
Burke	-0.02	-0.19	0.16	3.02	0.49	46.8	0.0011
Cooper	0.01	-0.03	0.05	18.42			
Han	0.03	-0.04	0.10	11.51			
Hu	-0.03	-0.06	0.01	21.15			
Johnson	0.07	0.01	0.13	14.7			
Kivimäki	0.06	-0.02	0.14	10.34			
Kvaavik	-0.05	-0.17	0.07	5.32			
Carrillo-Larco	-0.09	-0.21	0.02	6.08			
Zalbahar	0.04	-0.05	0.12	9.46			
Overall, DL	0.01	-0.02	0.05	100			

SMD, Standardized Mean Difference; MO, mother-offspring; FO, father-offspring; LL, lower limit; UL, upper limit

I^2 = proportion of total variation in effect estimate due to between-study heterogeneity (based on Q)

Table S8. Difference of standardized mean difference between maternal and paternal line in unadjusted models

Author	Diff. in SMD	95% CI			Test of overall effect		
		LL	UL	% Weight	p value compare MO and FO	I² (%)	tau²
Cooper	0.05	0.01	0.08	19.75	0.82	65.5	0.003
Friedlander	-0.06	-0.11	-0.01	17.52			
Kelly	0.18	0.05	0.32	7.43			
Khoury	-0.08	-0.41	0.24	1.82			
Lake	0.05	0.02	0.08	20.21			
Magarey	-0.01	-0.23	0.20	3.66			
Mirmiran	-0.06	-0.40	0.28	1.65			
Murrin	-0.04	-0.25	0.17	3.92			
Salces	-0.34	-0.38	-0.30	1.07			
William	-0.03	-0.13	0.06	11.68			
Wu	-0.05	-0.15	0.04	11.29			
Overall, DL	0.01	-0.04	0.05	100			

SMD, Standardized Mean Difference; MD, mean difference; MO, mother-offspring; FO, father-offspring; LL, lower limit; UL, upper limit

I^2 = proportion of total variation in effect estimate due to between-study heterogeneity (based on Q)

Table S9. Difference of mean difference between maternal and paternal line

Series	Author	Difference of MDs	95% CI		% Weight	Test of overall effect		
			LL	UL		p value compare MO and FO	I² (%)	tau²
i.MD adjusted covariates	Burke	-0.14	-0.32	0.03	3.66	0.29	14.1	0.0003
	Cooper	-0.01	-0.06	0.03	40.18			
	Han	-0.03	-0.10	0.04	18.49			
	Kivimäki	0.02	-0.06	0.10	15.9			
	Carrillo-Larco	-0.10	-0.21	0.02	8.08			
	Zalbahar	0.02	-0.07	0.11	13.69			
	Overall, DL	-0.02	-0.05	0.02	100			
ii.MD without adjusting for covariates	Kivimäki	-0.02	-0.10	0.06	39.26	0.45	0.0	0.0000
	Rodrigo M. Carrillo-Larco	-0.04	-0.15	0.08	18.16			
	Zalbahar	-0.01	-0.09	0.07	44.19			
	Overall, DL	-0.02	-0.07	0.03	100			

MD, mean difference; CI, Confidence interval; MO, mother-offspring; FO, father-offspring; LL, lower limit; UL, upper limit

I^2 = proportion of total variation in effect estimate due to between-study heterogeneity (based on Q)

Table S10. Difference of mean difference between maternal and paternal line (restricting to studies assessing parental BMI when children were young)

Series	Author	Difference of MDs	95% CI		% Weight	Test of overall effect		
			LL	UL		p value compare MO and FO	I² (%)	tau²
SMD adjusted covariates	Burke	-0.02	-0.19	0.16	3.52	0.499	0.0	0.0000
	Cooper	0.01	-0.03	0.05	57.97			
	Kivimäki	0.06	-0.02	0.14	17.20			
	Kvaavk	-0.05	-0.17	0.07	6.85			
	Zalbahar	0.06	-0.03	0.14	14.47			
	Overall, DL	0.02	-0.01	0.05	100			

MD, mean difference; CI, Confidence interval; MO, mother-offspring; FO, father-offspring; LL, lower limit; UL, upper limit

I^2 = proportion of total variation in effect estimate due to between-study heterogeneity (based on Q)

Table S11. Summary of studies not included in meta-analyses

Study	Title	Parental BMI categories	Offspring outcome	Main finding	Reason not included
Sørensen et al.[3] 1992	Correlations of body mass index of adult adoptees and their biological and adoptive relatives	BMI continuous	BMI continuous	The study found correlations between adoptees and their biological parents, but no correlation in BMI between the adoptees and their adoptive parents	The adoption study setting is different from other studies
Vik et al.[35] 2013	Comparison of father-offspring and mother-offspring associations of cardiovascular risk factors family linkage within the population-based HUNT Study, Norway	Parental BMI as continuous	Offspring BMI continuous	This study found similar father-offspring and mother-offspring BMI associations across all cardiovascular risk factors under study	The study reported residuals from linear regression analysis, and therefore not amenable to inclusion in the meta-analysis
Eriksson et al.[39] 2015	Maternal weight in pregnancy and offspring body composition in late adulthood: findings from the Helsinki Birth Cohort Study (HBCS)	Maternal BMI >28.1 kg/m^2 vs Maternal BMI <28.1 kg/m^2	% fat mass % lean mass	Higher maternal BMI was associated with less favorable body composition in the offspring	Outcome is body fat percentage
Chaparro et al.[48] 2017	Maternal pre-pregnancy BMI and offspring body composition in young adulthood: the modifying role of offspring sex and birth order	Maternal pre-pregnancy BMI	% fat mass % lean mass	A higher pre-pregnancy BMI was associated with higher offspring % fat mass and lower offspring % lean mass in late adolescence and young adulthood	Outcome is body fat percentage
Kaseva et al.[46] 2018	Pre-pregnancy overweight or obesity and gestational diabetes as predictors of body composition in offspring twenty years later: evidence from two birth cohort studies	normoglycemic mothers who were OWOB, GDM group, and normal group	BMI continuous	Maternal unhealthy BMI before pregnancy was associated with body composition in offspring, including BMI, waist circumference, fat mass, and fat percentage	The study reported risk difference of offspring's BMI between these born with mothers who were OWOB versus normal weight groups
Schoppa et al.[47] 2018	Association of Maternal Prepregnancy Weight with Offspring Adiposity Throughout Adulthood over 37 Years of Follow-up	Mothers with OWOB versus mothers with normal weight	BMI continuous	Maternal unhealthy BMI before pregnancy was associated with greater offspring BMI throughout adulthood from almost 40 years of follow-up	The study reported risk difference of offspring's BMI between these born with mothers who were OWOB versus normal weight groups

Table S12. Subgroup analyses by BMI measurement method

Categories	Family relationship	Self-reported				Measured				Overall I² (%)
		SMD	95%CI	Study numbers	I² (%)	SMD	95%CI	Study numbers	I² (%)	
Offspring's BMI measurement methods	Mother-offspring	0.14	0.01-0.27	2	87.2	0.25	0.22-0.28	12	73.3	77.5
	Father-offspring	0.12	0.03-0.21	1	0.0	0.23	0.20-0.26	8	65.0	68.1
Parents'BMI measurement methods	Mother-offspring	0.21	0.15-0.27	6	79.7	0.26	0.23-0.30	7	66.5	77.5
	Father-offspring	0.17	0.13-0.22	3	42.7	0.25	0.23-0.27	6	0.0	68.1

SMD, standardized mean difference; CI, confidence interval; NA, not available

I² = proportion of total variation in effect estimate due to between-study heterogeneity (based on Q)

Table S13. Subgroup analyses by study design

Family relationship	Cohort studies				Cross-sectional studies				Overall I^2 (%)
	SMD	95%CI	Study numbers	I^2 (%)	SMD	95%CI	Study numbers	I^2 (%)	
Mother- daughter	0.26	0.20-0.32	6	71.8	0.24	0.18-0.30	4	65.3	70.5
Mother-son	0.22	0.17-0.26	7	65.3	0.25	0.23-0.28	4	0.00	61.80
Father-daughter	0.17	0.11-0.24	3	69.2	0.24	0.19-0.31	4	41.3	68.5
Father-son	0.23	0.18-0.28	3	58.3	0.27	0.24-0.30	4	0.00	39.9
Mother-offspring	0.23	0.19-0.28	10	82.2	0.24	0.21-0.28	4	53.8	77.50
Father-offspring	0.20	0.16-0.23	5	60.8	0.25	0.23-0.28	4	0.00	68.10

SMD, standardized mean difference; CI, confidence interval

I^2 = proportion of total variation in effect estimate due to between-study heterogeneity (based on Q)

Table S14. Subgroup analyses by maternal BMI measurement time point

Family relationship	Before-pregnancy				Post-pregnancy				Overall I^2 (%)
	SMD	95%CI	Study number	I^2 (%)	SMD	95%CI	Study number	I^2 (%)	
Mother-daughter	0.27	0.23-0.31	4	0.00	0.24	0.19-0.29	6	80.5	70.5
Mother-son	0.21	0.15-0.27	5	40.8	0.24	0.20-0.28	6	70.0	61.8
Mother-offspring	0.25	0.20-0.29	6	39.3	0.23	0.19-0.27	8	85.9	76.6

SMD, standardized mean difference; CI, confidence interval

I^2 = proportion of total variation in effect estimate due to between-study heterogeneity (based on Q)

Table S15. Subgroup analyses by offspring age

Family relationship	Early adulthood				Mid-adulthood				Late-adulthood				Overall I² (%)
	SMD	95%CI	Study number	I² (%)	SMD	95%CI	Study number	I² (%)	SMD	95%CI	Study number	I² (%)	
Mother-daughter	0.24	0.20-0.28	7	51.7	NA	NA	NA	NA	0.26	0.18-0.34	3	87.2	70.5%
Mother-son	0.23	0.19-0.26	8	41.0	NA	NA	NA	NA	0.24	0.16-0.31	3	85.4	61.8%
Father-daughter	0.25	0.21-0.30	4	16.3	NA	NA	NA	NA	0.16	0.11-0.21	3	51.8	68.5%
Father-son	0.26	0.23-0.29	4	0.00	NA	NA	NA	NA	0.25	0.18-0.31	3	72.5	39.9%
Mother-offspring	0.22	0.19-0.26	9	66.9	0.27	0.23-0.32	2	0.00	0.25	0.17-0.33	3	93.2	83.3%
Father-offspring	0.24	0.19-0.28	5	57.6	0.22	0.16-0.28	1	NA	0.21	0.16-0.26	3	76.4	40.2%

*Early adulthood: 18-30y; Mid-adulthood: 25-39y or 30-40y; Late adulthood: >40y

SMD, standardized mean difference; CI, confidence interval; NA, not available

I^2 = proportion of total variation in effect estimate due to between-study heterogeneity (based on Q)

Table S16. Subgroup analyses by parent-offspring BMI assessed time

Family relationship	Parallel young age				Parallel midlate age				Late aged parent-young adult child				Overall I² (%)
	SMD	95%CI	Study number	I² (%)	SMD	95%CI	Study number	I² (%)	SMD	95%CI	Study number	I² (%)	
Mother-daughter	0.27	0.23-0.31	4	0.0	0.26	0.18-0.34	3	87.2	0.22	0.14-0.0.30	3	60.9	70.5
Mother-son	0.21	0.15-0.27	5	40.8	0.24	0.16-0.31	3	85.4	0.25	0.22-0.28	3	0.0	61.8
Father-daughter	0.24	0.15-0.34	1	.	0.16	0.11-0.21	3	51.8	0.27	0.19-0.36	3	44.1	68.5
Father-son	0.22	0.14-0.31	1	.	0.25	0.18-0.31	3	72.5	0.26	0.23-0.30	3	0.0	39.9
Mother-offspring	0.24	0.19-0.29	5	45.9	0.23	0.16-0.29	5	91.4	0.23	0.19-0.27	3	37.3	78.9
Father-offspring	0.23	0.17-0.30	1	.	0.20	0.16-0.24	5	65.9	0.26	0.23-0.28	0	76.4	68.1

SMD, standardized mean difference; CI, confidence interval

I² = proportion of total variation in effect estimate due to between-study heterogeneity (based on Q)

Table S17. Sensitivity analyses-standardized mean difference*

Series	Family relationship	SMD	95%CI	I^2 (%)	Study number
**Level1**	Mother-daughter	0.25	0.20-0.30	81.8	10
	Mother-son	0.24	0.20-0.28	61.6	14
	Father-daughter	0.19	0.15-0.23	63.1	10
	Father-son	0.22	0.17-0.26	64.6	12
**Level2**	Mother-offspring	0.25	0.21-0.29	86.2	16
	Father-offspring	0.21	0.17-0.26	91.6	13

*unadjusted SMD, exclude studies with some participants younger than 18 years.

SMD, standardized mean difference; CI, confidence interval

I^2 = proportion of total variation in effect estimate due to between-study heterogeneity (based on Q)

Table S18. Sensitivity analysis- standardized mean difference taking out studies with low quality score*

Series	Family relationship	Pooled SMD for adjusted models				Pooled SMD for unadjusted models**			
		SMD	95%CI	I^2 (%)	Study number	SMD	95%CI	I^2 (%)	Study number
**Level1**	Mother-daughter	0.25	0.21-0.29	68.6	10	0.26	0.21-0.31	82.6	15
	Mother-son	0.23	0.20-0.26	61.8	11	0.24	0.21-0.27	59.6	16
	Father-daughter	0.21	0.16-0.26	67.9	7	0.20	0.16-0.25	70.0	11
	Father-son	0.25	0.21-0.28	38.5	7	0.22	0.18-0.26	82.6	13
**Level2**	Mother-offspring	0.24	0.22-0.27	74.7	12	0.25	0.22-0.29	85.5	17
	Father-offspring	0.23	0.20-0.26	65.0	8	0.22	0.18-0.25	73.7	12

SMD, standardized mean difference; SD, standardized deviation; CI, Confidence interval

*take out studies by Kvaavik et al.[14], Cho et al.[36], and Khoury et al.[1]

**include studies reporting correlation and unadjusted SMD in regression coefficient

I^2 = proportion of total variation in effect estimate due to between-study heterogeneity (based on Q)

Table S19. Study quality assessment using Adapted Newcastle-Ottawa scale

**Study, year**	**Selection**				**Comparability**	**Outcome**			**Stars**
	Representative-ness of the exposed cohort	Selection of the non-exposed cohort	Ascertainment of exposure	Demonstration that outcome of interest was not present at start of study	Comparability of cohorts on the basis of the design or analysis	Assessment of outcome	Was follow-up long enough for outcomes to occur	Adequacy of follow up of cohorts	
Khoury et al. [1] 1983,USA	---	★	---	---	---	---	★	---	2
Friedlanderet al.[2] 1988, Israel	★	★	★	---	★	★	★	---	6
Rotimi et al.[4] 1995,USA	★	★	★	★	★	★	★	---	7
Lake et al.[5] 1997,UK	★	★	★	★	★	★	★	★	8
Al-Isa et al.[6] 1998, Kuwaiti	---	★	---	---	★	★	★	★	5
Burke et al.[7] 2001, Australia	---	★	★	★	★	★	★	---	6
Williams et al.[9] 2001,New Zealand	★	★	---	★	★	★	★	★	7

Laitinen et a[10]<br>2001,Finland	★	★	★	★	★	★	★	★	8
Magnusson et al.[11]<br>2002,Sweden	★	★	---	★	★	---	★	★	6
Salces et al.[12]<br>2002, Spain	---	★	★	---	---	★	★	★	5
Mirmiran et al.[13]<br>2002, Iran	★	★	★	---	---	★	★	---	5
Kvaavik et al.[14]<br>2003, Norway	---	★	---	★	---	---	★	---	3
Wu et al.[15]<br>2003, Taiwan	---	★	★	★	★	★	★	---	6
Kazumi et al.[17]<br>2005, Japan	---	★	---	★	---	★	★	★	5
Crossman et al.[18]<br>2006, USA	★	★	---	★	★	★	★	---	6
Kivimäki et al.[19]<br>2007, Finland	★	★	★	★	★	★	★	---	7

Abu-Rmeileh, Hart et al. [20]<br><br>2008, Scotland	★	★	★	★	★	★	★	★	8
Koupil et al.[21],<br><br>2008, Sweden	★	★	★	★	★	★	★	★	8
Tequeanes et al.[22]<br><br>2009,Brazil	★	★	★	★	★	★	★	---	7
Kowaleski-Jones et al.[23]<br><br>2009,USA	★	★	---	★	★	---	★	---	5
Classen et al.[25]<br><br>2010, USA	★	★	★	★	---	★	★	---	6
Cooper et al.[26]<br><br>2010, UK	★	★	---	★	★	★	★	---	6
Reynolds et al.[27]<br><br>2010, UK	---	★	★	★	★	★	★	---	6
Al-Isa et al.[28]<br><br>2011,Kuwaiti	---	★	---	---	★	★	★	---	4
Hochner et al. [29]<br><br>2012, Israel	★	★	★	★	★	★	★	---	7

Johnson et al.[30] 2012,UK	★	★	★	★	★	★	★	---	7
Murrin et al.[31] 2012, Ireland*	★	★	---	★	---	---	★	---	4
Wang et al.[32] 2012, China	★	★	---	★	★	★	★	★	7
Hu et al.[33] 2013, China	★	★	★	★	★	★	★	---	7
Magarey et al.[16] 2003,Australia	★	★	★	★	★	★	★	---	7
Kelly et al.[34] 2014,Ireland*	★	★	★	★	---	---	★	---	5
Cho et al.[36] 2015,USA	---	★	---	---	---	---	---	---	1
Derraik et al. [37] 2015,Sweden	★	★	★	★	★	★	★	---	7
Han et al[38] 2015,Scottland	★	★	★	★	★	★	★	---	7
Alati et al.[40] 2016,Australia	★	★	★	★	★	★	★	---	7

Zalbahar et al.[41] 2016,Australia	★	★	---	★	★	★	★	★	7
Rath et al.[42] 2016,Australia	★	★	---	★	★	★	★	---	6
Swanton et al.[44] 2017,USA	★	★	★	★	★	★	★	---	7
Rodrigo M. Carrillo-Larco et al.[45] 2018,Peru	★	★	★	★	★	★	★	---	7
Kaseva et al.[46] 2018,Finland	---	★	★	★	---	★	★	---	5
Schoppa et al.[47] 2018,Finland	★	★	★	★	★	★	★	---	7

Reference

1. Khoury P, Morrison JA, Laskarzewski PM, Glueck CJ. Parent-offspring and sibling body mass index associations during and after sharing of common household environments: the Princeton School District Family Study. *Metabolism.* 1983;32(1):82-89.
2. Friedlander Y, Kark J, Kaufmann N, Berry E, Stein YJIjoo. Familial aggregation of body mass index in ethnically diverse families in Jerusalem. The Jerusalem Lipid Research Clinic. 1988;12(3):237-247.
3. Rotimi C, Cooper R. Familial resemblance for anthropometric measurements and relative fat distribution among African Americans. *International journal of obesity and related metabolic disorders: journal of the International Association for the Study of Obesity.* 1995;19(12):875.
4. Lake JK, Power C, Cole TJ. Child to adult body mass index in the 1958 British birth cohort: associations with parental obesity. *Archives of disease in childhood.* 1997;77(5):376-380.
5. Al-Isa AN. Factors associated with overweight and obesity among Kuwaiti college women. *Nutrition and health.* 1998;12(4):227-233.
6. Burke V, Beilin LJ, Dunbar D. Family lifestyle and parental body mass index as predictors of body mass index in Australian children: a longitudinal study. *International journal of obesity.* 2001;25(2):147-157.
7. Milligan R, Burke V, Beilin L, et al. Influence of gender and socio-economic status on dietary patterns and nutrient intakes in 18-year-old Australians. *Australian and New Zealand journal of public health.* 1998;22(4):485-493.
8. Williams S. Overweight at age 21: the association with body mass index in childhood and adolescence and parents' body mass index. A cohort study of New Zealanders born in 1972–1973. *International journal of obesity.* 2001;25(2):158-163.
9. Laitinen J, Power C, Järvelin M-R. Family social class, maternal body mass index, childhood body mass index, and age at menarche as predictors of adult obesity. *The American journal of clinical nutrition.* 2001;74(3):287-294.
10. Magnusson P, Rasmussen F. Familial resemblance of body mass index and familial risk of high and low body mass index. A study of young men in Sweden. *International journal of obesity.* 2002;26(9):1225-1231.
11. Salces I, Rebato E, San Martin L, Rosique J, Vinagre A, Susanne C. Family resemblance for anthropometric traits II. Assessment of maternal occupational and age effects. *Homo.* 2002;52(3):201-213.
12. Mirmiran P, Mirbolooki M, Azizi F. Familial clustering of obesity and the role of nutrition: Tehran Lipid and Glucose Study. *International journal of obesity.* 2002;26(12):1617-1622.
13. Kvaavik E, Tell GS, Klepp K-I. Predictors and tracking of body mass index from adolescence into adulthood: follow-up of 18 to 20 years in the Oslo Youth Study. *Archives of pediatrics & adolescent medicine.* 2003;157(12):1212-1218.
14. Wu D-M, Hong Y, Sun C-A, Sung P-K, Rao D, Chu N-F. Familial resemblance of adiposity-related parameters: results from a health check-up population in Taiwan. *European journal of epidemiology.* 2003;18(3):221-226.
15. Magarey AM, Daniels LA, Boulton TJ, Cockington RA. Predicting obesity in early adulthood from childhood and parental obesity. *International journal of obesity.* 2003;27(4):505-513.
16. Kazumi T, Kawaguchi A, Yoshino G. Associations of middle-aged mother's but not father's body mass index with 18-year-old son's waist circumferences, birth weight, and serum hepatic enzyme levels. *Metabolism.* 2005;54(4):466-470.
17. Crossman A, Sullivan DA, Benin M. The family environment and American adolescents' risk of obesity as young adults. *Social science & medicine.* 2006;63(9):2255-2267.
18. Kivimäki M, Lawlor DA, Smith GD, et al. Substantial intergenerational increases in body mass index are not explained by the fetal overnutrition hypothesis: the Cardiovascular Risk in Young Finns Study. *The American journal of clinical nutrition.* 2007;86(5):1509-1514.
19. Abu-Rmeileh NM, Hart CL, McConnachie A, Upton MN, Lean ME, Watt GC. Contribution of Midparental BMI and other determinants of obesity in adult offspring. *Obesity (Silver Spring, Md).* 2008;16(6):1388-1393.

20. Koupil I, Toivanen P. Social and early-life determinants of overweight and obesity in 18-year-old Swedish men. *International journal of obesity (2005).* 2008;32(1):73-81.
21. Tequeanes ALL, Gigante DP, Assunção MCF, Chica DAG, Horta BL. Maternal anthropometry is associated with the body mass index and waist: height ratio of offspring at 23 years of age. *The Journal of nutrition.* 2009;139(4):750-754.
22. Kowaleski-Jones L, Brown BB, Fan JX, Smith KR, Zick CD. Are you what your mother weighs? Evaluating the impact of maternal weight trajectories on youth overweight. *Maternal and child health journal.* 2010;14(5):680-686.
23. Classen TJ. Measures of the intergenerational transmission of body mass index between mothers and their children in the United States, 1981–2004. *Economics & Human Biology.* 2010;8(1):30-43.
24. Cooper R, Hyppönen E, Berry D, Power C. Associations between parental and offspring adiposity up to midlife: the contribution of adult lifestyle factors in the 1958 British Birth Cohort Study. *The American journal of clinical nutrition.* 2010;92(4):946-953.
25. Reynolds R, Osmond C, Phillips D, Godfrey K. Maternal BMI, parity, and pregnancy weight gain: influences on offspring adiposity in young adulthood. *The Journal of Clinical Endocrinology & Metabolism.* 2010;95(12):5365-5369.
26. Naser Al-Isa A, Campbell J, Desapriya E. Factors associated with overweight and obesity among Kuwaiti men. *Asia Pacific Journal of Public Health.* 2013;25(1):63-73.
27. Hochner H, Friedlander Y, Calderon-Margalit R, et al. Associations of maternal prepregnancy body mass index and gestational weight gain with adult offspring cardiometabolic risk factors: the Jerusalem Perinatal Family Follow-up Study. *Circulation.* 2012;125(11):1381-1389.
28. Johnson PC, Logue J, McConnachie A, et al. Intergenerational change and familial aggregation of body mass index. *Eur J Epidemiol.* 2012;27(1):53-61.
29. Murrin CM, Kelly GE, Tremblay RE, Kelleher CC. Body mass index and height over three generations: evidence from the Lifeways cross-generational cohort study. *BMC public health.* 2012;12(1):81.
30. Wang H, Wang J, Liu M-M, et al. Epidemiology of general obesity, abdominal obesity and related risk factors in urban adults from 33 communities of Northeast China: the CHPSNE study. *BMC public health.* 2012;12(1):967.
31. Hu Y, He L, Wu Y, Ma G, Li L, Hu Y. Familial correlation and aggregation of body mass index and blood pressure in Chinese Han population. *BMC public health.* 2013;13(1):686.
32. Kelly GE, Murrin C, Viljoen K, O'Brien J, Kelleher C. Body mass index is associated with the maternal lines but height is heritable across family lines in the Lifeways Cross-Generation Cohort Study. *BMJ open.* 2014;4(12).
33. Cho C, Maawadh A, Gerstner GE. Comparisons of chewing rhythm, craniomandibular morphology, body mass and height between mothers and their biological daughters. *Archives of Oral Biology.* 2015;60(11):1667-1674.
34. Derraik JG, Ahlsson F, Diderholm B, Lundgren M. Obesity rates in two generations of Swedish women entering pregnancy, and associated obesity risk among adult daughters. *Scientific reports.* 2015;5:16692.
35. Han T, Hart C, Haig C, et al. Contributions of maternal and paternal adiposity and smoking to adult offspring adiposity and cardiovascular risk: the Midspan Family Study. *BMJ open.* 2015;5(11).
36. Alati R, Betts K, Williams G, Najman J, Zalbahar N, Mamun A. Generational increase in obesity among young women: a prospective analysis of mother–daughter dyads. *International Journal of Obesity.* 2016;40(1):176-180.
37. Zalbahar N, Najman J, McIntrye HD, Mamun A. Parental pre-pregnancy BMI influences on offspring BMI and waist circumference at 21 years. *Australian and New Zealand journal of public health.* 2016;40(6):572-578.
38. Rath S, Marsh JA, Newnham JP, et al. Parental pre-pregnancy BMI is a dominant early-life risk factor influencing BMI of offspring in adulthood. *Obesity science & practice.* 2016;2(1):48-57.

39. Swanton S, Choh AC, Lee M, et al. Body mass index associations between mother and offspring from birth to age 18: the Fels Longitudinal Study. *Obesity Science & Practice.* 2017;3(2):127-133.
40. Carrillo-Larco RM, Bernabé-Ortiz A, Sal y Rosas VG, et al. Parental body mass index and blood pressure are associated with higher body mass index and blood pressure in their adult offspring: a cross-sectional study in a resource-limited setting in northern Peru. *Tropical Medicine & International Health.* 2018;23(5):533-540.
41. Kaseva N, Vääräsmäki M, Matinolli H, et al. Pre-pregnancy overweight or obesity and gestational diabetes as predictors of body composition in offspring twenty years later: evidence from two birth cohort studies. *International Journal of Obesity.* 2018;42(4):872-879.
42. Schoppa I, Lyass A, Heard-Costa N, et al. Association of Maternal Prepregnancy Weight with Offspring Adiposity Throughout Adulthood over 37 Years of Follow-up. *Obesity.* 2019;27(1):137-144.
43. Sørensen T, Holst C, Stunkard AJ, Skovgaard LTJIjoo, Obesity rmdjotIAftSo. Correlations of body mass index of adult adoptees and their biological and adoptive relatives. 1992;16(3):227-236.
44. Stuebe AM, Forman MR, Michels KB. Maternal-recalled gestational weight gain, pre-pregnancy body mass index, and obesity in the daughter. *International journal of obesity.* 2009;33(7):743-752.
45. Chaparro MP, Koupil I, Byberg L. Maternal pre-pregnancy BMI and offspring body composition in young adulthood: the modifying role of offspring sex and birth order. *Public health nutrition.* 2017;20(17):3084-3089.
47. Eriksson JG, Sandboge S, Salonen M, Kajantie E, Osmond C. Maternal weight in pregnancy and offspring body composition in late adulthood: findings from the Helsinki Birth Cohort Study (HBCS). *Annals of medicine.* 2015;47(2):94-99.
48. Vik KL, Romundstad P, Carslake D, Davey Smith G, Nilsen TI. Comparison of father-offspring and mother-offspring associations of cardiovascular risk factors: family linkage within the population-based HUNT Study, Norway. *International journal of epidemiology.* 2014;43(3):760-771

Appendix

Search strategies

Adapted Newcastle–Ottawa scale for cohort studies

MOOSE Checklist for Meta-analyses of observational studies

Search strategy

PubMed	#1,"Search ((((((((body mass index[MeSH Terms]) OR body weight[MeSH Terms]) OR body size[MeSH Terms]) OR body composition[MeSH Terms]) OR body constitution[MeSH Terms]) OR adiposity[MeSH Terms]) OR overweight[MeSH Terms]) OR obesity[MeSH Terms]) OR bmi[Title/Abstract] Sort by: [pubsolr12]"	717,217
	#2,"Search ((((((offspring[Title/Abstract]) OR adult child[Title/Abstract]) OR adult children) OR adolescent) OR adult daughter) OR adult son) OR young adult Sort by: [pubsolr12]"	2,634,623
	#3 ,"Search (((((((((parental[Title/Abstract]) OR maternal[Title/Abstract]) OR prepregnancy[Title/Abstract]) OR pre-pregnancy[Title/Abstract]) OR father[Title/Abstract]) OR mother[Title/Abstract]) OR parents[Title/Abstract]) OR male[Title/Abstract]) OR female[Title/Abstract]) OR paternal[Title/Abstract] Sort by: [pubsolr12]"	1,736,959
	#4,"Search (#2) AND #3 Sort by: [pubsolr12]"	145,586 (521,542)
	#5,"Search (#1) AND #4 Sort by: [pubsolr12]"	137,988
		(51,109)
	#6,"Search (#5) AND #6 Sort by: [relevance]",	39,253
	#7,"Search (#5) AND #6 Filters: Humans Sort by: [pubsolr12]",	32,689
		46,366
EmBase	#1 ('body mass index':ab,ti OR 'body weight':ab,ti OR 'body size':ab,ti OR 'body composition':ab,ti OR 'body constitution':ab,ti OR adipsoity:ab,ti OR obesity:ab,ti OR overweight:ab,ti OR bmi:ab,ti) AND [1980-2020]/py	980,121

#2 (offspring:ab,ti OR 'adult child':ab,ti OR 'adult son':ab,ti OR 'adult daughter':ab,ti OR 'young adult':ab,ti OR 'old adult':ab,ti OR 'adolescents'/exp OR adolescents) AND [1980-2020]/py	383,832
#3 (parental:ab,ti OR maternal:ab,ti OR paternal:ab,ti OR parents:ab,ti OR father:ab,ti OR mother:ab,ti OR male:ab,ti OR female:ab,ti OR prepregnancy:ab,ti) AND [1980-2020]/py	2,6664,995
#4 #1 AND #2	16,410
#5 #1 AND #3	236,677
#6 #4 AND #5	12,006
Total	32,689

Adapted Newcastle–Ottawa scale for cohort studies [1]

Selection

1) Representativeness of the exposed cohort [1]

a) truly representative of the average maternal OR paternal population[a] in the community □

b) somewhat representative of the average maternal OR paternal population[a] in the community

c) selected group of users

d) no description of the derivation of the cohort

2) Selection of the non-exposed cohort [1]

a) drawn from the same community as the exposed cohort □

b) drawn from a different source

c) no description of the derivation of the non exposed cohort

3) Ascertainment of exposure [2]

a) secure record (e.g. explicitly measured weight and height [a]) □

b) structured interview (e.g. validated self-reported[a])□

c) any self-report

d) no description

4) Demonstration the outcome of interest was not present at start of the study

a) yes □

b) no

Comparability

5) Comparability of cohorts on the basis of the design or analysis [3]

a) study controls for age[a] □

b) study controls for any additional factor □

c) no factors controlled for[a]

Outcome (note: outcome is child weight status)

6) Assessment of outcome [2]

a) independent blind assessment □

b) record linkage □

c) self-report

d) no description

7) Was follow-up long enough for outcomes to occur [2]

a) yes (select an adequate follow up period for outcome of interest: all children in the analysis were the target age of the research question[a]) □

b) no

8) Adequacy of follow up of cohorts [1]

a) complete follow up - all subjects accounted for □

b) subjects lost to follow up unlikely to introduce bias - small number lost – >80%[a] follow up, or description provided of those lost) □

c) follow up rate < 80%[a] and no description of those lost

d) no statement

Note: A study can be awarded a maximum of one star for each numbered item within the Selection and Outcome categories. A maximum of two stars can be given for Comparability.

Footnote: [a]Red font is where form was adapted to make questions relevant to this systematic review.

[1]Questions assessing selection bias; [2]Questions assessing information bias; [3]Questions assessing confounding

MOOSE[2] Checklist for Meta-analyses of Observational Studies

Item No	Recommendation	Reported in manuscript section/paragraph number
Reporting of background should include		
1	Problem definition	Introduction/1,2
2	Hypothesis statement	Introduction/2
3	Description of study outcome(s)	Methods/3,6
4	Type of exposure or intervention used	Methods/3
5	Type of study designs used	Methods/3
6	Study population	Methods/3
Reporting of search strategy should include		
7	Qualifications of searchers (eg, librarians and investigators)	Methods/5
8	Search strategy, including time period included in the synthesis and key words	Methods/2<br>Fig S1
9	Effort to include all available studies, including contact with authors	Methods/2<br>Results/1
10	Databases and registries searched	Methods/2<br>Fig S1

11	Search software used, name and version, including special features used (eg, explosion)	Methods/2<br>Appendix
12	Use of hand searching (eg, reference lists of obtained articles)	Methods/2
13	List of citations located and those excluded, including justification	Results/1<br>Fig S1
14	Method of addressing articles published in languages other than English	Methods/3
15	Method of handling abstracts and unpublished studies	Methods/3
16	Description of any contact with authors	NA
Reporting of methods should include		
17	Description of relevance or appropriateness of studies assembled for assessing the hypothesis to be tested	Methods/6-8
18	Rationale for the selection and coding of data (eg, sound clinical principles or convenience)	Methods/6-9
19	Documentation of how data were classified and coded (eg, multiple raters, blinding and interrater reliability)	Methods/5
20	Assessment of confounding (eg, comparability of cases and controls in studies where appropriate)	Methods/11<br>Fig S3
21	Assessment of study quality, including blinding of quality assessors, stratification or regression on possible predictors of study results	Methods/12<br>Table S19
22	Assessment of heterogeneity	Methods/8,10<br>Tables 12-16

23	Description of statistical methods (eg, complete description of fixed or random effects models, justification of whether the chosen models account for predictors of study results, dose-response models, or cumulative meta-analysis) in sufficient detail to be replicated	Methods/6-8
24	Provision of appropriate tables and graphics	Fig 1-3<br>Fig S1-S17<br>Table S1-S19
Reporting of results should include		
25	Graphic summarizing individual study estimates and overall estimate	Fig 1-3<br>Fig S1-S8
26	Table giving descriptive information for each study included	Table S1
27	Results of sensitivity testing (eg, subgroup analysis)	Results/8-10<br>Tables 12-18<br>Fig S9-S13
28	Indication of statistical uncertainty of findings	Confidence intervals provided throughout narrative results, tables and figures in the main manuscript and in Supplementary Tables and Figures, and Discussion/3: discussion of limitations

Reporting of discussion should include		
29	Quantitative assessment of bias (eg, publication bias)	Results/14-15<br>Fig S17
30	Justification for exclusion (eg, exclusion of non-English language citations)	Discussion/3
31	Assessment of quality of included studies	Results/14-15
Reporting of conclusions should include		
32	Consideration of alternative explanations for observed results	Discussion/1-3
33	Generalization of the conclusions (ie, appropriate for the data presented and within the domain of the literature review)	Discussion/1
34	Guidelines for future research	Discussion/1, 6
35	Disclosure of funding source	Conflict of interest

Reference:

1. Wells G, Shea B. Newcastle-Ottawa quality assessment scale cohort studies. In:2000.
2. Stroup D, Berlin J, Morton S. MOOSE Checklist for Meta-analyses of Observational Studies. *Jama.* 2000;28315:14-15.
